# Augmenting Large Language Model With Prompt Engineering and Supervised Fine-Tuning in Non-Small Cell Lung Cancer Tumor-Node-Metastasis Staging: Framework Development and Validation

**DOI:** 10.2196/77988

**Published:** 2026-04-15

**Authors:** Ruonan Jin, Chao Ling, Yixuan Hou, Yuhan Sun, Ning Li, Jiefei Han, Jin Sheng, Qizhao Wang, Yuepeng Liu, Shen Zheng, Xingyu Ren, Chiyu Chen, Jue Wang, Cheng Li

**Affiliations:** 1 Liangyihui Network Technology Co, Ltd, 9/F, Tower T2, Jinheshangcheng, 140 Tianlin Road, Xuhui District, Shanghai, Shanghai, 200233, China, 86 13636599645; 2 School of Medicine, Tongji University, Shanghai, Shanghai, China; 3 Department of Thoracic Surgery, Renmin Hospital of Wuhan University, Wuhan, Hubei, China; 4 Department of Neuro-oncology, Neurosurgery Center, Beijing Tiantan Hospital, Capital Medical University, Beijing, Beijing, China; 5 Department of Medical Oncology, Sir Run Run Shaw Hospital, School of Medicine, Zhejiang University, Hangzhou, Zhejiang, China; 6 NoDesk AI Technology Co.,Ltd, Hangzhou, Zhejiang, China; 7 Zhipu AI Technology Co, Ltd, Beijing, Beijing, China; 8 Department of Biomedical Engineering, Faculty of Engineering, National University of Singapore, Singapore, Singapore, Singapore

**Keywords:** large language models, clinical decision support, non-small cell lung cancer, TNM staging, artificial intelligence, AI, GLM-32B, GPT-4o, prompt engineering, supervised fine tuning, diagnostic standardization, grassroots health care improvement

## Abstract

**Background:**

Accurate tumor node metastasis (TNM) staging is fundamental for treatment planning and prognosis in non-small cell lung cancer (NSCLC). However, its complexity poses significant challenges. Traditional rule-based natural language processing methods are constrained by their reliance on manually crafted rules and are susceptible to inconsistencies in clinical reporting.

**Objective:**

This study aimed to develop and validate a robust, accurate, and operationally efficient artificial intelligence framework for the TNM staging of NSCLC by strategically enhancing a large language model, GLM-4-Air (general language model), through advanced prompt engineering and supervised fine-tuning (SFT).

**Methods:**

We constructed a curated dataset of 492 deidentified real-world medical imaging reports, with TNM staging annotations rigorously validated by senior physicians according to the AJCC (American Joint Committee on Cancer) 8th edition guidelines. The GLM-4-Air model was systematically optimized via a multi-phase process: iterative prompt engineering incorporating chain-of-thought reasoning and domain knowledge injection for all staging tasks, followed by parameter-efficient SFT using low-rank adaptation for the reasoning-intensive primary tumor characteristics (T) and regional lymph node involvement (N) staging tasks. The final hybrid model was evaluated on a completely held-out test set (black-box) and benchmarked against GPT-4o using standard metrics, statistical tests, and a clinical impact analysis of staging errors.

**Results:**

The optimized hybrid GLM-4-Air model demonstrated reliable performance. It achieved higher staging accuracies on the black-box test set: 92% (95% CI 0.850‐0.959) for T, 86% (95% CI 0.779‐0.915) for N, 92% (95% CI 0.850‐0.959) for distant metastasis status (M), and 90% for overall clinical staging; by comparison, GPT-4o attained 87% (95% CI 0.790‐0.922), 70% (95% CI 0.604‐0.781), 78% (95% CI 0.689‐0.850), and 80%, respectively. The model’s robustness was further evidenced by its macro-average *F*_1_-scores of 0.914 (T), 0.815 (N), and 0.831 (M), consistently surpassing those of GPT-4o (0.836, 0.620, and 0.698). Analysis of confusion matrices confirmed the model’s proficiency in identifying critical staging features while effectively minimizing false negatives. Crucially, the clinical impact assessment showed a substantial reduction in severe category I errors, which are defined as misclassifications that could significantly influence subsequent clinical decisions. Our model committed 0 category I errors in M staging and fewer category I errors in T and N staging. Furthermore, the framework demonstrated practical deployability, achieving efficient inference on consumer-grade hardware (eg, 4 RTX 4090 GPUs) with latencies suitable and acceptable for clinical workflows.

**Conclusions:**

The proposed hybrid framework, integrating structured prompt engineering and applying SFT to reasoning-heavy tasks (T/N), enables the GLM-4-Air model to serve as a highly accurate, clinically reliable, and cost-efficient solution for automated NSCLC TNM staging. This work demonstrates the efficacy and potential of a domain-optimized smaller model compared with an off-the-shelf generalist model, holding promise for enhancing diagnostic standardization in resource-aware health care environments.

## Introduction

On February 2, 2024, the National Cancer Center of China released “Cancer incidence and mortality in China, 2022.” The latest data indicate that lung cancer remains the leading cause of cancer incidence and mortality in China [1]. Lung cancer can be divided into 2 main histopathological types: non-small cell lung cancer (NSCLC) and small cell lung cancer. According to statistical data, NSCLC accounts for approximately 85% of all cases [2].

The tumor node metastasis (TNM) staging system, developed by the American Joint Committee on Cancer (AJCC), serves as the foundation for NSCLC treatment planning, outcome assessment, and clinical research design [3]. It evaluates 3 critical parameters: primary tumor characteristics (T), regional lymph node involvement (N), and distant metastasis status (M) [4].

Although tumor characteristics are described in medical imaging reports, the explicit TNM stage classification is rarely included [5]. Physicians must extract and interpret the relevant information from the reports and apply the complex TNM stage classification criteria to determine the stage of the patients. The intricacy of the criteria makes it challenging even for experienced physicians to memorize all the details, posing an even greater challenge for early-career physicians or those in primary care settings.

NLP approaches reported in the TNM stage classification primarily consist of rule-based methods and deep learning methods. Among the reported research, rule-based NLP approaches have achieved a maximum accuracy of approximately 85% for T and N staging [6,7]. For M staging, a peak accuracy of about 93% has been reached by Park et al [8] using a deep learning NLP method.

While progress has been made, NLP approaches extensively rely on intricately crafted rules [6,7,9] and the annotated datasets of substantial size [8,10]. Besides, the overall accuracy is heavily affected by report standardization, terminology consistency, and contextual clarity in clinical documentation [7].

Recently, large language models (LLMs) have become a hot topic due to their ability to capture complex patterns and structures of language without the above-mentioned limitations of traditional NLP [11-15].

In the study conducted by Nakamura et al [16], the performance of GPT-4 in extracting lung cancer staging from CT radiological reports was evaluated. When guided by TNM staging rules, the model demonstrated varying accuracy rates: 52.2% for T staging, 78.9% for N staging, and 86.7% for M staging without extra training. They indicated that most of ChatGPT’s errors were caused by challenges with numerical reasoning or insufficiency in anatomical or lexical knowledge.

Another study by Matsuo et al [17] investigated the performance of multilingual LLMs in interpreting TNM staging from both Japanese and English radiological reports. The researchers found that providing comprehensive TNM definitions notably improved the model’s accuracy. The highest accuracy was achieved for reports in English: 47% for T staging, 80% for N staging, 94% for M staging, and an overall accuracy of 36%.

Additionally, based on a fine-tuned method, a study by Fujimoto [18] developed an LLM that was fine-tuned on an augmented dataset consisting of 27 lung cancer cases. They noted that fine-tuning can help the model understand the relationships between different aspects of TNM classification, such as how tumor size relates to the T stage and how lymph node involvement affects the N stage. They also observed that the T stage contains several types of criteria other than tumor size and is therefore more complex compared with the M stage.

Previous applications of LLMs in NSCLC TNM staging have demonstrated promising performance in N and M stage classification. However, these methods show limited accuracy in T staging, which requires a more precise assessment of tumor characteristics, including size, invasion depth, and local extension. This underscores the necessity for further research to develop more robust methods for analyzing complex tumor characteristics [16-21].

Moreover, the clinical implementation of artificial intelligence (AI) technologies necessitates thorough cost-effectiveness evaluation, particularly as data privacy concerns often mandate local (on-premises) deployment. The substantial operational costs associated with many commercial LLMs present a dual challenge: high application programming interface fees for cloud-based services and, for local deployment, the significant hardware investment required to run large-parameter models, which often exceed the capabilities of consumer-grade equipment. These cost barriers critically impact the feasibility of integrating such technologies into routine clinical practice.

Given the challenges encountered in prior studies, this research focuses on optimizing GLM-4-Air (general language model), an LLM with 32 billion parameters, for a more robust and cost-effective solution to NSCLC TNM stage classification. The training framework we developed specifically aims to improve TNM staging accuracy and efficiency for NSCLC, and its performance was rigorously evaluated using a held-out internal test set to ensure an unbiased assessment within this study’s scope. The ultimate goal is to enhance the standardization of NSCLC cancer management in grassroots medical settings in mainland China as well as in other developing countries.

## Methods

### Overview

**Figure 1.**
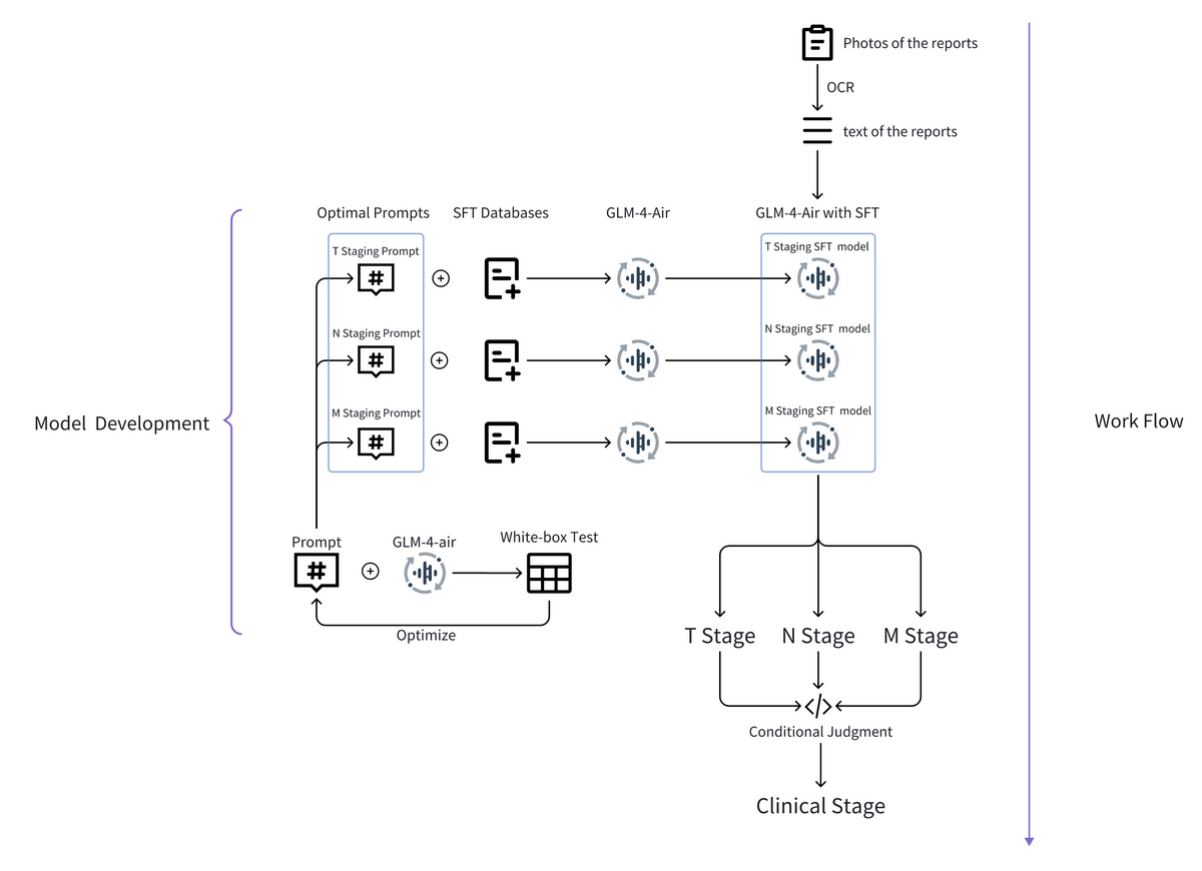
The comprehensive workflow from model development to clinical application. GLM: general language model; M: distant metastasis status; N: regional lymph node involvement; OCR: optical character recognition; SFT: supervised fine-tuning; T: primary tumor characteristics.

This study used a structured pipeline that systematically progresses from raw medical report processing to final clinical staging output, as illustrated in Figure 1. This end-to-end framework facilitates reproducible model development through a structured transformation of unstructured reports into staging conclusions that adhere to clinical standards.

### Ethical Considerations

This study was approved by the Shanghai Ethics Committee for Clinical Research (SECCR2025-260) as a retrospective analysis. The requirement for informed consent was waived due to the retrospective nature of the study and the use of deidentified data. All patient information was anonymized prior to analysis, with all identifiers, medical record number/visit number, hospital name, attending physician’s name, examining doctor’s name, and other personally identifiable information such as ID number and contact details removed, to ensure privacy and confidentiality. No compensation was provided to participants as this was a retrospective study using existing clinical data. The study did not involve any images that could potentially identify individual participants. This research was conducted in accordance with the principles of the Declaration of Helsinki and adhered to all relevant regional and national research ethics guidelines.

### Dataset

#### Data Collection

The data for this study were collected through our proprietary medical record management platform, which was specifically developed to assist patients in managing their medical records. The study enrolled participants who were initially suspected of lung cancer through imaging or clinical presentation and subsequently received histopathological or cytological confirmation of NSCLC. All included cases had undergone relevant clinical management between January 2018 and May 2025, with available medical records containing extractable key elements essential for TNM staging, such as tumor size, location, nodal status, and metastatic findings.

The finalized dataset comprised photos of medical imaging reports for up to 492 cases of NSCLC. Using optical character recognition (OCR) technology provided by Tencent Co Ltd [22], we converted the captured images into text format. Following the OCR process, we applied no further processing to enhance the text’s quality. Nevertheless, we specifically examined the OCR outputs of both white-box and black-box and ran an automated post-OCR Chinese character error rate test measured on the 27 reports randomly selected from both white-box and black-box.

#### Data Annotation

The annotation process was conducted by 2 expert-level lung cancer physicians, who performed 3 independent rounds of detailed TNM staging annotations in accordance with the 8th edition AJCC TNM classification guidelines. For the definition and interpretation of professional medical terms, we referred to the standards of the Union for International Cancer Control. All annotators were blinded to the previously annotated results to ensure objectivity. Discrepancies in the annotations were systematically documented and discussed with additional physicians to reach a consensus. Finally, high-quality gold standard annotations for subsequent model development and evaluation were established.

In addition, to quantify the degree of interannotator agreement and the reliability of annotations, which serves as a human-performance benchmark for calibrating model performance, we calculated the Cohen kappa coefficient for all 3 stages in both white-box and black-box test sets among the clinical annotators, specifically prior to the adjudication process.

#### Dataset Splitting

**Figure 2.**
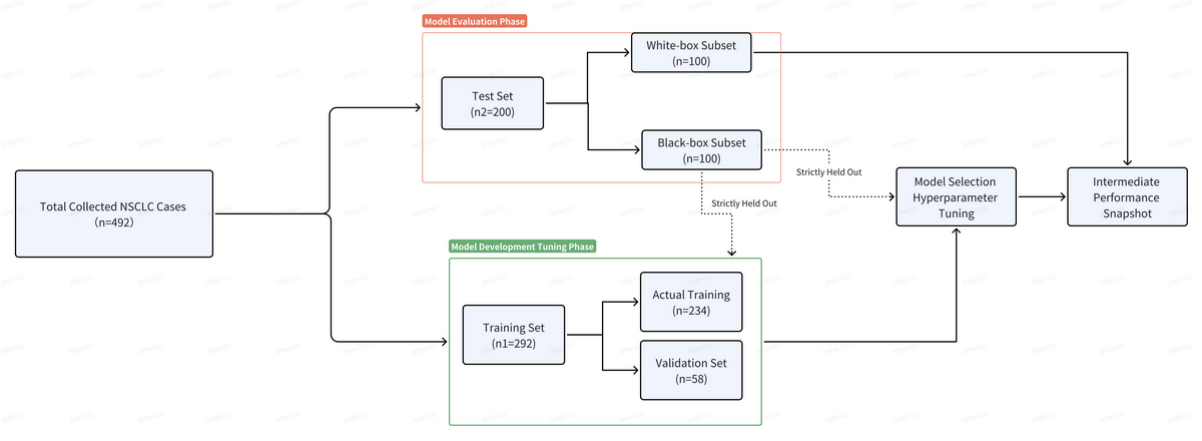
The overall procedure of dataset splitting in this study. NSCLC: non-small cell lung cancer.

The overall procedure of dataset splitting is shown in Figure 2. We randomly divided all 492 collected and annotated cases into training (n1=292) and test (n2=200) datasets. For hyperparameter tuning (including learning rate selection) and early stopping, we strictly used a dedicated validation set. This set was randomly held out from the original 292-case training dataset (n1), constituting about 20% (58 cases) of it.

The test dataset (n2) was further subdivided into a white-box subset for model development and a black-box subset for final performance evaluation (100 cases each). It is important to clarify that both subsets were derived from the same internal pool of user-uploaded cases within our platform. The term “black-box” specifically refers to this subset’s role as a rigorously held-out, internal test set that remained completely inaccessible during all phases of model development and tuning, thereby ensuring an unbiased final assessment. It was not an external validation cohort from distinct institutions or independent collection pipelines. A stratified sampling strategy was used during data partitioning to ensure a balanced representation of all stages in both subsets, thereby supporting a robust and generalizable evaluation of model performance. The white-box subset was kept completely separate and was never used for any training or tuning decisions. Its role was exclusively for intermediate evaluation at the end of the development cycle to provide a single, neutral performance snapshot and preliminary model diagnosis.

#### Data Distribution

The dataset characteristics and stratification are summarized as follows and detailed in the Multimedia Appendices. As detailed in Multimedia Appendix 1, the imaging modality distribution (n=492) was 84.76% CT, 13.01% PET-CT, with the remainder comprising other modalities (eg, MRI, Ultrasound). Regarding hospital sources, 78% of 492 cases originated from 51 distinct contributing hospitals, while the source was unidentified for the remaining 22% due to users’ proactive removal of hospital identifiers for privacy protection during upload. Subsequently, Multimedia Appendix 2 presents the detailed class distribution for the T and N categories of the training set, and T, N, and M categories, along with the overall clinical stages of the test sets according to annotation. Both the training and test sets were designed to ensure coverage of all staging subcategories with a relatively balanced distribution.

### Criteria for TNM and Clinical Staging

In this study, to enhance the practical application of AJCC 8th Edition guidelines within Chinese clinical contexts, we developed supplementary TNM staging rules through expert clinical consultation. These rules address specific scenarios either not explicitly covered in the official guidelines or requiring disambiguation for Chinese medical terminology. A comprehensive mapping table (Table 1) documents each rule alongside its corresponding AJCC guideline reference. The supplementary rules primarily address: (1) Clinical interpretations for anatomically complex scenarios (eg, trans-lobar growth), and (2) Disambiguation of Chinese medical terms with overlapping semantics (eg, distinguishing between local invasion vs metastatic spread).

**Table 1.** Mapping of supplementary staging rules to the American Joint Committee on Cancer (AJCC) 8th edition guidelines.

Tumor node metastasis stage	AJCC guidelines	Supplementary rules
T^a^ (primary tumor)		
T0		
T0	No primary tumor	1. Maximum Tumor Diameter: In imaging reports, multiple tumor measurements may be present due to factors such as prior surgeries or treatments. The maximum tumor diameter is explicitly defined as the most recent measurement obtained following the current treatment cycle. Care should be taken to avoid misinterpreting size information from nonneoplastic nodules, such as “solid nodules.” 2. Only explicit expressions such as “vertebral metastasis,” “invasion,” “encroachment,” or “occupation” in the report should be considered indicators of tumor invasion. Other expressions should not be used as criteria for invasion. (Definition of “Tumor Invasion”) 3. A nodule should be considered a cancer nodule only if the report explicitly describes it as “malignant soft tissue” or an equivalent severe condition. For example, “a malignant soft tissue mass in the right middle lobe, with multiple nodules in both lungs, suggestive of metastasis.” Other less severe descriptions should not be used as criteria for the presence of a cancer nodule (Definition of “Cancer Nodule”).
Tis		
Tis	Carcinoma in situ (squamous or adenocarcinoma)	NULL
T1		
T1	Tumor ≤3 cm	NULL
T1a(mi)	Minimally invasive adenocarcinoma	NULL
T1a	Superficial spreading tumor in central airways^b^	NULL
T1a	Tumor ≤1 cm	NULL
T1b	Tumor >1 but ≤2 cm	NULL
T1c	Tumor >2 but ≤3 cm	NULL
T2		
T2	Tumor >3 but ≤5 cm or tumor involving: visceral pleura, main bronchus (not carina), atelectasis to hilum^c^	Tumor invades the left or right main bronchus or visceral pleura.
T2a	Tumor >3 but ≤4 cm	Note: This supplementary rule conveys the same definition as the AJCC guidelines, with the only modification being the explicit inclusion of “left” and “right” specifications, which are not explicitly specified in the original AJCC guidelines.
T2b	Tumor >4 but ≤5 cm	
T3	Tumor >5 but ≤7 cm or invading chest wall, pericardium, phrenic nerve; or separate tumor nodule(s) in the same lobe	1. Tumor size <5 cm with trans-lobar growth. 2. Tumor invades the parietal pleura (usually noted in pathological staining), chest wall (including Pancoast tumors). Note: Based on the AJCC guidelines, this supplementary rule adds only the scenario involving invasion of the parietal pleura, and the description of chest wall invasion has been expanded to include cases of Pancoast tumors. 3. Tumor invades the main stems of more distal arteries or veins. Note: Based on the AJCC guidelines, this supplementary criterion adds only the scenario involving invasion of the main stems of more distal arteries or veins.
T4	Tumor >7 cm or tumor invading: mediastinum, diaphragm, heart, great vessels, recurrent laryngeal nerve, carina, trachea, esophagus, spine; or tumor nodule(s) in a different ipsilateral lobe	1. Tumor size 5‐7 cm with trans-lobar growth. 2. Tumor invades major vessels (aorta, superior vena cava, inferior vena cava, main pulmonary arteries, and left or right pulmonary veins within the pericardium), vertebral bodies. Note: Based on the AJCC guidelines, this supplementary rule adds the definition of major blood vessels provided in parentheses and includes the scenario involving invasion of the vertebral bodies.
N^d^ (regional lymph nodes)		
N0	No regional node metastasis	1. Expressions such as “small nodules” or “mild fluorodeoxyglucose (FDG) uptake” should be interpreted with caution and should not be directly inferred as lymph node metastasis. Only explicit statements such as “lymph node metastasis,” “enlarged lymph nodes,” or “markedly abnormal lymph nodes” should be considered evidence of lymph node metastasis. (Definition of “Lymph Node Metastasis”) 2. PET-CT^e^ reports were prioritized for evaluation. If unavailable, contrast-enhanced CT reports were used as the secondary reference. In the absence of both PET-CT and contrast-enhanced CT reports, lymph node status was directly classified as undetermined.
N1	Metastasis in ipsilateral pulmonary or hilar nodes	NULL
N2	Metastasis in ipsilateral mediastinal or subcarinal nodes	NULL
N3	Metastasis in contralateral mediastinal, hilar, or supraclavicular nodes	NULL
M^f^ (distant metastasis)		
M0	No distant metastasis	1. The requirement for cautious interpretation of terms such as “小结节” (small nodule), “低密度结节影” (low-density nodular shadow), “致密影” (dense shadow), “高密度影” (high-density shadow), “强回声伴声影” (strong echo with acoustic shadow), “软化灶” (softened focus), and “条状强化影” (linear enhancement), preventing their direct classification as metastatic lesions. 2. To mainly consider the expressions of “转移性” (metastatic), “考虑转移” (consider metastasis), “疑似转移” (suspected metastasis), “密度增高影” (increased density shadow), or “高密度结节” (high-density nodule) in other distant organs, as definitive evidence for metastasis.
M1a	Malignant pleural or pericardial effusions or pleural or pericardial nodules or separate tumor nodule(s) in a contralateral lobe	Metastasis is classified as M1a if it is confined to the thoracic cavity and the report explicitly describes tumor involvement of the pleura or pericardium with metastatic nodules.
M1b	Single extrathoracic metastasis	NULL
M1c	Multiple extrathoracic metastases (1 or >1 organ)	NULL

a T: primary tumor characteristics.

b Superficial spreading tumor of any size but confined to the tracheal or bronchial wall.

c Atelectasis or obstructive pneumonitis extending to hilum; such tumors are classified as T2a if >3 and ≤4 cm, T2b if >4 and ≤5 cm. Pleural effusions that are cytologically negative, nonbloody, transudative, and clinically judged not to be due to cancer are excluded.

d N: regional lymph node involvement.

e PET-CT: positron emission tomography–computed tomography.

f M: distant metastasis status.

Note that all supplementary logic maintains consistency with AJCC principles while improving alignment with Chinese clinical documentation practices. These enhancements ensure more accurate staging outcomes without contradicting established guideline definitions.

In accordance with our supplementary staging rules, we adopted a conservative interpretation for M staging: lesions are classified as metastatic only when the report contains terminology with high specificity for metastasis (eg, “转移” and “考虑转移”). Descriptions such as “小结节” (small nodule) or “高密度影” (high-density shadow), among others, which are common but nonspecific in Chinese radiology reports, are not considered sufficient evidence on their own, though they may contribute to a metastatic inference when supported by additional contextual findings. This strategy is designed to minimize false positives. While this approach could theoretically increase false negatives, we conducted a comparative analysis using a more lenient interpretation strategy. This alternative approach removed key restrictive rules, including:

1. The requirement for cautious interpretation of terms such as “小结节” (small nodule), “低密度结节影” (low-density nodular shadow), “致密影” (dense shadow), “高密度影” (high-density shadow), “强回声伴声影” (strong echo with acoustic shadow), “软化灶” (softened focus), and “条状强化影” (linear enhancement), preventing their direct classification as metastatic lesions.
2. To mainly consider the expressions of “转移性” (metastatic), “考虑转移” (consider metastasis), “疑似转移” (suspected metastasis), “密度增高影” (increased density shadow), or “高密度结节” (high-density nodule) in other distant organs, as definitive evidence for metastasis.

The results of the comparison of 2 strategies on M staging are shown and discussed in the Clinical Impact Assessment subsection of the Model Evaluation section in Results and Discussion, respectively.

**Figure 3.**
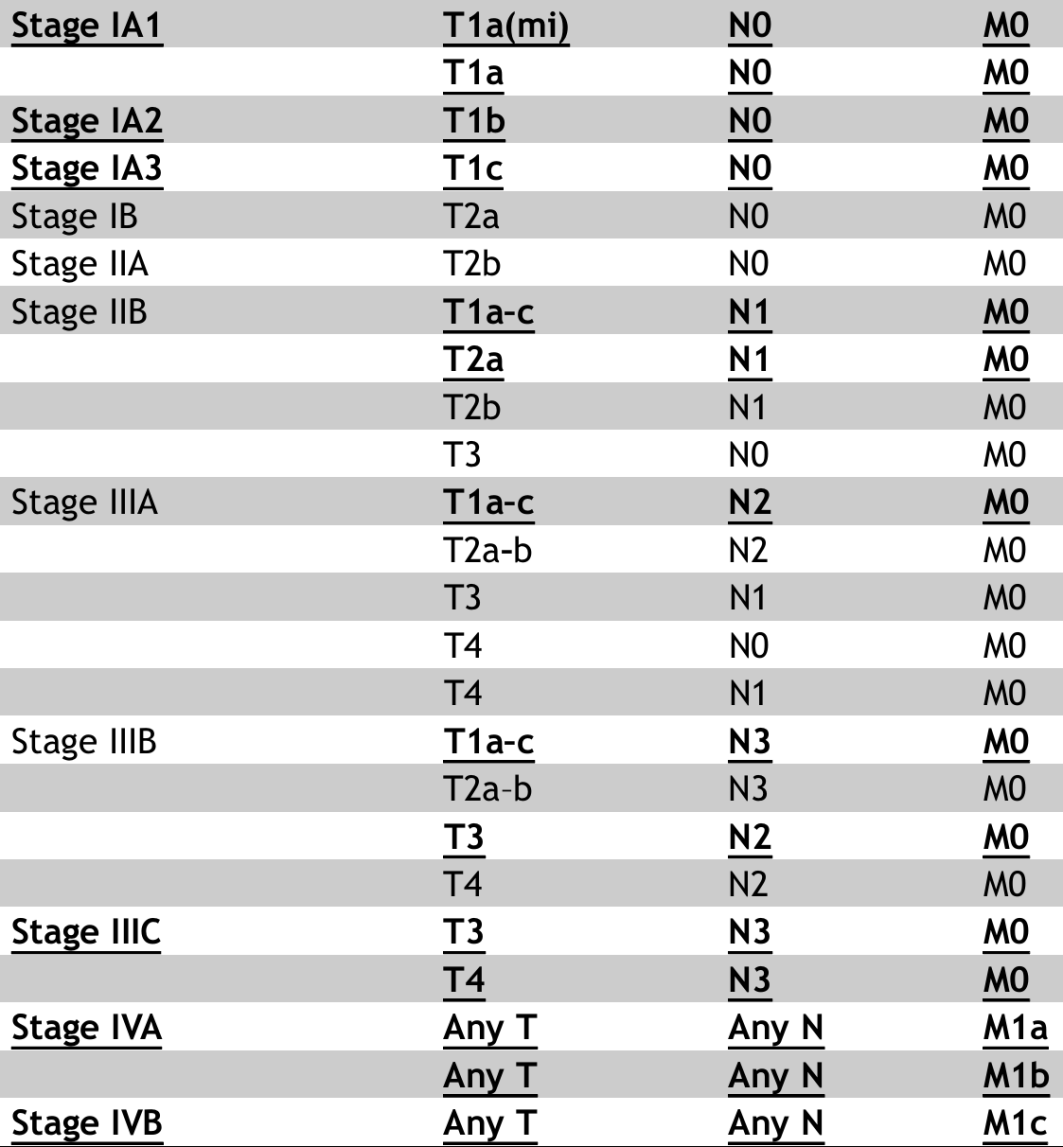
American Joint Committee on Cancer tumor node metastasis staging guidelines: From T, N, and M descriptors to stage grouping (adapted from Goldstraw et al [23], which is published under Creative Commons Attribution 4.0 International License [24]). M: distant metastasis status; N: regional lymph node involvement; T: primary tumor characteristics.

Finally, the overall clinical staging (Stage IA/IB, IIA/IIB, IIIA/IIIB/IIIC, or IVA/IVB) was algorithmically derived from the predicted T, N, and M staging results. This derivation strictly adhered to the stage grouping rules defined in the AJCC 8th Edition Cancer Staging Manual. The definitive mapping table from TNM combinations to clinical stages, as provided by the AJCC guidelines, is illustrated in Figure 3.

### Large Language Model

The GLM series, developed by Tsinghua University and Zhipu AI, has been pretrained on approximately 10 trillion tokens of multilingual data, predominantly in Chinese and English [25]. GLM-4-Air, an optimized variant of GLM-4 within the ChatGLM family, was selected for this study. This 32-billion-parameter model preserves the robust performance of its predecessor while offering reduced processing latency and lower inference costs, making it a more viable candidate for local deployment scenarios where data privacy is paramount, and the prohibitive hardware requirements of much larger models are a practical constraint.

### Prompt Optimization

#### Overview

To improve the performance of LLM in NSCLC TNM staging, we developed a series of prompts through iterative optimization. These prompts enhanced the model’s ability to accurately extract and interpret key patient medical information and match TNM judgment criteria.

The structure of our prompt implemented a standardized 3-step process: Information Extraction, Standard Matching, and Structured Output. This approach ensured consistent processing across all cases while enhancing the model’s reasoning capabilities. Notably, our output design incorporated the complete reasoning (chain-of-thought [CoT]) process rather than mere conclusions. This approach improved both reliability and interpretability of staging assessments, providing key information for result verification and analysis.

The entire optimization process was clearly divided into 4 phases, with each phase focusing on specific objectives and targeted improvements. Our strategy ensured systematic improvements at each stage, resulting in more precise and clinically applicable model performance.

#### Phase 1: Establishing a Baseline With Fundamental TNM Criteria

In the initial phase, a baseline prompt incorporating fundamental TNM staging criteria was developed using CoT reasoning. Through this structured approach, we established a robust foundation for the model to understand and process TNM staging tasks systematically. In this phase, we integrated the TNM staging criteria (Textbox 1) into the prompt, elaborating on the specific judgment logic as follows:

Textbox 1. **Tumor node metastasis (TNM) judgment criteria.**

Primary tumor characteristics (T) staging

 T4: Classified as T4 if the maximum tumor diameter exceeds 7 cm, if it invades specific anatomical structures, or if there are cancer nodules in different lobes of the same lung.

 T3: Classified as T3 if the maximum tumor diameter is greater than 5 cm and less than or equal to 7 cm, if it invades other specified structures, or if there are isolated cancer nodules within the same lobe.

 T2: Classified as T2 if the maximum tumor diameter is greater than 3 cm and less than or equal to 5 cm, if it invades the main bronchus or visceral pleura, or if there is tumor-induced atelectasis or obstructive pneumonia.

 T1:

  T1a: Classified as T1a if the maximum tumor diameter is less than or equal to 1 cm.

  T1b: Classified as T1b if the maximum tumor diameter is greater than 1 cm and less than or equal to 2 cm.  T1c: Classified as T1c if the maximum tumor diameter is greater than 2 cm and less than or equal to 3 cm.

  Tx: Classified as Tx if the primary tumor cannot be evaluated.

Regional lymph node involvement (N) staging

 N3: Classified as N3 if there is metastasis to contralateral mediastinal, contralateral hilar, ipsilateral or contralateral scalene, or supraclavicular lymph nodes.

 N2: Classified as N2 if there is metastasis to ipsilateral mediastinal lymph nodes or subcarinal lymph nodes.

 N1: Classified as N1 if there is metastasis to ipsilateral peribronchial lymph nodes and/or hilar lymph nodes, or if there is metastasis to ipsilateral intrapulmonary lymph nodes.

 N0: Classified as N0 if none of the above conditions are met.

 Nx: Classified as Nx if regional lymph node metastasis cannot be determined.

Distant metastasis status (M) staging

 M1c: Classified as M1c if there are multiple metastases outside the thorax.

 M1b: Classified as M1b if there is a single metastasis outside the thorax.

 M1a: Classified as M1a if there is intrathoracic metastasis, metastasis to the contralateral lung, tumor accompanied by metastatic nodules in the pleura or pericardium, or malignant pleural effusion or pericardial effusion.

 M0: Classified as M0 if there is no distant metastasis.

 Mx: Classified as Mx if distant metastasis cannot be determined.

#### Phase 2: Information Extraction From Medical Reports

This stage aimed to accurately extract lung cancer TNM staging information from medical reports to assist subsequent assessments. Through white-box testing, we found that the model frequently omitted key staging-related information from medical reports. To address this issue, we refined our prompt by explicitly listing all required information for extraction (Textbox 2). This enhancement improved the model’s ability to identify and prioritize relevant details from diverse medical image reports.

Additionally, in this version of the prompt, we instructed the model to focus on key sections, such as the “diagnostic opinion” and “conclusion”, to concentrate the model’s attention on essential information.

Textbox 2. **Key information extraction.**

Information requirements:

Extract the following information from the imaging report along with the rationale for the judgment:

Tumor size and location:

 What is the maximum diameter of the tumor in cm?

 Does the tumor grow across lobes?

 In which lung lobe is the tumor located?

Cancer nodule situation:

 What is the location and size of the cancer nodule (in cm)?

 Is there a cancer nodule present in different lobes on the same side? Provide the rationale and reasoning for the judgment.

 Is there a solitary cancer nodule present in the same lung lobe? Provide the rationale and reasoning for the judgment.

#### Phase 3: Independently Handling T, N, and M Staging

In this critical phase, we designed prompts to enable the LLM to independently handle T, N, and M staging. We observed that the T, N, and M stages in the NSCLC TNM staging system corresponded to different judgment logic and medical knowledge. We also found that combining all staging criteria in a single prompt led to misinterpretation of different staging standards.

Therefore, we separated T, N, and M staging and developed specialized prompts for each category. This separation allowed the model to focus precisely on the unique criteria and nuances of each staging category, enhancing its accuracy and reliability.

Given the complexity of T staging, we particularly focused on refining its criteria. We broke down the matching conditions into prioritized steps, providing systematic guidance for T staging judgments (Textbox 3).

Textbox 3. **Guidance for primary tumor characteristics (T) staging judgments.**

# ”T Staging Criteria and Workflow”:

Step 1: Extract relevant information from the imaging report according to primary tumor assessment criteria.

Step 2: Assess for T4 stage. If any of the following conditions are met, classify as T4. If not, proceed to Step 3.

Condition 1: Tumor size 50‐70 mm with trans-lobar growth.

Condition 2: Invasion of any T4 anatomical structures.

Condition 3: Presence of cancer nodules in different ipsilateral lobes.

Step 3: Assess for T3 stage. If any of the following conditions are met, classify as T3. If not, proceed to Step 4.

Condition 1: Invasion of any T3 anatomical structures.

Condition 2: Presence of isolated cancer nodule in the same lobe.

Condition 3: Tumor size <50 mm with trans-lobar growth.

Step 4: Assess for T2 stage. If the following condition is met, classify as T2. If not, proceed to Step 5.

Condition 1: Presence of atelectasis or obstructive pneumonia extending to hilar region.

Step 5: If none of the above conditions are met but tumor size is available, classify based on the following criteria:

- ≤10 mm: T1a
- >10 mm to ≤20 mm: T1b
- >20 mm to ≤30 mm: T1c
- >30 mm to ≤40 mm: T2a
- >40 mm to ≤50 mm: T2b
- >50 mm to ≤70 mm: T3
- >70 mm: T4

Step 6: If no primary tumor is mentioned, classify as T0.

#### Phase 4: Optimizing for Detailed Clinical Standards

In the final phase, we optimized the prompts to align with more comprehensive clinical standards as mentioned in the Criteria for TNM and Clinical Staging subsection. We analyzed and categorized the causes of model misjudgments and compiled them as background knowledge. This knowledge was then injected into the model before judgment to decrease the error rate. For instance, we emphasized the detailed criteria of “Tumor Invasion,” “Cancer Nodule,” and “Lymph Node Metastasis” (supplementary rules of T0 in Table 1). These enhancements improved the model’s capacity to process complex medical narratives.

We also introduced supplementary judgment rules, such as clearly defining the invasion sites for different stages (supplementary rules of T2, T3, and T4 in Table 1), and formulated the corresponding prompt as follows (Textbox 4). These supplements helped reduce ambiguity and enhance staging consistency and accuracy.

Textbox 4. **Examples of supplementary judgment rules.**

T4 staging invasion sites:

Does the report clearly indicate that the tumor invades any of the following structures: diaphragm, mediastinum, heart, major vessels (aorta, superior vena cava, inferior vena cava, main pulmonary arteries, and left or right pulmonary veins within the pericardium), trachea, recurrent laryngeal nerve, esophagus, vertebral bodies, or carina? Provide the specific evidence and rationale for the judgment.

T3 staging invasion sites:

Does the report clearly indicate that the tumor invades the parietal pleura (usually noted in pathological staining), chest wall (including Pancoast tumors), phrenic nerve, or pericardium? Provide the specific evidence and rationale for the judgment.

Does the report clearly indicate that the tumor invades the main stems of more distal arteries or veins? Provide the specific evidence and rationale for the judgment.

T2 staging invasion sites:

Does the report clearly indicate that the tumor invades the left or right main bronchus or visceral pleura? Provide specific evidence and rationale for the judgment.

Through comprehensive and iterative white-box testing, the deficiencies of the LLM in NSCLC TNM staging were identified. During this process, we optimized the prompts to address these deficiencies, thereby guiding the staging process of the LLM to enhance its performance. This improvement addressed problems including incomplete information extraction, inadequate handling of TNM staging complexities, and limited comprehension of medical terminology.

Despite these improvements, the model still faced challenges in numerical calculations and prompt adherence. These issues affected the accuracy of staging results, particularly in complex T staging. These limitations were addressed in the subsequent supervised fine-tuning (SFT) phase.

### SFT

The SFT process adjusts the model parameters using supervised learning techniques. This allows the model to adapt to specific data distributions and task requirements, leading to improved performance. In this study, we used low-rank adaptation (LoRA) technology for SFT on the GLM-4-Air model. LoRA is a parameter-efficient fine-tuning technique. It reduces computational complexity and memory requirements by decomposing the weight update matrix while maintaining fine-tuning effectiveness [26].

Based on our annotated training dataset, we reprocessed the stage labels. To be specific, the stage label was reprocessed to the reasoning process consistent with the optimized prompt instructions by professional physicians (Textbox 5). This made the training data more comprehensible to the GLM-4-Air model, providing a solid foundation for model training.

Textbox 5. **Example of annotated training data for primary tumor characteristics (T) staging model.**

Original data:

Content: Medical Imaging Report Content

Label: Stage T2a

Re-processed data for supervised fine-tuning (SFT):

{

 “messages”: [

    {

      “role”: “system,”

      “content”: “You are a senior chief physician specializing in non-small cell lung cancer diagnosis.”

    },

    {

      “role”: “user,”

      “content”: “Prompt and Medical Imaging Report Content.”

    },

    {

      “role”: “assistant,”

      “content”: {

       “report_extraction_results”: {

        “tumor_size_and_location”: {

         “maximum_tumor_diameter(mm)“: “39,”

         “tumor_cross_lobe_growth”: “No,”

         “tumor_lobe_location”: “Left lower lobe”

        },

        “cancer_nodule_status”: {

         “nodule_location_and_size(mm)“: “None,”

         “ipsilateral_different_lobe_nodules”: “No,”

         “isolated_nodule_same_lobe”: “No”

        },

         “T4_stage_invasion_sites”: “None,”

         “T3_stage_invasion_sites”: “None”

      },

      “T_staging”: {

       “reasoning_process”: "Based on the imaging report, the tumor’s maximum diameter is 3.9 cm, located in the left lower lobe. No invasion of T4 stage structures is mentioned, and no cancer nodules are found in ipsilateral different lobes, which does not meet T4 criteria. The tumor diameter is less than 5 cm, with no invasion of T3 stage structures mentioned and no isolated nodules in the same lobe, which does not meet T3 criteria. The tumor diameter is greater than 3 cm but less than or equal to 4 cm, with no mention of the tumor causing atelectasis or obstructive pneumonia extending to the hilar region; therefore, it is staged as T2a.",

     “reasoning_conclusion”: “Stage T2a”

     }

    }

   }

 ]

}

To deal with the potential risks of overfitting and catastrophic forgetting associated with the limited sample size, we introduced a key design in the SFT stage for the core task. Specifically, we constructed a heterogeneous instruction-tuning dataset by blending the 292 target-domain cases with approximately 2000 general-purpose instruction samples covering diverse tasks such as mathematics, summarization, and translation. The details of the categories, exact counts, proportions, and specific examples for each category of the instruction samples are provided in Multimedia Appendix 3. Based on our empirical assessment, these components were mixed at a ratio of approximately 1:7. This strategy was designed to leverage a multi-task learning framework, compelling the model to adapt to the specific medical task while simultaneously preserving and reinforcing its general instruction-following and reasoning capabilities, thereby enhancing its generalization stability.

In the fine-tuning phase, we used the LoRA method for SFT with the following parameters (Table 2), and the GPU we used in this task was NVIDIA A800.

**Table 2.** Supervised fine-tuning parameter settings.

Parameter	Value
Rank	16
LoRa^a^ dropout	0.05
Learning rate	≈2.5×10^–4^-5×10^–4^
Batch size	8
Precision	FP16
Scheduler	Linear Decay
Random seeds	1102
Number of trainable parameters	about 4M
Stopping criteria	3 epoch

a LoRA: low-rank adaptation.

These parameters were carefully tuned to ensure efficient training and stable performance of the model.

During the SFT process, we used white-box testing to identify and analyze deficiencies in the model’s medical knowledge and capabilities. Based on these findings, we conducted targeted parameter optimization and fine-tuning to enhance its TNM staging performance. The results showed that this refined tuning strategy, based on continuous feedback and optimization, improved the model’s accuracy and stability in NSCLC TNM staging.

### Model Evaluation

#### Overview

Both models were evaluated on the held-out test sets (comprising both the White-Box and Black-Box sets). To ensure a comprehensive, unbiased, and statistically robust comparison of the performance between the GLM-4-Air and GPT-4o models, we used a standardized framework for evaluation. This framework encompasses performance metrics, statistical comparison methods, clinical impact assessment, and cost-effective evaluation, as detailed below.

It is important to clarify that all the evaluations were performed under the same prompts, test datasets, and evaluation environments to ensure fairness and validity. As a concrete specification of these standardized conditions, the key inference configuration parameters, including temperature, top_p, max_tokens, and max_attempts, used for both GLM-4-Air and GPT-4o are detailed in Table 3.

**Table 3.** Inference configuration table for both models.

Parameter	Value
temperature	0.01
top_p	0.01
max_tokens	8192
max_attempts	3

#### Performance Metrics

##### Accuracy

The overall proportion of correct predictions compared with clinical annotation made by a model for a given stage and clinical stage. The mapping from TNM staging to the overall clinical stage was strictly determined according to the AJCC 8th edition criteria (Figure 3). Note that a theoretically “correct” clinical stage group could be generated from an incorrect combination of individual T, N, or M predictions. To ensure transparency and to help validate clinical safety beyond simple accuracy percentages, we also calculate and report the “Exact Match Ratio,” which represents the percentage of cases where T, N, and M are all correctly predicted for the same patient.

##### Confidence Intervals

CIs were used to quantify the uncertainty of the evaluation results, reflecting the precision of the performance metric estimates. The 95% CIs calculated in this study indicate that we can be 95% confident that the model’s true performance on a larger, similarly distributed test set would fall within this range.

##### Confusion Matrices

The confusion matrices of both models in both white-box and black-box were calculated to show the specific misclassifications of TNM stagings.

##### Precision

Precision is defined as the proportion of true positive cases among all samples predicted as positive by the model. This metric evaluates the accuracy of the model’s positive predictions. For instance, in M1 staging, precision represents the proportion of cases correctly classified as M1 among all cases predicted by the model as M1.

##### Recall

Recall is defined as the proportion of true positive cases correctly identified by the model among all actual positive samples. This metric evaluates the completeness of the model in identifying relevant instances. For example, in M1 staging, recall represents the proportion of true M1 cases that are correctly identified by the model.

##### *F*_1_-score

The *F*_1_-score represents the harmonic mean of precision and recall, providing a single composite metric that balances both the model’s accuracy in positive predictions (precision) and its completeness in identifying relevant instances (recall). For the multi-class classification tasks (T, N, and M staging), we also report the macro-average *F*_1_-score, which treats all classes equally and offers a perspective unbiased by class distribution. The formula for calculating the *F*_1_-score is as follows,


$$F_{1}-score=2\times \frac{Precision\times Recall}{Precision+Recall}$$

### Statistical Comparison

To determine if the observed performance differences between models were statistically significant, we applied paired significance testing. Specifically, for the key metric of classification accuracy, we performed McNemar’s test to compare the paired categorical outcomes of our final model (GLM-4-Air) and the baseline model (GPT-4o).

To calculate a standardized measure of the strength of association between categorical variables, we used the statistic Cohen ω [27,28], defined by the following formula. This metric provides a standardized effect size that is independent of sample size, facilitating comparisons across studies.

The formula for Cohen ω is:


$$\omega =\sqrt{\frac{\chi^{2}}{n}}$$

where *χ*² is the chi-square test statistic, reflecting the overall deviation of the observed data from the expected distribution, and n is the total sample size.

According to Cohen's conventional benchmarks, the effect size can be interpreted as follows: *ω* of 0.1 indicates a small effect, *ω* of 0.3 a medium effect, and *ω* of 0.5 a large effect. This statistic provides an objective assessment of the practical importance of observed differences or associations, moving beyond reliance solely on the statistical significance of *P* values.

### Clinical Impact Assessment

In clinical practice, the consequences of a misclassification in TNM staging are not uniform; an error can range from being inconsequential to leading to significant deviations in treatment planning. Therefore, beyond conventional performance metrics, we conducted a clinical impact analysis to evaluate the practical implications of model errors.

Based on the confusion matrices, all misclassifications were systematically categorized into 3 tiers according to their potential impact on clinical decision-making as follows:

- Category I (Major Errors): These are the most severe errors where the TNM misclassification results in a significant change in the overall clinical stage, substantially altering both diagnostic evaluation and treatment strategy (eg, shifting from a curative to a palliative intent).
- Category II (Moderate Errors): These errors are of moderate severity. The TNM misclassification may sometimes lead to a change in clinical stage, potentially impacting the choice of subsequent therapy (eg, altering the recommended adjuvant treatment regimen), but without a fundamental shift in the treatment goal.
- Category III (Minor Errors): These are the least severe errors. The TNM misclassification has a minimal to negligible impact on the final clinical stage and the selection of treatment options, typically involving substage distinctions that do not change the standard of care.

All erroneous predictions made by the models on the test set were retrospectively reviewed, classified according to this 3-tiered system, and quantified. This analysis provides a crucial safety-centric perspective on model performance, assessing its reliability and potential for adoption in real-world clinical workflows.

In addition, a detailed breakdown of error examples and their categorization rationale is provided in Table 4.

**Table 4.** Taxonomy of primary tumor characteristics (T), regional lymph node involvement (N), and distant metastasis status (M) staging errors with exemplars and rationale.

Error category and specific error types	Clinical impact
I - Major errors	
Misclassification between M0 and M1	Misjudgment of tumor metastasis status, leading to incorrect clinical staging, can significantly impact the selection of treatment options.
Misclassification between T0 and Tn	Misinterpretation of nodules as tumors or tumors as benign nodules, as well as failure to detect tumors, can lead to incorrect clinical staging and may significantly impact the selection of treatment approaches.
Misclassification between T1/T4, or T2/T4	Discrepancies in staging (Stage I/III or II/III) observed with N0, or discrepancies in staging (Stage II/III) observed with N1, can lead to incorrect staging results, while tumors classified as N2 and N3 are uniformly staged as Stage III. However, all the aforementioned scenarios may influence the selection of treatment approaches.
Misclassification between N0/N2 or N0/N3	Discrepancies in staging (Stage I/III or II/III) observed with T1, T2, and T3 can lead to incorrect staging results, while tumors classified as T4 are uniformly staged as Stage III. However, all the aforementioned scenarios may influence the selection of the extent of lymph node dissection.
II - Moderate errors	
Misclassification between T1 and T2	Aside from the potential for staging discrepancies between Stage I and II when classified as N0, other scenarios, while not resulting in incorrect staging outcomes, may influence the selection of treatment approaches.
Misclassification between T1/T3 or T2/T3	Aside from the staging discrepancies (Stage I/II) observed with N0 or the staging discrepancies (Stage II/III) observed with N1, other factors will not lead to incorrect staging results but may influence the selection of treatment approaches.
Misclassification between T3 and T4	Aside from the staging discrepancies (Stage IIB/IIIA) observed with N0, other factors will not lead to incorrect staging results, but may influence the selection of radiotherapy and chemotherapy.
Misclassification between N0 and N1	Apart from the staging discrepancies (Stage I/II) observed with T1 and T2a, the staging discrepancies (Stage II/III) observed with T3, or the discrepancies within the same major stage (Stage IIA/IIB) observed with T2b, other factors (including T4) will not lead to incorrect staging results, but all the aforementioned scenarios may influence the selection of treatment approaches.
Misclassification between N1/N2 or N1/N3	Apart from the staging discrepancies (Stage IIB/IIIA) observed with T1 and T2, tumors classified as T3 and T4 are uniformly staged as Stage III. However, all the aforementioned scenarios may influence the selection of treatment approaches.
III - Minor errors	
Misclassification among M1 Subcategories (M1a, M1b, and M1c)	All cases were clinically staged as Stage IV, but the specific metastatic pattern may influence the selection of treatment approaches for extrathoracic metastases.
Misclassification among T1 Subcategories (T1a, T1b, and T1c)	Does not cause a staging error (in this case, clinical stage is determined by N or M). Typically, it does not affect treatment.
Misclassification between N2 and N3	In this case, all clinical stages are Stage III. It only minimally affects refined adjustments within the treatment strategy, rather than the fundamental selection of the treatment regimen.
Any misclassification involving Tx, Nx, or Mx categories	The clinical stage cannot be determined and requires further evaluation based on the disease presentation.

### Cost-Effective Evaluation

To assess the model’s deployment feasibility on accessible hardware, we performed a local performance benchmark using consumer-grade GPUs (Four NVIDIA GeForce RTX 4090, 24 GB VRAM each). The GLM-4-Air model was loaded at full precision with an 8K token context length, and its inference latency for each TNM staging component was measured on the black-box test set.

## Results

### OCR and Interannotator Agreement

The Chinese character error rates on the randomly selected 27 reports from white-box and black-box settings were 0.26% and 0.22%, respectively, which is consistent with the data provided by the supplier [22]. Manual review confirmed that no serious errors were found in the extraction of core information critical for TNM staging, such as tumor size, nodal status, or metastasis presence, in all cases. Specifically, the primary errors in this study were confined to issues like erroneous line breaks, spurious spaces between characters, omitted/misplaced punctuation, and poor paragraph segmentation, none of which impacted the model’s TNM staging decisions. The detailed results of the post-OCR Chinese character error rate test and examples of common OCR error types can be found in Multimedia Appendix 4.

The result of the calculation of interannotator agreement using the Cohen kappa coefficient among the clinical annotators is shown in Table 5. For the white-box set, Kappa values were 0.714 (T stage), 0.755 (N stage), and 0.778 (M stage). In the black-box set, agreement levels were 0.851 (T stage), 0.796 (N stage), and 0.796 (M stage). All Kappa values exceeded 0.70, indicating substantial agreement across all staging categories in both test sets. The T stage demonstrated the most pronounced improvement in interrater reliability between white-box and black-box evaluations, while N and M stages maintained consistently high agreement levels across both settings.

**Table 5.** Interannotator agreement based on Cohen's Kappa coefficient.

Data set	T^a^ kappa value (κ)	N^b^ kappa value (κ)	M^c^ kappa value (κ)
White-box	0.714	0.755	0.778
Black-box	0.851	0.796	0.796

a T: primary tumor characteristics.

b N: regional lymph node involvement.

c M: distant metastasis status.

### Prompt Optimization

**Figure 4.**
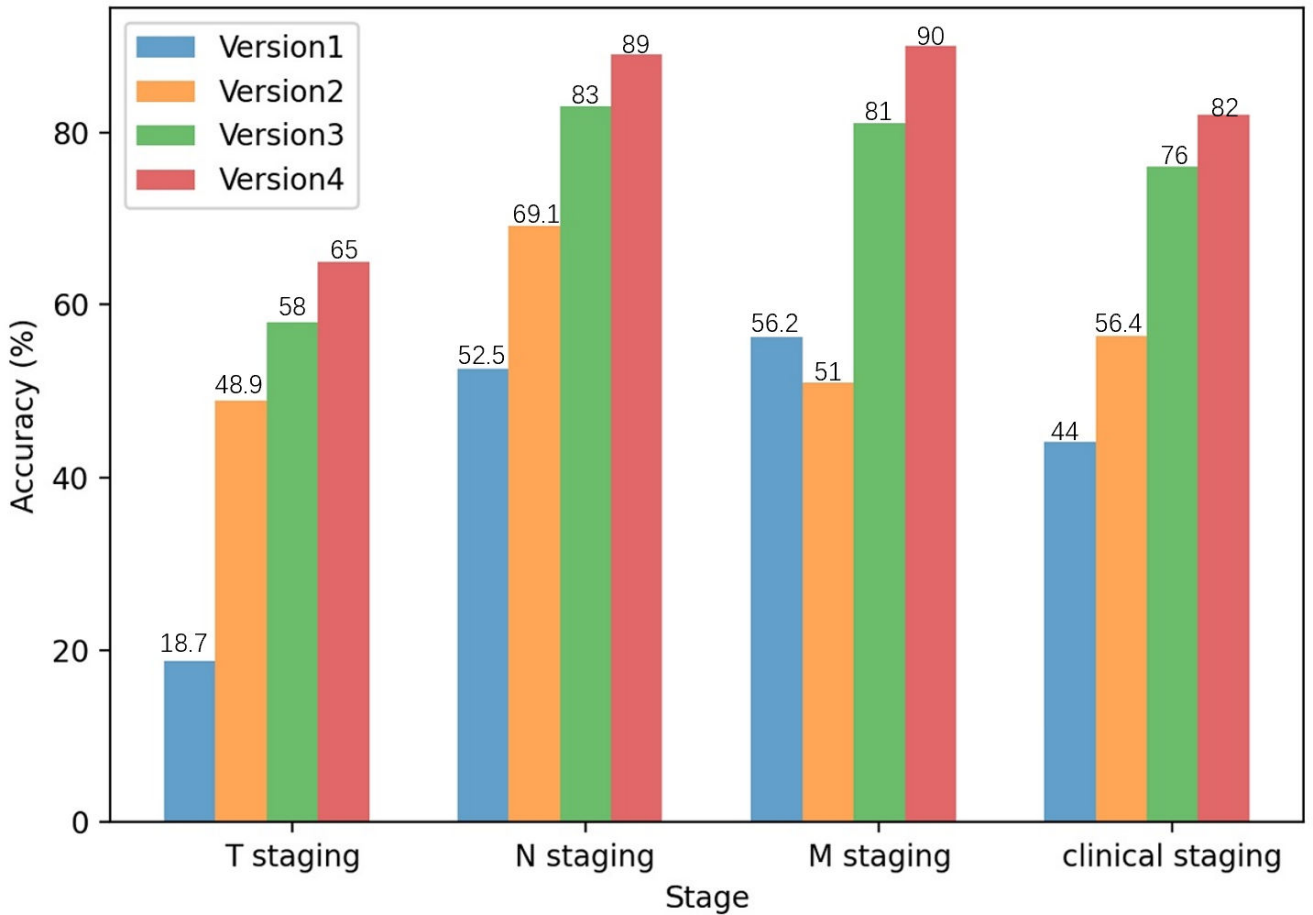
Comparison of accuracy across iterative prompt versions. M: distant metastasis status; N: regional lymph node involvement; T: primary tumor characteristics.

The results of this study demonstrate a general and substantial improvement in staging accuracy across successive prompt iterations. While an isolated decrease was observed in M staging from Version 1 to Version 2, all T and N stages, as well as the overall clinical stage, exhibited a consistent upward trend in accuracy from Version 1 through Version 4. Figure 4 and Table 6 compare the accuracy rates across 4 versions of prompts (0705 v, 0708 v, 0715 v, and 0801 v) for T, N, M, and clinical staging. The accuracy rates improved across all staging categories: T staging rose from 18.7% to 65%, N staging from 52.5% to 89%, and M staging from 56.2% to 90%. These improvements led to an increase in overall clinical staging accuracy from 44% to 82%.

**Table 6.** Accuracy of primary tumor characteristics (T), regional lymph node involvement (N), distant metastasis status (M), and clinical staging of iterative prompt versions.

	T staging (%)	N staging (%)	M staging (%)	Clinical staging (%)
Version 1	18.7	52.5	56.2	44
Version 2	48.9	69.1	51.0	56.4
Version 3	58	83	81	76
Version 4	65	89	90	82

The notable improvements occurred in 2 instances: first, between version 1 and version 2 for T staging, with accuracy increasing by 30.2% following the implementation of structured information extraction; second, between Version 2 and Version 3 for M staging, through the implementation of diverse prompts for independent T, N, and M staging. The data also showed that M and N staging maintained consistently higher accuracy than T staging across all versions.

### SFT

**Figure 5.**
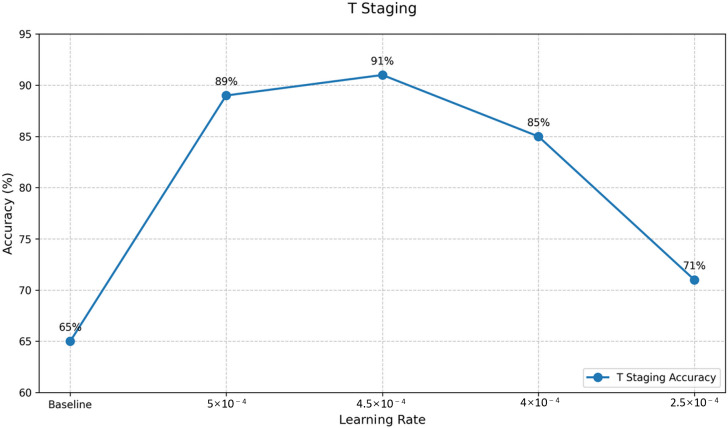
Supervised fine-tuning effects corresponding to learning rates: T staging accuracy. T: primary tumor characteristics.

**Figure 6.**
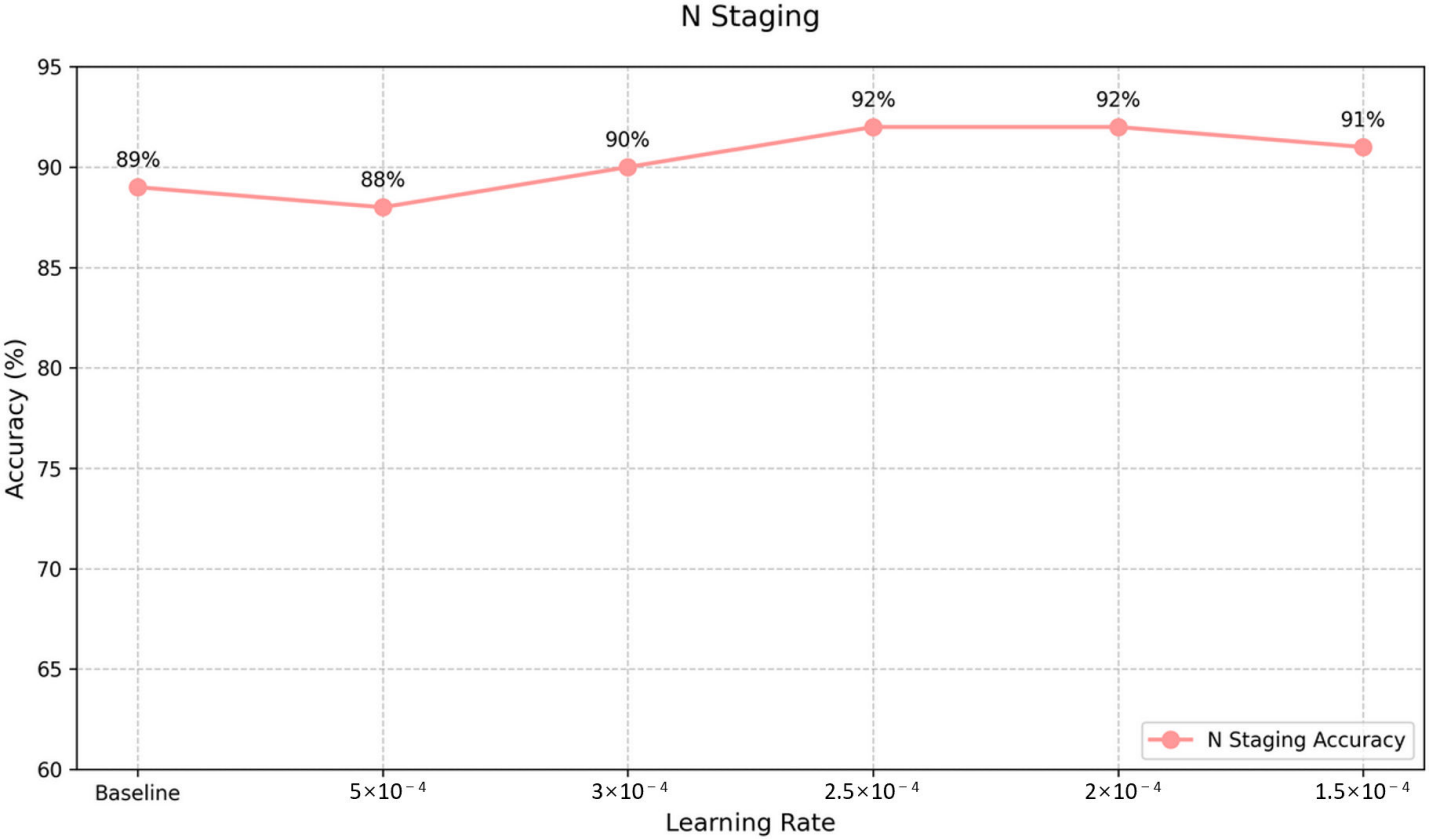
Supervised fine-tuning effects corresponding to learning rates: N staging accuracy. N: regional lymph node involvement.

**Figure 7.**
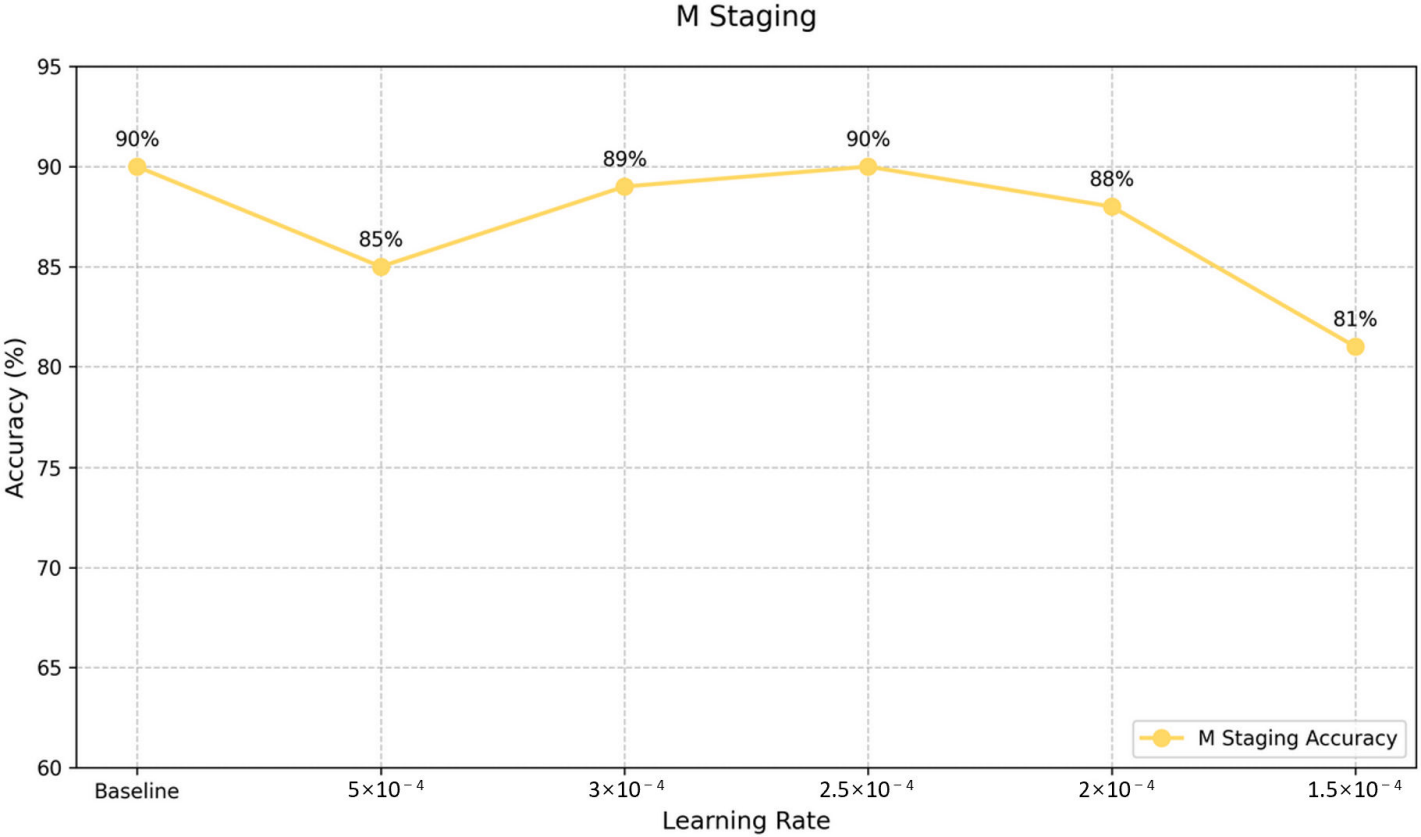
Supervised fine-tuning effects corresponding to learning rates: M staging accuracy. M: distant metastasis status.

Analysis of the LoRA fine-tuning results (Figures 5-7) led to the selection of optimal models for T and N staging, with learning rates of 4.5e-4 and 2.5e-4, respectively. For M staging, as no fine-tuned models exceeded the original model’s performance, the baseline GLM-4-Air model was retained, maintaining its accuracy of 90%.

The results demonstrated the effectiveness of the training process, particularly for T and N staging tasks. The accuracy of T staging improved from 65% to 91%, representing a substantial increase of 26%.

### Model Evaluation

#### Performance Metrics

In the black-box test, the GLM-4-Air model with SFT demonstrated superior performance compared with the GPT-4o model across all categories. Specifically, the GLM-4-Air with SFT achieved accuracy scores of 92% for T staging, 86% for N staging, 92% for M staging, and 90% for clinical staging. As a comparison, the GPT-4o recorded accuracy scores of 87%, 70%, 78%, and 80% for T, N, M, and clinical staging, respectively (Table 7). This performance aligned with the white-box test results. The GLM-4-Air model with SFT consistently showed higher accuracy across all stages compared with GPT-4o. It should be noted that the Clinical Staging accuracy is a derivative metric, calculated based on the combined T, N, and M outputs according to the mapping table (Figure 3), rather than a direct, independent prediction of the model.

**Table 7.** Performance comparison of different models in non-small cell lung cancer tumor node metastasis staging across White-box and Black-box tests. GLM-4-Air (general language model) (original): The base GLM-4-Air model without supervised fine-tuning; GLM-4-Air (supervised fine-tuning [SFT]): The GLM-4-Air model optimized through our training framework; GPT-4o: A large language model released on May 13, 2024.

Data set and model	Accuracy(%)				
T^a^ staging	N^b^ staging	M^c^ staging	Exact match	Clinical staging
White-box
GLM-4-Air (Original)	65	89	90	54	80
GLM-4-Air (SFT)	91	92	90	80	93
GPT-4o	86	80	70	61	77
Black-box
GLM-4-Air (SFT)	92	86	92	77	90
GPT-4o	87	70	78	64	80

a T: primary tumor characteristics.

b N: regional lymph node involvement.

c M: distant metastasis status.

**Figure 8.**
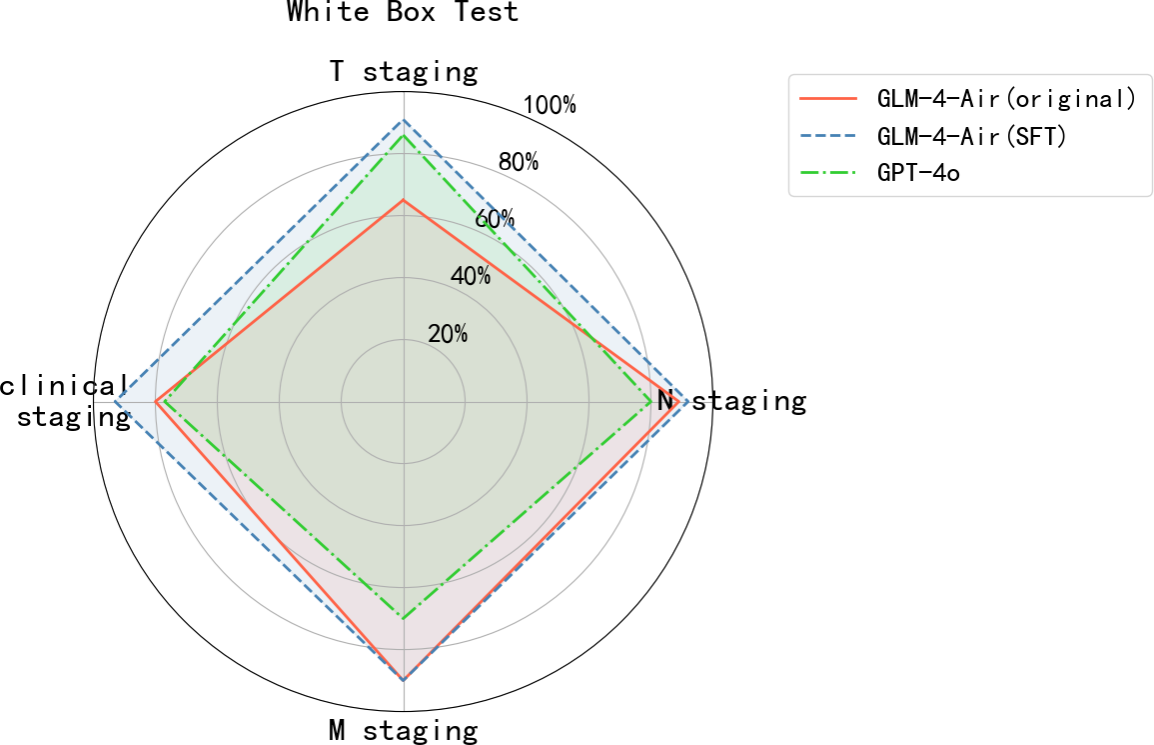
The accuracy of GLM-4-Air (original and SFT) and GPT-4o in the white-box test. GLM: general language model; M: distant metastasis status; N: regional lymph node involvement; SFT: supervised fine-tuning; T: primary tumor characteristics.

As shown in Figures8  9, the GLM-4-Air (SFT) demonstrated superior performance in white-box testing across all staging categories compared with both its original version and GPT-4o. This was maintained in black-box testing, with GLM-4-Air (SFT) consistently outperforming GPT-4o. The robust performance validated the effectiveness of the fine-tuning approach in enhancing the model’s staging capabilities.

**Figure 9.**
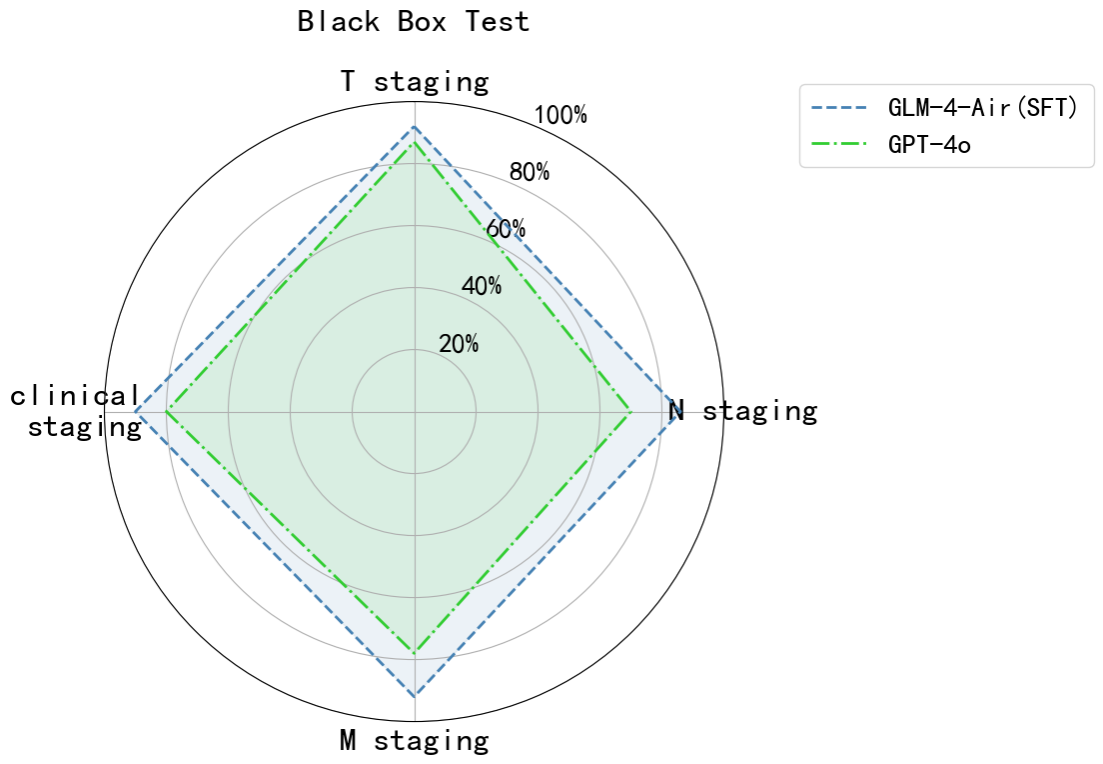
The accuracy of GLM-4-Air (SFT) and GPT-4o in the black-box test. GLM: general language model; M: distant metastasis status; N: regional lymph node involvement; SFT: supervised fine-tuning; T: primary tumor characteristics.

The calculated 95% CIs are presented in Table 8 below.

**Table 8.** Comparison of accuracy (95% CI) for tumor node metastasis staging of GLM-4-Air (general language model) and GPT-4o.

Model and date set	Stage	Accuracy (%)	Accuracy (95% CI)
GLM-4-Air 32B (original)			
White-box			
	T^a^	65	0.552‐0.736
	N^b^	89	0.814‐0.938
	M^c^	90	0.826‐0.945
GLM-4-Air 32B (SFT)^d^			
White-box			
	T	91	0.838‐0.952
	N	92	0.850‐0.959
	M	90	0.826‐0.945
Black-box			
	T	92	0.850‐0.959
	N	86	0.779‐0.915
	M	92	0.850‐0.959
GPT-4o			
White-box			
	T	86	0.779‐0.915
	N	80	0.711‐0.867
	M	70	0.604‐0.781
Black-box			
	T	87	0.790‐0.922
	N	70	0.604‐0.781
	M	78	0.689‐0.850

a T: primary tumor characteristics.

b N: regional lymph node involvement.

c M: distant metastasis status.

d SFT: supervised fine-tuning.

When comparing GLM-4-Air before and after SFT on the white-box test set, the CI for T staging significantly narrowed, improving from 0.552‐0.736 to 0.838‐0.952. A similar narrowing was observed for N staging, with the interval improving from 0.814‐0.938 to 0.850‐0.959. The CI for M staging remained unchanged due to the model not being fine-tuned for this specific category.

In the white-box test comparison between GLM-4-Air and GPT-4o, the CIs for GLM-4-Air were superior to those of GPT-4o across all individual TNM categories. This pattern was consistent in the black-box test comparison, where GLM-4-Air again demonstrated higher CIs than GPT-4o for each TNM category.

**Figure 10.**
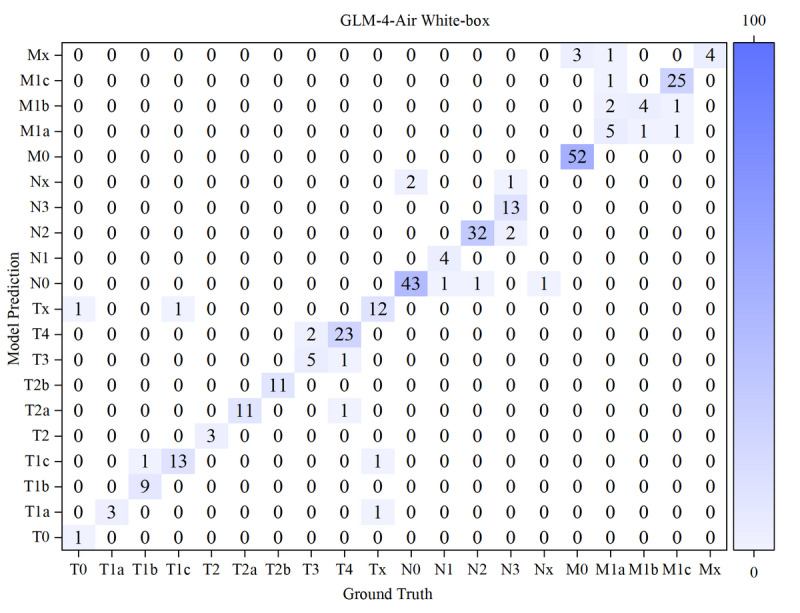
Confusion matrix for T, N, and M staging by GLM-4-Air in white-box. GLM: general language model; M: distant metastasis status; N: regional lymph node involvement; T: primary tumor characteristics.

**Figure 11.**
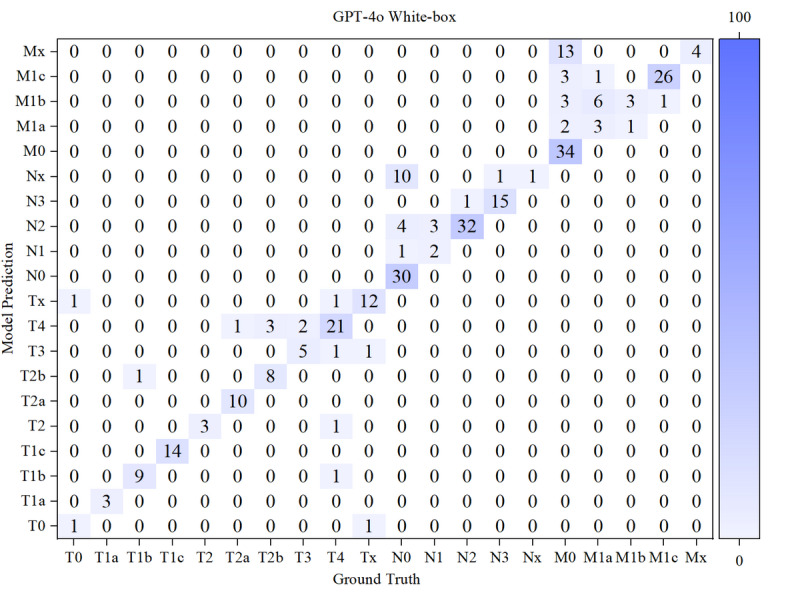
Confusion matrix for T, N, and M staging by GPT-4o in white-box. M: distant metastasis status; N: regional lymph node involvement; T: primary tumor characteristics.

**Figure 12.**
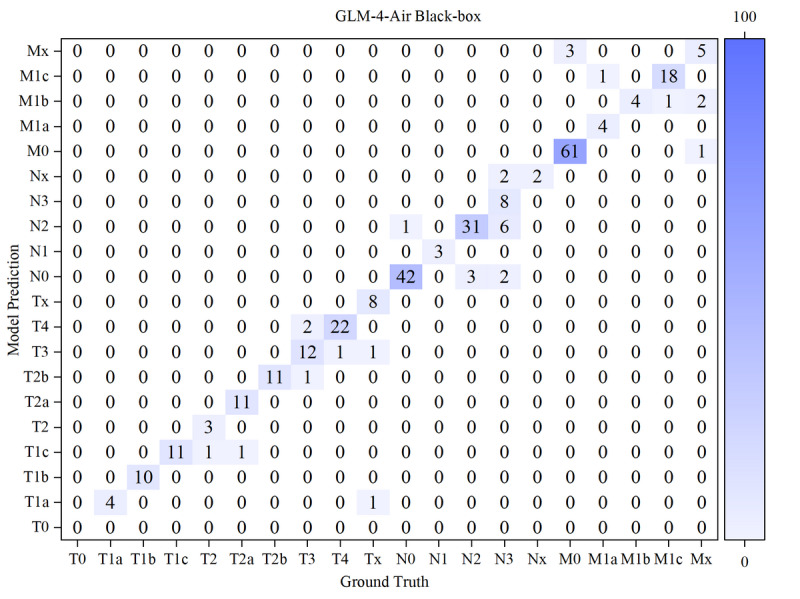
Confusion matrix for T, N, and M staging by GLM-4-Air in a black box. GLM: general language model; M: distant metastasis status; N: regional lymph node involvement; T: primary tumor characteristics.

**Figure 13.**
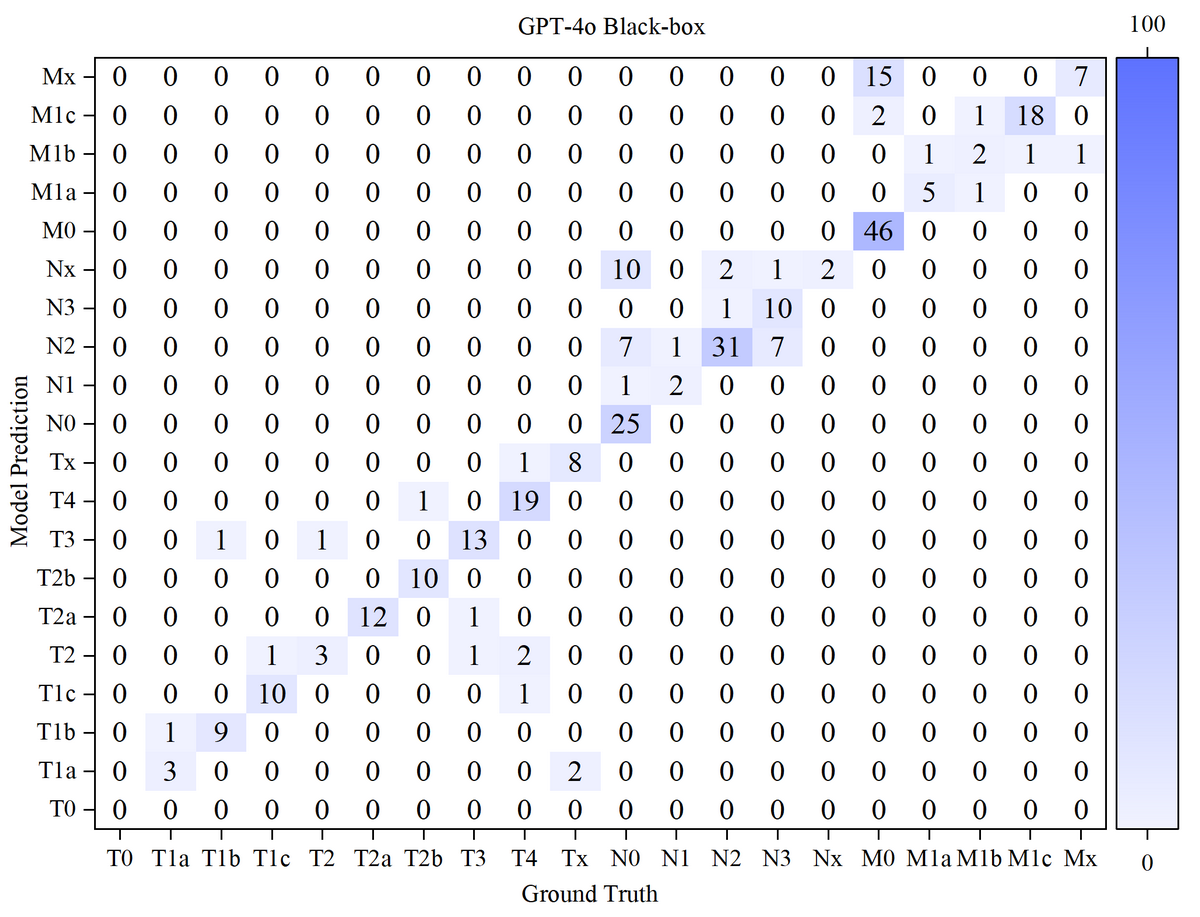
Confusion matrix for T, N, and M staging by GPT-4o in a black box. M: distant metastasis status; N: regional lymph node involvement; T: primary tumor characteristics.

The results of the confusion matrices of both models in white-box and black-box were shown in Figures 10-13 below.

**Figure 14.**
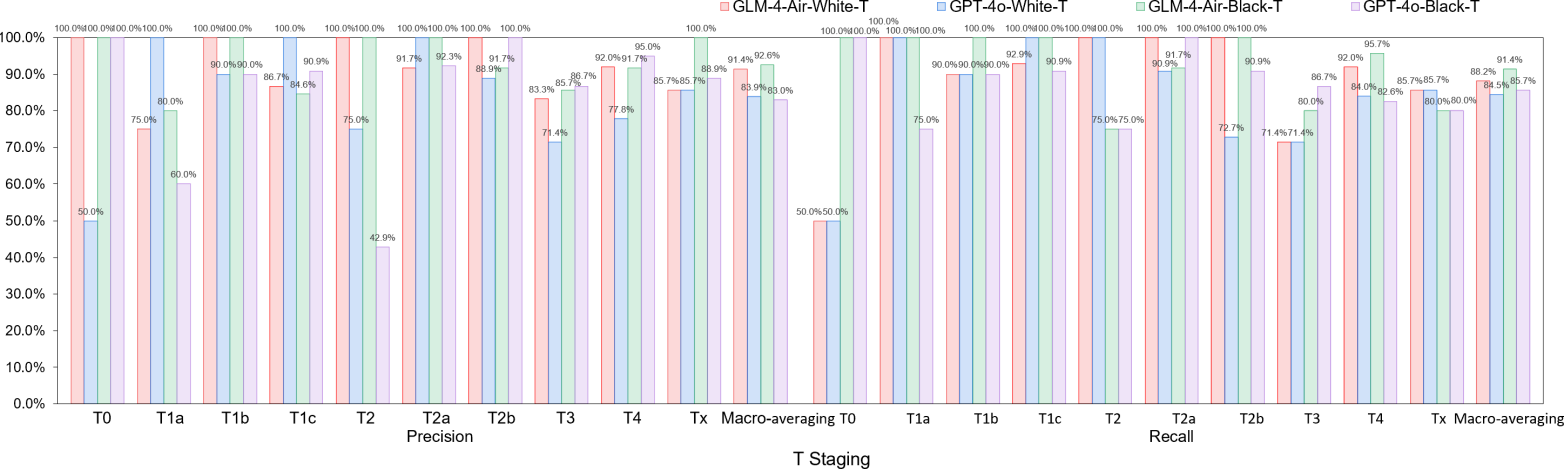
Comparison of precision and recall for T staging between GLM-4-Air and GPT-4o in the white-box and black-box tests. GLM: general language model; T: primary tumor characteristics.

**Figure 15.**
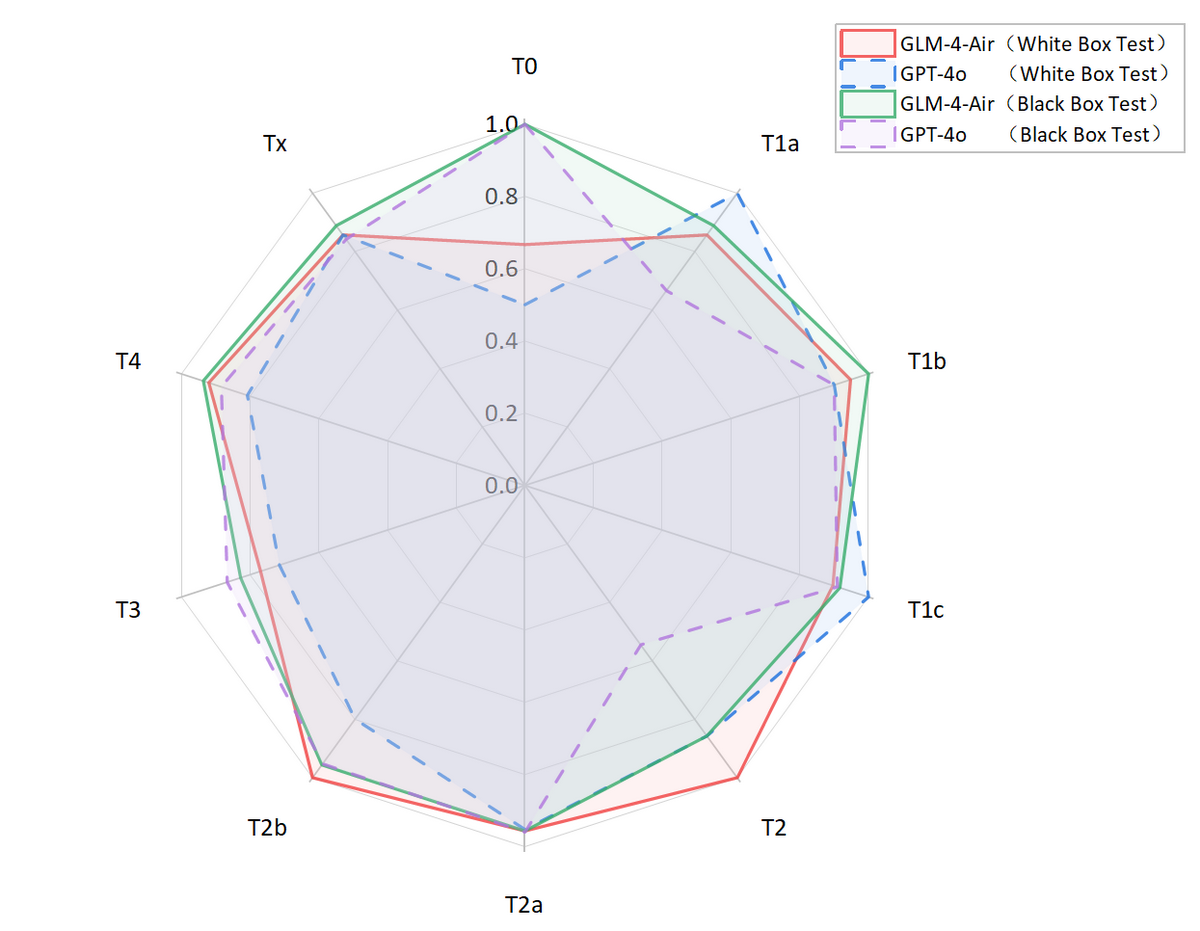
The *F*_1_-score of T staging of GLM-4-Air and GPT-4o in the white-box and black-box tests. GLM: general language model; T: primary tumor characteristics.

**Figure 16.**
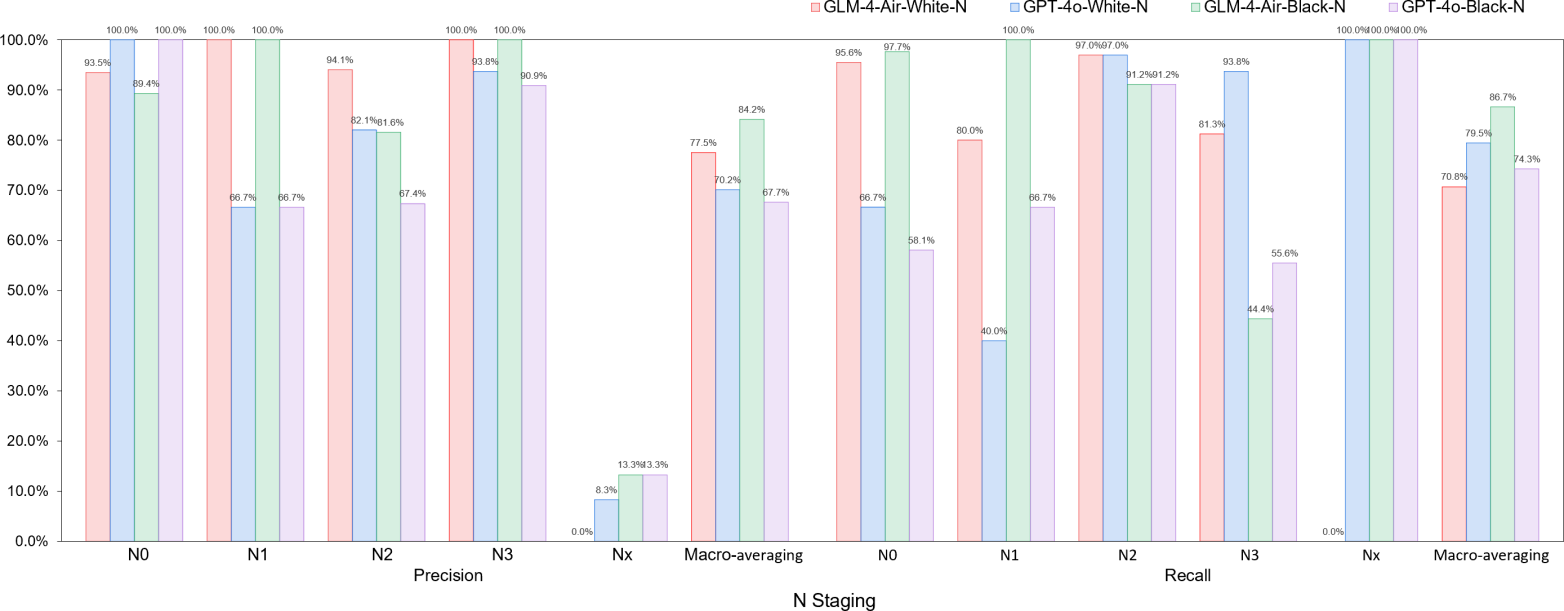
Comparison of precision and recall for N staging between GLM-4-Air and GPT-4o in the white-box and black-box tests. GLM: general language model; N: regional lymph node involvement.

**Figure 17.**
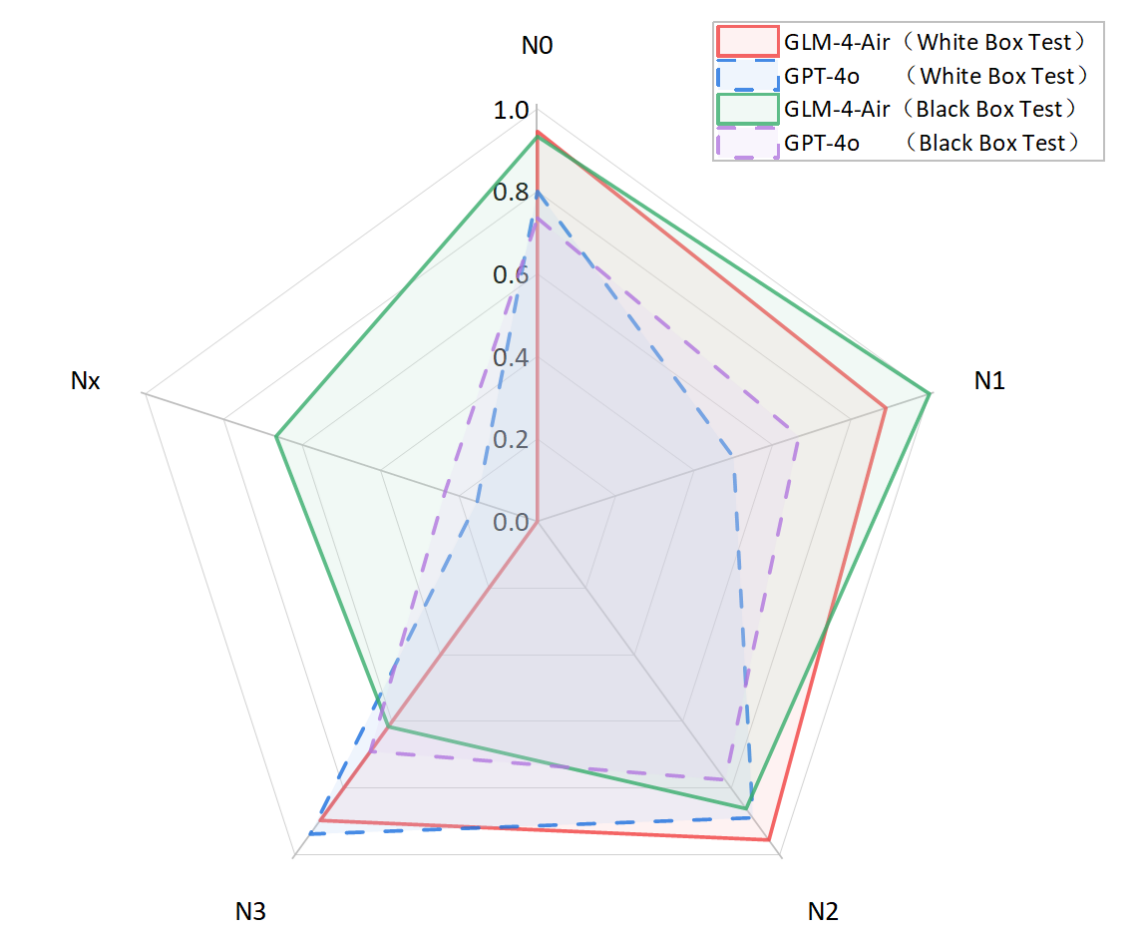
The *F*_1_-score of N staging of GLM-4-Air and GPT-4o in the white-box and black-box tests. GLM: general language model; N: regional lymph node involvement.

**Figure 18.**
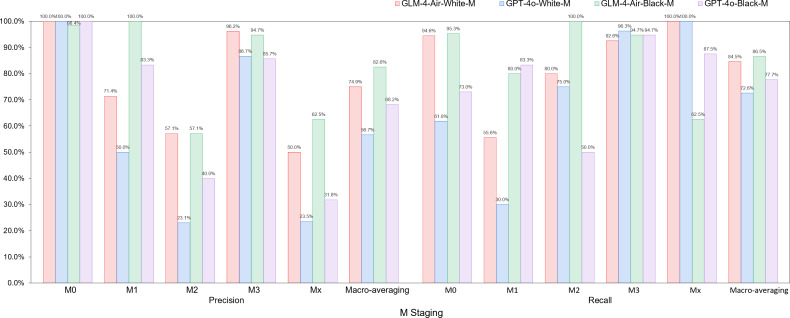
Comparison of precision and recall for M staging between GLM-4-Air and GPT-4o in the white-box and black-box tests. GLM: general language model; M: distant metastasis status.

**Figure 19.**
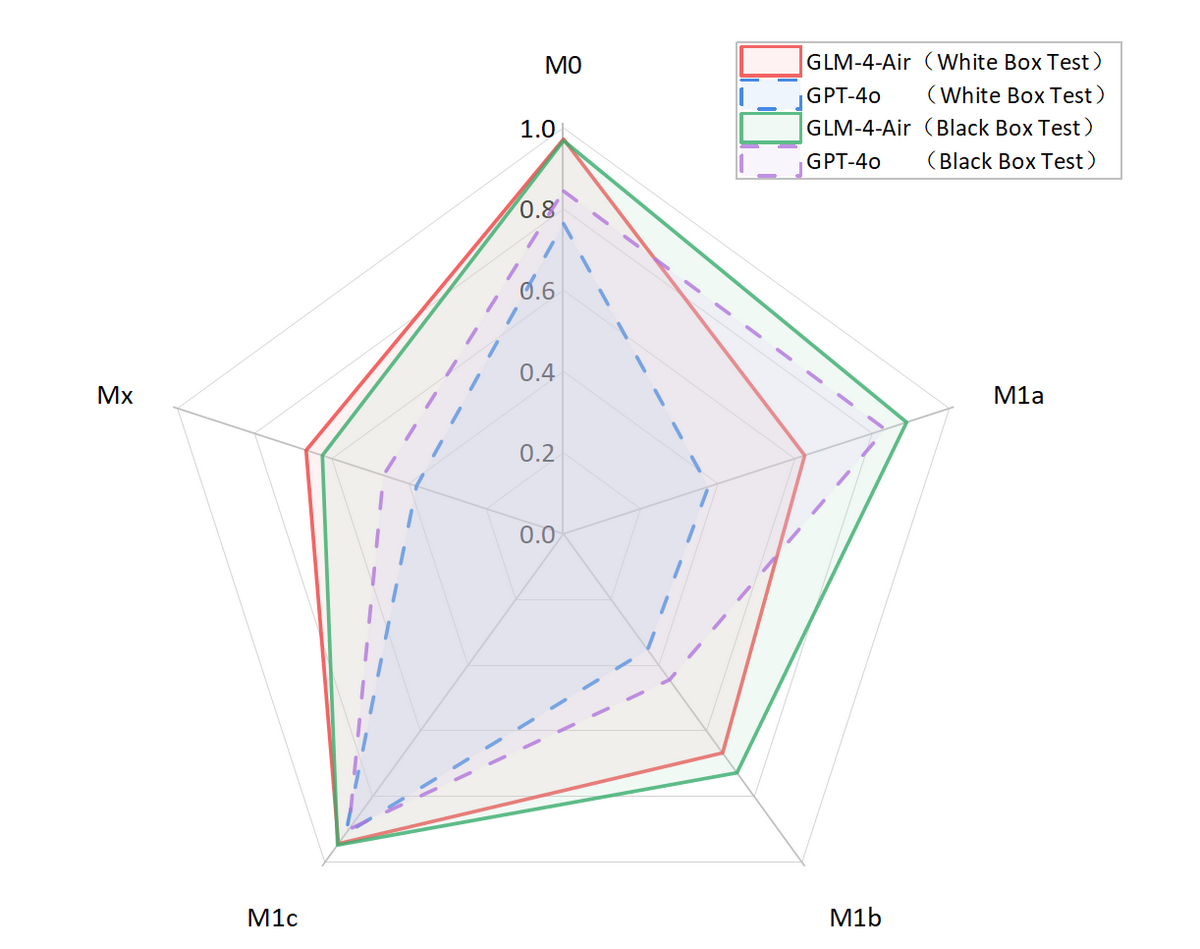
The *F*_1_-score of M staging of GLM-4-Air and GPT-4o in the white-box and black-box tests. GLM: general language model; M: distant metastasis status.

Based on the confusion matrices, we calculated the precision, recall, and *F*_1_-score for all subcategories of the T, N, and M stages for both models in the white-box and black-box test sets, as shown in Tables 9-11 and Figures 14-19.

**Table 9.** Comparison of precision, recall, and *F*_1_-score for primary tumor characteristics (T) staging between GLM-4-Air (general language model) and GPT-4o.

Model and T staging	Precision (%)	Recall (%)	*F*_1_-score
GLM-4-Air (white-box)
T0	100.0	50.0	0.667
T1a	75.0	100.0	0.857
T1b	100.0	90.0	0.947
T1c	86.7	92.9	0.897
T2	100.0	100.0	1.000
T2a	91.7	100.0	0.956
T2b	100.0	100.0	1.000
T3	83.3	71.4	0.769
T4	92.0	92.0	0.920
Tx	85.7	85.7	0.857
Macro-averaging	91.4	88.2	0.887
GPT-4o (white-box)
T0	50.0	50.0	0.500
T1a	100.0	100.0	1.000
T1b	90.0	90.0	0.900
T1c	100.0	100.0	1.000
T2	75.0	100.0	0.857
T2a	100.0	90.9	0.952
T2b	88.9	72.7	0.800
T3	71.4	71.4	0.714
T4	77.8	84.0	0.808
TX	85.7	85.7	0.857
Macro-averaging	83.9	84.5	0.839
GLM-4-Air (black-box)
T0	100.0	100.0	1.000
T1a	80.0	100.0	0.889
T1b	100.0	100.0	1.000
T1c	84.6	100.0	0.917
T2	100.0	75.0	0.857
T2a	100.0	91.7	0.957
T2b	91.7	100.0	0.956
T3	85.7	80.0	0.828
T4	91.7	95.6	0.936
Tx	100.0	80.0	0.889
Macro-averaging	92.6	91.4	0.914
GPT-4o (black-box)
T0	100.0	100.0	1.000
T1a	60.0	75.0	0.667
T1b	90.0	90.0	0.900
T1c	90.9	90.9	0.909
T2	42.9	75.0	0.546
T2a	92.3	100.0	0.960
T2b	100.0	90.9	0.952
T3	86.7	86.7	0.867
T4	95.0	82.6	0.884
Tx	88.9	80.0	0.842
Macro-averaging	83.0	85.7	0.836

**Table 10.** Comparison of precision, recall, and *F*_1_-score for regional lymph node involvement (N) staging between GLM-4-Air (general language model) and GPT-4o.

Model and N staging	Precision (%)	Recall (%)	*F*_1_-score
GLM-4-Air (white-box)
N0	93.5	95.6	0.945
N1	100.0	80.0	0.889
N2	94.1	97.0	0.955
N3	100.0	81.2	0.897
Nx	0.0	0.0	0.000
Macro-averaging	77.5	70.8	0.737
GPT-4o (white-box)
N0	100.0	66.7	0.800
N1	66.7	40.0	0.500
N2	82.0	97.0	0.889
N3	93.8	93.8	0.938
Nx	8.3	100.0	0.154
Macro-averaging	70.2	79.5	0.656
GLM-4-Air (black-box)
N0	89.4	97.7	0.933
N1	100.0	100.0	1.000
N2	81.6	91.2	0.861
N3	100.0	44.4	0.615
Nx	50.0	100.0	0.667
Macro-averaging	84.2	86.7	0.815
GPT-4o (black-box)
N0	100.0	58.1	0.735
N1	66.7	66.7	0.667
N2	67.4	91.2	0.775
N3	90.9	55.6	0.690
Nx	13.3	100.0	0.235
Macro-averaging	67.7	74.3	0.620

**Table 11.** Comparison of precision, recall, and *F*_1_-score for distant metastasis status (M) staging between GLM-4-Air (general language model) and GPT-4o.

Model and M staging	Precision (%)	Recall (%)	*F*_1_-score
GLM-4-Air (white-box)			
M0	100.0	94.6	0.972
M1a	71.4	55.6	0.625
M1b	57.1	80.0	0.667
M1c	96.2	92.6	0.943
Mx	50.0	100.0	0.667
Macro-averaging	74.9	84.5	0.775
GPT-4o (white-box)			
M0	100.0	61.8	0.764
M1a	50.0	30.0	0.375
M1b	23.1	75.0	0.353
M1c	86.7	96.3	0.912
Mx	23.5	100.0	0.381
Macro-averaging	56.7	72.6	0.557
GLM-4-Air (black-box)			
M0	98.4	95.3	0.968
M1a	100.0	80.0	0.889
M1b	57.1	100.0	0.727
M1c	94.7	94.7	0.947
Mx	62.5	62.5	0.625
Macro-averaging	82.6	86.5	0.831
GPT-4o (black-box)			
M0	100.0	73.0	0.844
M1a	83.3	83.3	0.833
M1b	40.0	50.0	0.444
M1c	85.7	94.7	0.900
Mx	31.8	87.5	0.467
Macro-averaging	68.2	77.7	0.698

The results for each T, N, and M stage will be elaborated separately in this section, with corresponding discussions provided in the Discussion section.

- White-box Evaluation (T): In the T1a subcategory, GLM-4-Air demonstrated precision and recall of 75.0% and 100.0%, respectively, showing slightly lower precision but equivalent recall compared with GPT-4o (100.0% and 100.0%). For T1b staging, GLM-4-Air achieved a precision of 100.0% and a recall of 90.0%, outperforming GPT-4o (90.0% for both metrics) in precision. In the T1c subcategory, GLM-4-Air demonstrated precision and recall of 86.7% and 92.9%, respectively, underperforming GPT-4o (both 100.0%). In T2 staging, GLM-4-Air attained 100.0% of both precision and recall versus GPT-4o (75.0% and 100.0%). For T2b, GLM-4-Air maintained performance of 100.0% for both precision and recall, compared with GPT-4o (88.9 % precision and 72.7% recall). In T3 staging, GLM-4-Air showed precision of 83.3% and recall of 71.4%, surpassing GPT-4o in precision (71.4%) while matching recall. For T4 staging, GLM-4-Air achieved 92.0% for both metrics, exceeding GPT-4o (77.8% precision and 84.0% recall).
- Black-box Evaluation (T): In T1a staging, GLM-4-Air attained 80.0% precision and 100.0% recall, outperforming GPT-4o (60.0% and 75.0%). For T1b, GLM-4-Air achieved 100.0% of both precision and recall compared with GPT-4o’s both 90.0%. In T1c staging, GLM-4-Air showed 84.6% precision and 100.0% recall, while GPT-4o achieved 90.9% for both metrics. For T2a, GLM-4-Air demonstrated 100.0% precision and 91.7% recall versus GPT-4o’s 92.3% and 100.0%, with each model excelling in different metrics. In T3 staging, GLM-4-Air showed 85.7% precision and 80.0% recall, slightly lower than GPT-4o (86.7% both). For T4 staging, GLM-4-Air achieved 91.7% precision and 95.6% recall compared with GPT-4o’s 95.0% and 82.6%.
- *F*_1_-score Analysis (T): In white-box testing, GLM-4-Air showed slightly lower *F*_1_-scores in T1a and T1c (0.857 and 0.897) compared with GPT-4o (1.000 in both). Both models achieved identical *F*_1_-scores (0.857) in Tx staging. However, GLM-4-Air demonstrated higher *F*_1_-scores in all other T subcategories. In black-box testing, GLM-4-Air showed marginally lower *F*_1_-scores in T2a and T3 (0.956 and 0.828) compared with GPT-4o (0.960 and 0.867), but outperformed GPT-4o in all remaining subcategories. Notably, GLM-4-Air demonstrated more balanced and stable performance across all T subcategories. In black-box testing, GLM-4-Air maintained *F*_1_-scores above 0.828 across all T subcategories, while GPT-4o showed greater variability, particularly in T2 staging, where its *F*_1_-score dropped to 0.546.
- Macro-average Metrics (T): In white-box testing, GLM-4-Air achieved macro-average precision, recall, and *F*_1_-score of 91.4%, 88.2%, and 0.887, respectively, compared with GPT-4o’s 83.9%, 84.5%, and 0.839. In black-box testing, GLM-4-Air demonstrated further improvement with macro-average metrics of 92.6%, 91.4%, and 0.914, consistently exceeding GPT-4o’s corresponding values of 83.0%, 85.7%, and 0.836.
- White-box Evaluation (N): In the N0 subcategory, GLM-4-Air demonstrated a slightly lower precision than GPT-4o (93.48% vs 100.0%) but achieved a substantially higher recall (95.6% vs 66.7%). For N1 staging, GLM-4-Air attained a precision of 100.0% and a recall of 80.0%, outperforming GPT-4o (66.7% precision and 40.0% recall). In N2 staging, GLM-4-Air showed higher precision (94.1% vs 82.0%) while matching GPT-4o’s recall (97.0% for both models). For N3 staging, GLM-4-Air achieved precision of 100.0%, surpassing GPT-4o (93.8%).
- Black-box Evaluation (N): In the N0 subcategory, GLM-4-Air again showed lower precision than GPT-4o (89.4% vs 100.0%) but demonstrated significantly higher recall (97.7% vs 58.1%). For N1 staging, GLM-4-Air achieved scores of 100.0% for both precision and recall, while GPT-4o achieved 66.7% for both metrics. In N2 staging, GLM-4-Air showed higher precision (81.6% vs 67.4%) with both models achieving identical recall (91.2%). For N3 staging, GLM-4-Air attained precision of 100.0% compared with 90.9% of GPT-4o, though with a slightly lower recall (44.4% vs 55.6%).
- *F*_1_-score Analysis (N): In white-box testing, GLM-4-Air’s *F*_1_-score for the Nx subcategory was 0 due to it misclassifying the only Nx case in white-box; excluding this, GLM-4-Air achieved higher *F*_1_-scores than GPT-4o in the N0, N1, and N2 subcategories. However, in the N3 subcategory, GLM-4-Air’s *F*_1_-score (0.897) was slightly lower than GPT-4o’s (0.938). In black-box testing, GLM-4-Air demonstrated higher *F*_1_-scores than GPT-4o in the N0, N1, N2, and Nx subcategories. Although its *F*_1_-score in the N3 subcategory (0.615) was lower than GPT-4o’s (0.690), GLM-4-Air maintained *F*_1_-scores above 0.615 across all N subcategories, while GPT-4o’s minimum *F*_1_-score was 0.235 (in Nx subcategory).
- Macro-average Metrics (N): In white-box testing, GLM-4-Air achieved macro-average precision, recall, and *F*_1_-score of 77.5%, 70.8%, and 0.737, respectively, compared with GPT-4o’s 70.2%, 79.5%, and 0.656. In black-box testing, GLM-4-Air’s macro-average metrics were 84.2%, 86.7%, and 0.815, consistently outperforming GPT-4o’s corresponding values of 67.7%, 74.3%, and 0.620.
- White-box evaluation (M): In the white-box test set, the two models demonstrated identical precision (100.0%) only in the M0 subcategory. Across all other subcategories (M1a, M1b, M1c, Mx), GLM-4-Air consistently achieved higher precision than GPT-4o. Regarding recall, both models performed equally (100.0%) in the Mx subcategory. GLM-4-Air exhibited superior recall in the M0, M1a, and M1b subcategories compared with GPT-4o, though it showed a slightly lower recall in M1c (92.6% vs 96.3%).
- Black-box Evaluation (M): In the black-box test set, GLM-4-Air showed slightly lower precision than GPT-4o in the M0 subcategory (98.4% vs 100.0%). However, it outperformed GPT-4o in all remaining subcategories (M1a, M1b, M1c, Mx) in terms of precision. For recall, each model showed advantages in different areas: GLM-4-Air achieved higher recall in M0 and M1b, with a recall of 100.0% in M1b, which significantly surpassed GPT-4o (50.0%). Conversely, GLM-4-Air’s recall was marginally lower in M1a and Mx, while both models achieved identical recall in M1c.
- *F*_1_-score Analysis (M): In white-box testing, GLM-4-Air achieved higher *F*_1_-scores than GPT-4o across all M subcategories (M0, M1a, M1b, M1c, Mx). This pattern was consistent in black-box testing, where GLM-4-Air again demonstrated superior *F*_1_-scores in each subcategory. Additionally, GLM-4-Air maintained *F*_1_-scores above 0.625 across all M subcategories in both white-box and black-box settings, whereas GPT-4o’s lowest *F*_1_-scores were observed in M1b (0.353 in white-box and 0.444 in black-box).
- Macro-average Metrics (M): In white-box testing, GLM-4-Air achieved macro-average precision, recall, and *F*_1_-score of 74.9%, 84.5%, and 0.775, respectively, comprehensively outperforming GPT-4o (56.7%, 72.6%, and 0.557). Similarly, in black-box testing, GLM-4-Air’s macro-average metrics (82.6%, 86.5%, and 0.831) were significantly higher than those of GPT-4o (68.2%, 77.7%, and 0.698).

To evaluate the improvements GLM-4-Air achieved via SFT, we also compared the *F*_1_-scores for each staging subcategory before and after SFT in the white-box test set. Since the baseline model was ultimately selected for M staging, the comparison focused specifically on the T and N subcategories. The results are presented in Table 12 below. Combining with the analysis of the post-SFT white-box results for T and N staging (Tables9  10), it can be seen that GLM-4-Air demonstrated marked overall improvement in both T and N staging tasks following SFT.

**Figure 20.**
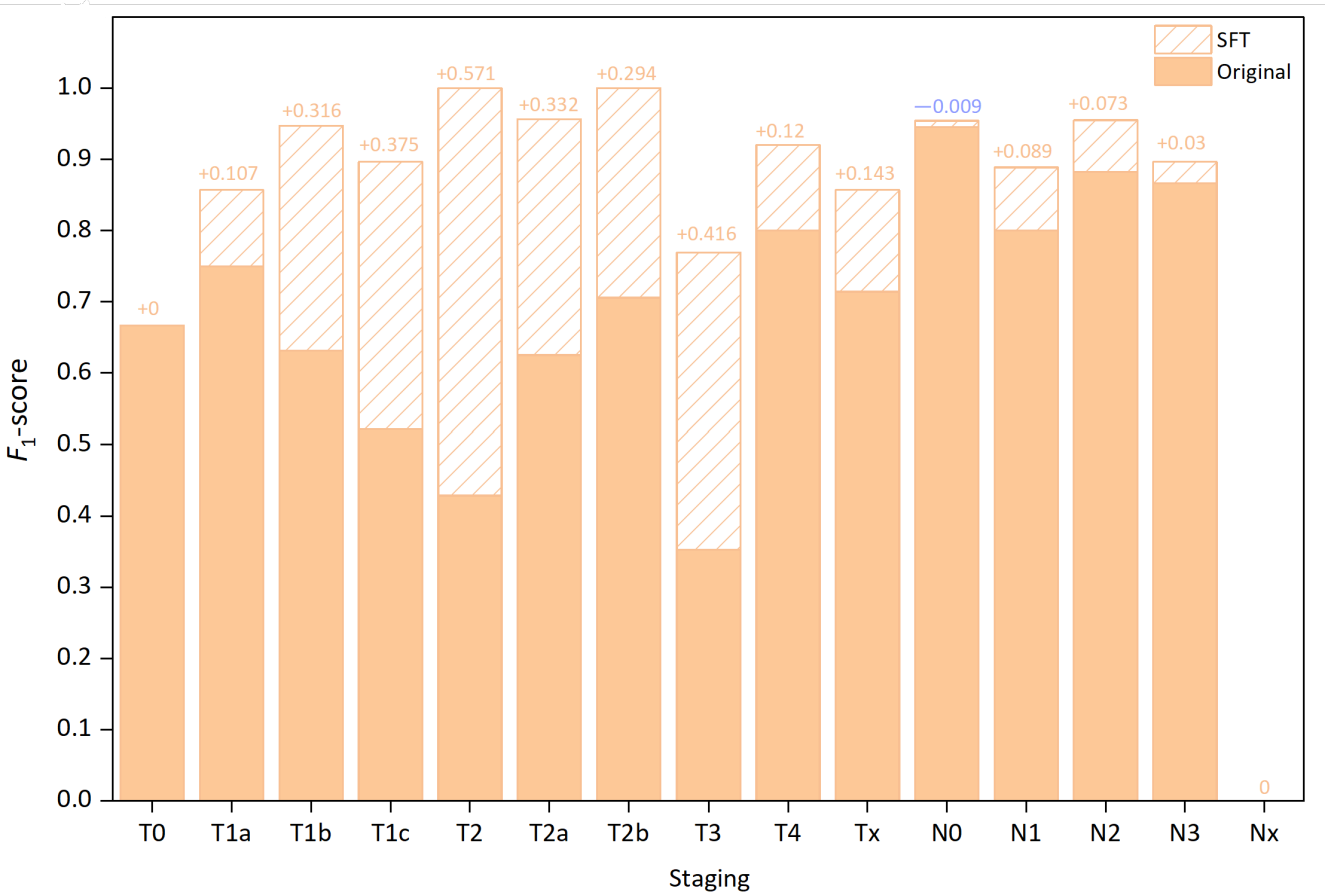
Changes in the *F*_1_-score for T and N staging of GLM-4-Air in the white-box test pre-SFT and post-SFT. GLM: general language model; N: regional lymph node involvement; SFT: supervised fine-tuning; T: primary tumor characteristics.

In addition, to provide a more intuitive demonstration of the performance improvement in GLM-4-Air after SFT, we calculated the difference in precision, recall, and *F*_1_-scores for each T and N staging subcategory following SFT. The difference in the *F*_1_-score of T and N staging was illustrated in Figure 20.

**Table 12.** Precision, recall, and *F*_1_-score for primary tumor characteristics (T) and regional lymph node involvement (N) staging of original (presupervised fine-tuning) GLM-4-Air (general language model).

Model and staging	Precision (%)	Recall (%)	*F*_1_-score
GLM-4-Air (Original)
T0	100.0	50.0	0.667
T1a	60.0	100.0	0.750
T1b	66.7	60.0	0.632
T1c	66.7	42.9	0.522
T2	27.3	100.0	0.429
T2a	100.0	45.4	0.625
T2b	100.0	54.6	0.706
T3	30.0	42.9	0.353
T4	73.3	88.0	0.800
Tx	71.4	71.4	0.714
Macro-Averaging	69.5	65.5	0.620
GLM-4-Air (Original)
N0	97.7	93.3	0.954
N1	80.0	80.0	0.800
N2	85.7	90.9	0.882
N3	92.9	81.2	0.867
Nx	0.0	0.0	0.000
Macro-Averaging	71.2	69.1	0.701
N1	100.0	80.0	0.889
N2	94.1	97.0	0.955
N3	100.0	81.2	0.897
Nx	0.0	0.0	0.000
Macro-Averaging	77.5	70.8	0.737

#### Comparison of T Staging Performance Before and After SFT

For the T0 subcategory, precision, recall, and *F*_1_-score remained unchanged. In T1a staging, precision improved from 60.0% to 75.0%, while recall remained at 100.0%, resulting in an increase in *F*_1_-score from 0.750 to 0.857. T1b staging showed substantial improvement: precision increased from 66.7% to 100.0%, recall from 60.0% to 90.0%, and *F*_1_-score from 0.632 to 0.947. The notable improvement was observed in T1c staging: precision increased from 66.7% to 86.7%, recall from 42.9% to 92.9%, and *F*_1_-score from 0.522 to 0.897. In T2 staging, precision improved markedly from 27.3% to 100.0%, and *F*_1_-score increased from 0.429 to 1.000. For T2a staging, precision slightly decreased from 100.0% to 91.7%, but recall substantially increased from 45.4% to 100.0%, raising the *F*_1_-score from 0.625 to 0.956. T2b staging maintained precision of 100.0% while recall increased from below 60.0% to 100.0%, improving the *F*_1_-score from 0.706 to 1.000. In T3 staging, precision, recall, and *F*_1_-score improved from 30.0%, 42.9%, and 0.353 to 83.3%, 71.4%, and 0.769, respectively. For T4 staging, precision increased from 73.3% to 92.0%, recall from 88.0% to 92.0%, and *F*_1_-score from 0.800 to 0.920. In Tx staging, precision, recall, and *F*_1_-score all improved from 71.4%, 71.4%, and 0.714 to 85.7%, 85.7%, and 0.857, respectively.

Macro-average metrics demonstrated substantial improvement after SFT. Specifically, precision increased from 69.5% to 91.4%, recall from 65.5% to 88.2%, and *F*_1_-score from 0.620 to 0.887.

#### Comparison of N Staging Performance Before and After SFT

For N0 staging, precision slightly decreased from 97.7% to 93.5% after SFT, but recall improved from 93.3% to 95.6%, maintaining a high *F*_1_-score of 0.945 (compared with 0.954 pre-SFT). The improvement was observed in N1 staging: precision increased from 80.0% to 100.0%, recall remained at 80.0%, and *F*_1_-score improved from 0.800 to 0.889. In N2 staging, precision increased from 85.7% to 94.1%, recall from 90.9% to 97.0%, and *F*_1_-score from 0.882 to 0.955. For N3 staging, precision improved from 92.9% to 100.0%, recall remained at 81.2%, and *F*_1_-score increased from 0.867 to 0.897. Macro-average metrics showed improvement after SFT: precision increased from 71.2% to 77.5%, recall from 69.1% to 70.8%, and *F*_1_-score from 0.701 to 0.737.

#### Statistical Comparison

The results of the paired significance tests (McNemar’s test) for model comparisons are summarized in Table 13 below.

**Table 13.** Results of the McNemar’s test for model comparisons between GLM-4-Air (general language model) and GPT-4o.

Parameters	White-box T^a^	Black-box T	White-box N^b^	Black-box N	White-box M^c^	Black-box M
Chi-square test (*df*)	14 (45)	14.3 (36)	23 (10)	24.5 (10)	21 (10)	15.9 (10)
*P* value	>.99	>.99	.01	.006	.02	.10
Significance	Not significant	Not significant	Significant	Significant	Significant	Not significant
Dataset (n)	100	100	100	100	100	100
Staging	T0, T1a, T1b, T1c, T2, T2a, T2b, T3, T4, Tx	T1a, T1b, T1c, T2, T2a, T2b, T3, T4, Tx	N0, N1, N2, N3, Nx	N0, N1, N2, N3, Nx	M0, M1a, M1b, M1c, Mx	M0, M1a, M1b, M1c, Mx
ω	0.374	0.378	0.480	0.495	0.458	0.399

a T: primary tumor characteristics.

b N: regional lymph node involvement.

c M: distant metastasis status.

For T staging, the differences were not statistically significant in both the black-box (*χ*²_36_=14.333, *P*=.99) and white-box (*χ*²_45_=14.000, *P*=.99) test sets.

In contrast, for N staging, statistically significant differences were observed in both the black-box (*χ*²_10_=24.533, *P*=.006) and white-box (*χ*²_10_=23.000, *P*=.01) test sets.

For M staging, the results differed between the test sets: a statistically significant difference was found in the white-box set (*χ*²_10_=20.974, *P*=.02), but not in the black-box set (*χ*²_10_=15.941, *P*=.10). It is worth noting that although the black-box model for M staging did not reach the statistical significance level, its chi-square test effect size reached *ω*=0.399, which is considered a medium-to-large effect.

#### Clinical Impact Assessment

**Figure 21.**
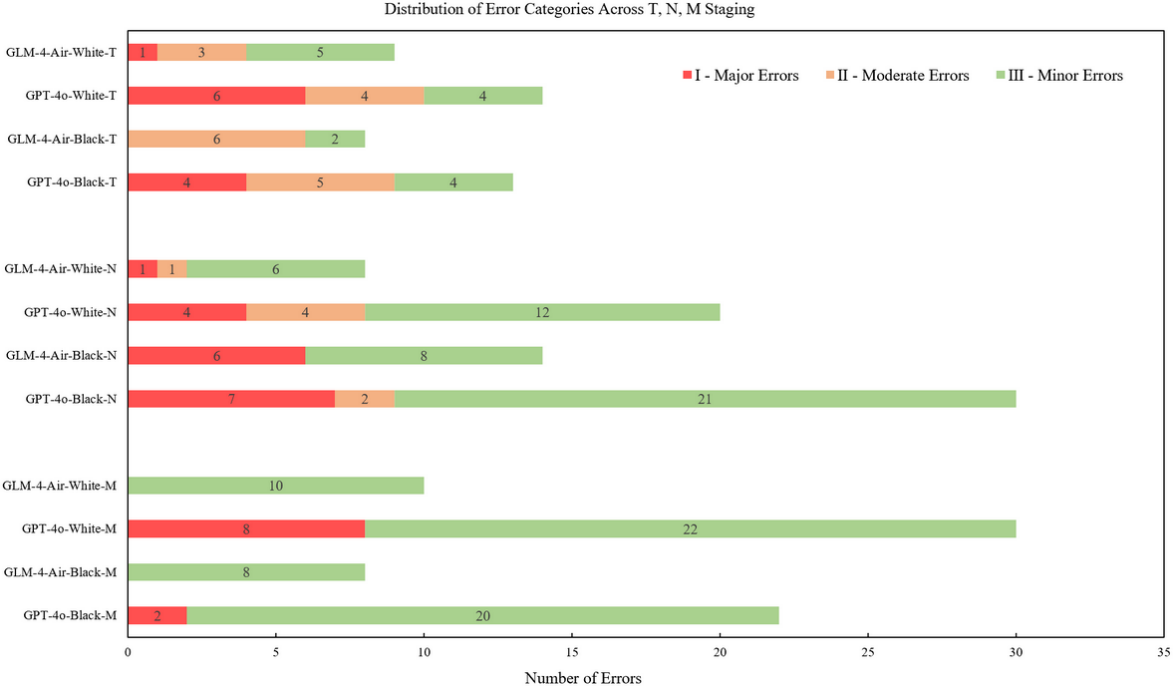
Distribution of error categories across T, N, and M staging on white-box and black-box of GLM-4-Air and GPT-4o. GLM: general language model; M: distant metastasis status; N: regional lymph node involvement; T: primary tumor characteristics.

Based on the previously defined error typology (Table 4), we systematically categorized all errors committed by both models in the white-box and black-box test sets. The detailed statistical results are presented in Table 14 and visualized in Figure 21.

**Table 14.** Distribution of error categories across primary tumor characteristics (T), regional lymph node involvement (N), and distant metastasis status (M) staging on White-box and Black-box of GLM-4-Air (general language model) and GPT-4o.

Model/Data Set/Stage	I-Major errors	II-Moderate errors	III-Minor errors	Total
GLM-4-Air-White-T	1	3	5	9
GPT-4o-White-T	6	4	4	14
GLM-4-Air-Black-T	0	6	2	8
GPT-4o-Black-T	4	5	4	13
GLM-4-Air-White-N	1	1	6	8
GPT-4o-White-N	4	4	12	20
GLM-4-Air-Black-N	6	0	8	14
GPT-4o-Black-N	7	2	21	30
GLM-4-Air-White-M	0	0	10	10
GPT-4o-White-M	8	0	22	30
GLM-4-Air-Black-M	0	0	8	8
GPT-4o-Black-M	2	0	20	22

In the white-box evaluation, GLM-4-Air demonstrated superior performance with fewer total errors compared with GPT-4o across all staging categories. Specifically, for T staging, GLM-4-Air committed 9 errors versus GPT-4o’s 14; for N staging, 8 errors versus 20; and for M staging, 10 errors versus 30.

This advantage remained consistent in the black-box evaluation. GLM-4-Air recorded 8, 14, and 8 errors in T, N, and M staging, respectively, while GPT-4o committed 13, 30, and 22 errors in the same categories.

Regarding error type classification, GLM-4-Air showed particularly notable performance in controlling category I errors. In T staging, GLM-4-Air committed only 1 category I error in white-box testing compared with GPT-4o’s 6, and did not commit such errors at all in black-box testing versus GPT-4o’s 4. For N staging, GLM-4-Air recorded 1 category I error in white-box (vs 4 for GPT-4o) and 6 in black-box (vs 7 for GPT-4o). In M staging, GLM-4-Air achieved 0 category I errors in both test sets, while GPT-4o committed 8 and 2 in white-box and black-box, respectively.

GLM-4-Air also demonstrated excellent performance in controlling category II errors. In T staging, it committed 3 category II errors in white-box (vs 4 for GPT-4o) and 6 in black-box (vs 5 for GPT-4o). For N staging, GLM-4-Air committed only 1 category II error in white-box (vs 4 for GPT-4o) and did not commit a category II error in black-box testing (vs 2 for GPT-4o). Neither model committed category II errors in M staging.

In category III errors, GLM-4-Air maintained an overall advantage. For T staging, it committed 5 category III errors in white-box (vs 4 for GPT-4o) and 2 in black-box (vs 4 for GPT-4o). In N staging, GLM-4-Air committed 6 category III errors in white-box (vs 12 for GPT-4o) and 8 in black-box (vs 21 for GPT-4o). For M staging, GLM-4-Air committed 10 category III errors in white-box (vs 22 for GPT-4o) and 8 in black-box (vs 20 for GPT-4o).

In addition, the evaluation results of strict and lenient interpretation strategies demonstrate that the risk of increasing false negatives under the strict strategy remains low. In the white-box test set, no false negative cases occurred, with the model correctly identifying all metastatic cases. In the black-box test set, only 1 false negative was observed, where an Mx case was misclassified as M0. Consequently, the false negative rate under the strict interpretation strategy was 0.5% (1/200), as visible in the confusion matrix in Figures10  12.

**Figure 22.**
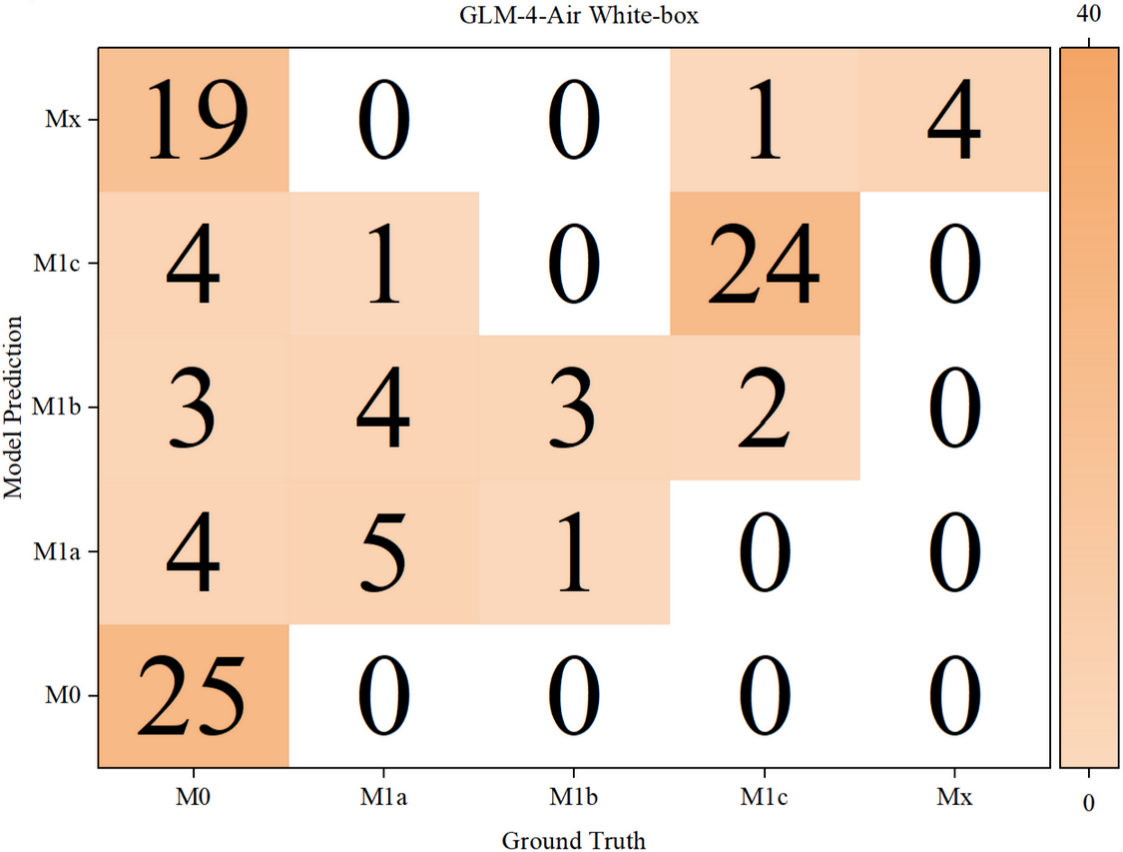
Confusion matrix for M staging using lenient strategy by GLM-4-Air in White-box test. GLM: general language model; M: distant metastasis status.

On the other hand, the lenient strategy significantly increased false positives, with cases incorrectly classified as M1 or MX instead of M0. The results, detailed in Figures22  23, show false positive rates of 30% (30/100) in the white-box set and 23% (23/100) in the black-box set.

**Figure 23.**
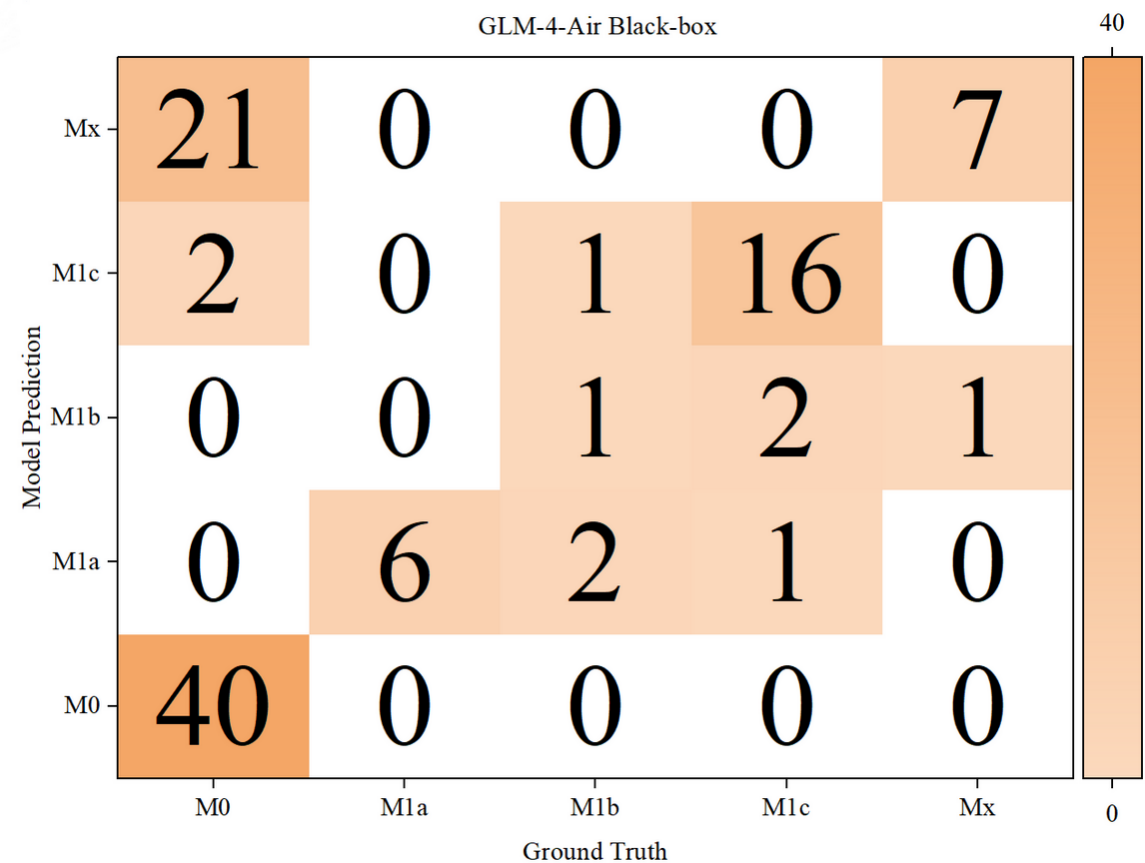
Confusion matrix for M staging using lenient strategy by GLM-4-Air in Black-box test. GLM: general language model; M: distant metastasis status.

#### Cost-Effective Evaluation

The local hardware benchmark confirmed the operational efficiency of the model on accessible hardware (Four NVIDIA GeForce RTX 4090, 24 GB VRAM each). The median inference latencies per case for the GLM-4-Air model were 8.510 seconds for T staging, 5.125 seconds for N staging, and 1.300 seconds for M staging on the black-box dataset.

## Discussion

### Principal Findings

The principal finding of this study is that a mid-scale LLM, when optimized through a systematic framework of clinical prompt engineering and SFT, can be developed into a highly effective and more practical tool in this specific clinical task, demonstrating the efficacy and potential of domain-specific adaptation. Our optimized GLM-4-Air model achieved higher accuracy in NSCLC TNM staging relative to the general-purpose GPT-4o, alongside a notable reduction in clinically critical errors and practical deployability on consumer-grade hardware with latencies acceptable for clinical workflows. These performance outcomes, particularly evident in the semantically complex N and M staging tasks, suggest that our hybrid optimization strategy can effectively enhance domain-specific reasoning and guideline adherence required for clinical interpretation. Furthermore, these results provide evidence that a specialized, moderately sized model can achieve high accuracy in complex clinical tasks, offering a complementary pathway to the scale-driven paradigm and highlighting the critical role of domain-specific optimization.

### OCR and Annotation

One of the keys to our research was the collection of high-quality, real-world data. We therefore invested considerable effort in data preparation, working closely with medical experts for rigorous validation and cleaning, which provides high-quality source material for the OCR process. We believe OCR is a mature technology now, and this study relies fundamentally on the LLM’s capacity for deep semantic understanding and information extraction from medical report text, not on exact string matching in a traditional way. Pretrained on massive text corpora, the LLM possesses inherent and robust capabilities for tolerating and correcting common OCR-induced noise by leveraging contextual information.

Our analysis also revealed that data quality, especially annotation quality, significantly impacts model performance, with even a small proportion of mislabeled samples potentially undermining the overall results. Given our limited dataset size, maintaining annotation quality became crucial. To ensure annotation quality and consistency, we developed standardized protocols aligned with AJCC TNM staging guidelines and implemented a multi-expert review mechanism. Annotators were instructed to make decisions strictly based on the medical reports’ textual content, minimizing subjective bias.

From the results of calculating and reporting the interannotator agreement using the Cohen kappa coefficient in Table 5, we observed an enhancement in T-stage agreement within the black-box set. This improvement may be attributed to the annotation process for the white-box set itself, possibly acting as a calibration phase. Through challenging cases, the annotators likely developed a more consistent internal standard for interpreting ambiguous T-stage descriptors (eg, invasion vs abutment). This refined, shared understanding was then applied during the black-box annotation, leading to higher immediate agreement. The sustained high agreement for N and M stages across both conditions reflects the more binary nature of nodal and metastatic assessment compared with the finer gradations required for T-stage determination. These findings underscore the context-dependent nature of staging reliability and highlight the value of multi-environment validation in assessing clinical annotation consistency.

### Prompt Optimization

Building upon the high-quality dataset, we realized three key strategies in prompt optimization were important to enhance the model’s performance: (1) structured output and CoT design, (2) independent modular architecture design, and (3) background knowledge injection.

First, structured output and CoT design were adopted to improve the readability and interpretability of the model’s output. In each staging task, the model not only provided the final conclusion but also presented the logical reasoning path. For example, in T staging, the model might state, “The tumor diameter exceeds 7 cm and invades the chest wall structures, thus meeting the criteria for T4 staging.” This approach ensures transparency, facilitating manual verification and error adjustment. Collaborating with medical professionals for white-box testing allowed us to analyze prediction errors and optimize prompts effectively.

Second, we implemented an independent modular architecture. We treated T (tumor size and location), N (lymph node metastasis), and M (distant metastasis) staging as separate tasks to reduce interference between staging judgments. To be specific, earlier versions had a 35% error rate in N staging due to mixed criteria. Modularization allowed the model to concentrate on specific tasks, increasing accuracy by approximately 30% and reducing cross-interference.

For example, in a case with the description “T9 vertebra suspected metastatic bone tumor,” the model initially misclassified it as M1a. After prompt optimization, it correctly identified it as T4 staging. This improvement was achieved through a specialized T staging prompt that detailed specific invasion criteria. The prompt listed T4 invasion sites, including diaphragm, mediastinum, heart, major vessels, trachea, recurrent laryngeal nerve, esophagus, vertebral bodies, and carina. This guided the model to correctly extract “T4 Invasion Sites”: “vertebrae.”

The model’s reasoning demonstrated clear compliance with the instructions: “The report indicates T9 vertebra involvement, which aligns with T4 staging criteria. While the tumor diameter is less than 50 mm and no T3 invasion sites are present, the vertebral involvement confirms T4 staging.”

Finally, to address the issue of misjudgment due to the lack of professional medical knowledge in the model’s practical application, we applied a Background Knowledge Injection strategy. This strategy involved incorporating common error patterns and detailed professional criteria from medical imaging analysis.

The background knowledge serves as a reference for each decision-making process. For instance, we observed that the model tended to incorrectly interpret small lesions in Chinese, such as cysts and thickening, as evidence of distant metastasis. This led to significant errors in M staging.

To address this, we made an additional injection of specific judgment criteria and medical terminology explanations, according to supplementary rules shown in Table 1, such as:

- Knowledge 1: When expressions like “small nodules,” “low-density nodule shadows,” and “density shadows” appear, they should be considered cautiously and cannot be directly inferred as metastatic lesions.
- Knowledge 2: Expressions like “metastatic,” “consider metastasis,” and “suspected metastasis” in other distant organs can be used as evidence for metastatic lesions.

Additionally, we clarified the criteria for key terms such as “tumor invasion,” “cancerous nodules,” and “lymph node metastasis.”

The integration of professional knowledge improved the model’s ability to distinguish between minor abnormalities and actual metastatic lesions. As a result, the misjudgment rate for minor abnormalities decreased from 29% to 6%, while M staging accuracy increased from 70% to 90%.

The prompt optimization framework central to this study, which involves establishing a reasoning baseline, extracting key information, separately addressing T/N/M components, and injecting domain knowledge, was designed as a model-agnostic and universally applicable framework. To ensure a controlled and fair comparison, the iterative prompt optimization was informed by bad-case analysis from both models, and the final prompt set was applied uniformly to all evaluated models. This approach underscores that our primary objective was to validate a generalizable optimization pathway for the clinical task, not to orchestrate a maximally tailored benchmark for any specific model.

It should be acknowledged that the performance of any LLM, including generalist models, such as GPT-4o, could potentially be further enhanced with dedicated, model-specific prompt tuning. This is an inherent consideration when comparing a domain-optimized pipeline with a general-purpose model. Our findings and the proposed framework are positioned within the context of this shared, task-centric optimization objective.

### SFT

After the prompt optimization process, white-box testing results indicated that the model still faces challenges in precise numerical calculations and prompt adherence. Especially for precise numerical comparisons, it is difficult for further prompt optimization to solve this problem due to the lack of related abilities in the original model itself. Therefore, we introduced SFT into the training framework to further enhance model capabilities.

The SFT results showed significant improvements in domain-specific tasks, especially in tumor size measurements and classification. This demonstrated that SFT was an effective strategy for enhancing the model’s numerical computation abilities. Interestingly, we also observed significant differences in the fine-tuning results of different staging models: T staging accuracy improved markedly, while N and M staging did not. The results for M staging (Figure 7) show that the fine-tuned models achieved accuracies comparable to the baseline, with the highest-performing configuration matching the baseline accuracy of 90%, while others were marginally lower. We first clarify that, based on experimental review, neither label noise in the M-staging training data nor the learning rate configuration was the primary cause of this performance plateau. We attribute the observed results primarily to the inherent characteristics of the task and the interplay within our multi-task SFT framework.

First, N and M staging tasks have fewer subcategories compared with T staging. The baseline model, powered by extensive pretraining, may already be near a performance ceiling for tasks heavily reliant on key information retrieval. Consequently, the baseline accuracies for N and M staging were high (89% and 90%, respectively), leaving limited absolute room for improvement via SFT on our dataset. This is particularly relevant for M staging, a binary task (M0 vs M1) with relatively definitive criteria that depend on detecting explicit descriptions of distant metastasis.

Building on this, a more granular technical analysis reveals underlying mechanisms. Beyond the task’s retrieval-intensive nature, the limited number of M1-positive cases in our dataset may constrain the model’s ability to learn the subtle semantic distinctions required (eg, between M1a “contralateral lung nodules” and benign findings). More importantly, within our single-stage, multi-task (T, N, M) joint SFT framework, we hypothesize that conflicting optimization signals may have arisen. T and N staging, as complex tasks demanding deep semantic reorganization, likely generated stronger gradients that dominated the model’s optimization trajectory. In contrast, M staging performance relies heavily on the precise pattern-matching capabilities inherent in the base model. The gradient updates aimed at learning complex semantic mappings for T/N tasks may have inadvertently perturbed these foundational retrieval mechanisms. When the training signal for the M task is relatively limited, this “interference cost” could offset any task-specific gains, resulting in a neutral net performance benefit. It is important to note that we present this “interference cost” as a theoretical hypothesis for the observed results, and it remains to be validated through controlled single-task experiments.

Therefore, the observed plateau in M staging primarily reflects a misalignment between the task’s needs and the full-parameter SFT approach in a multi-task setting, rather than a model flaw. This analysis directly corroborates the rationale for our final hybrid strategy. For M staging, preserving the base model’s strong inherent retrieval ability and guiding it precisely via prompt engineering proved to be a more robust and parameter-efficient solution than full semantic remapping via SFT. Conversely, the significant accuracy gains in T and N staging validate that SFT is highly effective for tasks that require the deep semantic comprehension and reasoning it is designed to enhance.

### Model Evaluation

#### Performance Metrics

We noticed the minor 3-percentage-point drop from white-box to black-box testing. Since we have introduced approximately 7 times the amount of heterogeneous data compared with the target-domain data and balanced the data distribution to ensure essentially consistent coverage and proportions of I to IV clinical stages across both test sets, we believe that the minor difference between the 93% accuracy in white-box testing and 90% accuracy in black-box testing is attributable to normal fluctuations resulting from the test dataset, rather than significant overfitting.

The analysis of 95% CIs for accuracy reveals several noteworthy findings. Following SFT, T staging demonstrated not only substantially improved accuracy but also a markedly narrowed 95% CI, indicating enhanced estimation precision and significantly improved model stability for this task. A similar pattern was observed for N staging, where the model maintained high accuracy while achieving a more stable and narrower CI after SFT. In contrast, GPT-4o exhibited comparatively wider CIs across evaluations, suggesting greater uncertainty in its predictions. Overall, the post-SFT model shows consistently narrowed CIs for both T and N staging, reflecting improved estimation precision and reduced overfitting risk. Notably, in all six comparative scenarios between GLM-4-Air and GPT-4o (encompassing both white-box and black-box evaluations across TNM components), GLM-4-Air consistently demonstrated superior 95% CIs. This pattern suggests improved estimation precision and model stability for these tasks within the evaluated data distribution, which is a desirable property for clinical applications. Particular attention should be paid to the width of the CI for N staging accuracy in the black-box set (77.9% to 91.5%). In comparison, the 95% CIs for both T staging and M staging accuracy in the same black-box set are notably narrower (85.0% to 95.9%). This contrast highlights the greater precision and stability achieved in T and M staging, while the width of the N staging CI reflects not only statistical uncertainty from the sample size but also, and perhaps more critically, the inherent diagnostic challenges specific to imaging-based nodal (N) staging. The assessment of lymph nodes often occupies a clinical “gray zone” between suggestive radiological findings and definitive pathological confirmation, which is a fundamental source of label variability. Additionally, the heterogeneity in nonstandardized descriptive language across different institutional reports particularly affects N staging, contributing to the observed performance range. Therefore, while the relative narrowing of the CI post-SFT confirms improved model stability, its absolute width may, in part, map the expected performance variability when the model confronts the genuine and pronounced complexity inherent to clinical N staging. This understanding underscores that the observed CI width for N staging is not solely a statistical phenomenon but also a reflection of the specific real-world complexity of that task. Consequently, the width of the CI for N staging appropriately highlights the need for future validation in larger, prospective cohorts to more precisely estimate its performance in broader populations.

Based on the analysis of Precision, Recall, and *F*_1_-score for the TNM staging results, we found that for T staging, the assessment of T1a, T1b, T1c, T2a, T2b subcategories primarily relies on the accurate extraction of tumor size information from medical reports, and precise numerical comparison and classification against established staging criteria. The calculated results indicate no substantial differences between the two models in T1 (T1a, T1b, and T1c) and T2 (T2a and T2b) staging outcomes, suggesting comparable capabilities in these fundamental tasks. In T4 staging, which entails assessing involvement of critical structures such as the diaphragm, mediastinum, heart, great vessels, trachea, recurrent laryngeal nerve, esophagus, vertebral body, or carina, GLM-4-Air achieved significantly higher recall than GPT-4o in both black-box (95.6% vs 82.6%) and white-box (92.0% vs 84.0%) settings. This indicates GLM-4-Air’s superior ability to recognize complex or implicit descriptions of invasion. Although GPT-4o showed a slight advantage in black-box precision (95.0% vs 91.7%), its white-box precision dropped sharply to 77.8%, whereas GLM-4-Air maintained 92.0%, demonstrating stronger consistency. For T3 staging, which involves judging chest wall or pericardial invasion, GPT-4o showed marginally higher recall than GLM-4-Air in the black-box set (86.7% vs 80.0%), potentially due to its more accurate and stable interpretation of relevant terminology across diverse medical reports. However, it is noteworthy that GPT-4o’s precision in the white-box set (71.4%) was considerably lower than that of GLM-4-Air (83.3%). Based on a comprehensive analysis and supported by confusion matrix data, we observed that GPT-4o frequently misidentified invasion sites, for instance, misclassifying diaphragmatic invasion as T3-stage involvement. In contrast, GLM-4-Air demonstrated a more balanced judgment, highlighting its advantage in generating reliable clinical references. We also observed that in the white-box test, GLM-4-Air exhibited lower precision in T1a and lower precision and recall in T1c compared with GPT-4o. Although the situation improved in the black-box test, precision in T1c remained lower. Error analysis revealed that most misclassifications by GLM-4-Air involved incorrectly assigning Tx cases as T1a or T1c, often due to errors in extracting tumor diameter from multiple posttreatment measurements. GPT-4o appeared more accurate in such contexts. In response to this identified limitation, we have supplemented the staging rule to specify that tumor diameter should be based on the most recent measurement obtained following the current treatment cycle (Table 1). GLM-4-Air achieved a higher macro-average *F*_1_-score than GPT-4o (black-box: 0.914 vs 0.836; white-box: 0.887 vs 0.839). This superior performance indicates a better balance between precision and recall, which effectively reduces both misclassification and underdiagnosis. This advantage is particularly evident in T4 staging. In the context of NSCLC T staging, this implies that GLM-4-Air provides more comprehensive and reliable identification of various tumor invasion patterns, thereby enhancing diagnostic accuracy. Finally, comparing GLM-4-Air’s overall performance in T staging between white-box and black-box tests revealed no noticeable decline in metrics, indicating stable model output and an absence of overfitting.

For N Staging, in the N0 staging category, GLM-4-Air demonstrated outstanding performance: achieving a recall of 95.6% in white-box testing (vs GPT-4o’s 66.7%) and 97.7% in black-box testing (vs GPT-4o’s 58.1%). This advantage primarily stems from the instructional fine-tuning that incorporates precise medical knowledge: the model strictly adheres to definitive terminology such as “lymph node metastasis” or “enlarged lymph node shadow” to determine metastasis, while maintaining caution against overinterpreting nonspecific descriptions like “small nodule” or “mild FDG uptake.” In contrast, GPT-4o, relying solely on prompt engineering without strict medical logic enforcement, generated numerous false positives (33 cases), substantially reducing its recall. Accurate discrimination between N0 and N1-N3 stages holds direct clinical significance for treatment decisions, such as determining the need for regional lymph node radiotherapy or extensive lymphadenectomy (Table 4 provides error categories). However, in black-box testing, recall for N3 remained suboptimal: GLM-4-Air at 55.6% and GPT-4o at 44.4%. The confusion matrices indicate that both models frequently misclassified N3 as N2 (6 cases by GLM-4-Air, 8 by GPT-4o). This stems from the highly variable and nuanced anatomical descriptions in reports (eg, “contralateral mediastinum” and “contralateral hilum”), which current LLMs struggle to interpret accurately. Future work may involve targeted training or rule-based enhancements to improve performance in such judgments. Additionally, GLM-4-Air misclassified 2 N3 cases as N0 due to its strict adherence to the principle of “classifying as N0 in the absence of definitive metastatic evidence.” While this leads to occasional under-classification, the trade-off is justified given the substantial improvement in N0 recall. Subsequent SFT focusing on complex anatomical descriptions may further enhance N3 staging accuracy. For the Nx staging category, which reflects clinically indeterminate cases, both models showed limited recall: GLM-4-Air and GPT-4o achieved 0% and 8.3%, respectively, in white-box testing, and 50.0% and 13.3% in black-box testing. Analysis of GPT-4o’s outputs suggests a tendency toward conservative assessments: it classified 10 cases that should have been “no metastasis” as “indeterminate (Nx)” in both test sets, significantly lowering its Nx recall. In comparison, GLM-4-Air demonstrated a stronger inclination toward definitive classification and rarely output “indeterminate” or equivalent responses. Finally, in N staging overall, GLM-4-Air exhibited no noticeable performance drop from white-box to black-box testing. Moreover, its macro-average precision, recall, and *F*_1_-score all improved in the black-box setting, indicating stable model generalization without signs of overfitting.

For M staging, based on the *F*_1_-score, GLM-4-Air outperformed GPT-4o in both white-box and black-box evaluations. We attribute this advantage primarily to the effective integration of medical knowledge related to distant tumor metastasis through carefully designed prompts. The results indicate that the medical knowledge acquired during the model’s pretraining phase is insufficient for accurate distant metastasis assessment in our specific clinical context, necessitating supplementary knowledge injection coupled with robust instruction-following capabilities. It is noteworthy that no fine-tuning was performed for GLM-4-Air on M staging tasks, as it already demonstrated competent medical instruction execution. Conversely, should a model exhibit inadequate instruction adherence with complex prompts, instructional fine-tuning could be considered. Overall, GLM-4-Air demonstrated superior efficiency in leveraging medical knowledge embedded in prompts. Analysis of confusion matrices revealed that GPT-4o’s failure to strictly follow prompt instructions resulted in 38 cases of misclassification where nonmetastatic cases were incorrectly identified as metastatic or suspected metastatic. In M staging, GLM-4-Air rigorously adhered to our prompt strategy, which inevitably led to lower performance metrics in certain subcategories compared with GPT-4o. For instance, in white-box M1c staging, GLM-4-Air’s recall (92.6%) was slightly lower than GPT-4o’s (96.3%). Error analysis revealed that GLM-4-Air’s misclassifications stemmed from strict compliance with the prompt rule requiring explicit metastatic evidence in reports, leading to misclassification of M1c as M1a. Under identical prompts, GPT-4o did not adhere to this rule. According to our error classification criteria (Table 4), misclassification between M1c and M1a does not constitute a severe category I error. While strict, detailed prompt strategies may reduce recall in specific scenarios, they ensure better precision. We maintain that a relatively balanced staging performance is more likely to be applicable in real-world clinical settings. Finally, a comprehensive evaluation of GLM-4-Air’s performance in M staging across white-box and black-box tests showed no significant performance degradation. Moreover, macro-average precision, recall, and *F*_1_-score all improved in the black-box setting, indicating stable model output without evidence of overfitting.

Upon analysis of the model’s performance data from both pre- and post-SFT stages, it was observed that in T staging, substantial improvements in *F*_1_-score were observed across the T1a, T1b, T1c, T2a, and T2b subcategories following SFT. This indicates enhanced model capability in numerical discrimination tasks based primarily on tumor size, reflecting SFT’s significant effect in strengthening both fundamental data computation and rule-following abilities. For T2, T3, and T4 staging, where assessment depends not only on tumor dimensions but also on medical understanding of local invasion extent and involved anatomical structures, *F*_1_-scores increased by 57.1%, 41.6%, and 12.0%, respectively, after SFT. These improvements demonstrate that SFT effectively infused relevant medical knowledge, enhancing the model’s comprehension of complex clinical terminology and real-world medical scenarios. Notably, a slight decrease in precision was observed for T2a post-SFT, primarily due to misclassification of some T4 cases as T2a. However, this was accompanied by an 18.7% improvement in T4 precision, which is an outcome aligned with our objective of minimizing category I errors.

In N staging, post-SFT *F*_1_-score improvements for N1, N2, and N3 were 8.9% (0.800 to 0.889), 7.3% (0.882 to 0.955), and 3.0% (0.867 to 0.897), respectively. Although these gains appear modest, they remain practically meaningful given the model’s already high baseline performance in these categories, confirming that SFT effectively enhanced lymph node metastasis assessment through improved medical knowledge integration. For the N0 subcategory, precision slightly decreased (−4.2%) after SFT, but recall increased from 93.3% to 95.6% (+2.3%), with the *F*_1_-score decreasing only marginally (0.009). This shift resulted from the model’s stricter adherence to clinical interpretation strategies post-SFT: classifying cases as positive only when definitive terminology such as “lymph node metastasis” or “enlarged lymph node shadow” was present, while maintaining caution against nonspecific descriptions like “small nodule” or “mild FDG uptake,” thereby effectively reducing false positives. Overall, the minimal *F*_1_-score reduction in N0 is substantially outweighed by the collective gains in N1-N3, consistent with our expectations.

In conclusion, SFT of GLM-4-Air not only improved computational accuracy in rule-driven tasks but also significantly enhanced discriminative capability in complex medical reasoning scenarios.

#### Statistical Comparison

In the TNM staging task, model performance exhibited distinct stage-dependent variations between white-box and black-box evaluations. For T staging, no statistically significant differences were observed between models in either test set (*P*>.05). This likely stems from T staging’s reliance on objective, quantifiable imaging features such as tumor size, which is a task with well-defined criteria and high interpretive consistency. The observed effect sizes for white-box (*ω*=0.374) and black-box (*ω*=0.378) evaluations both reached medium-to-large magnitudes according to conventional benchmarks. Although these effect sizes should be interpreted with caution due to the nonsignificant differences, they may nonetheless indicate exploratory trends worthy of future investigation with larger sample sizes.

In contrast, for N staging, highly significant differences (*P*<.05) were consistently demonstrated across both test sets, indicating statistically meaningful performance disparities. This suggests fundamental differences in how the models interpret regional lymph node metastasis status.

For M staging, white-box evaluation showed significant differences (*P*<.05). In the black-box evaluation, where the difference did not reach statistical significance (*P*=.10), the medium-to-large effect size (Cohen ω=0.399) suggests a potential trend, which points to a possible performance difference in detecting distant metastasis evidence in model behavior.

In summary, model performance divergence primarily emerged in tasks requiring complex clinical reasoning (eg, lymph node or distant metastasis assessment), whereas performance converged in tasks dependent on explicit numerical criteria (eg, T staging).

#### Clinical Impact Assessment

##### Overview

Based on the comprehensive analysis of all error types for both models across white-box and black-box evaluations, GLM-4-Air demonstrates substantial advantages in the TNM staging task, which can be summarized as the following 2 aspects.

##### GLM-4-Air's Advantages in TNM Staging

###### Superior Overall Error Control

Across all 18 comparison metrics (3 stages / 2 test sets / 3 error categories), GLM-4-Air outperformed GPT-4o in 14 metrics, matched its performance in 2 metrics (category II errors in M staging, with 0 errors in white-box and black-box for both models), and showed marginally higher errors in only 2 metrics (category II errors in black-box T staging and category III errors in white-box T staging, with 1 additional error each). This overall advantage primarily stems from the heterogeneous data training strategy used during SFT for T and N staging, coupled with enhanced instruction adherence (strict implementation of our stringent strategy), which collectively improved the model’s discriminative capability for medical features.

###### Significant Effectiveness in Controlling Critical Error Types

The core strategy of this study focuses on effectively reducing category I errors. GLM-4-Air completely reduced category I errors to 0 in M staging, whereas GPT-4o committed 8 and 2 such errors in white-box and black-box tests, respectively. This discrepancy carries crucial clinical implications: misclassifying M0 (no distant metastasis) as M1 (distant metastasis present) would incorrectly upgrade the clinical stage from III to IV, consequently shifting the treatment strategy from surgery-based comprehensive therapy to primarily palliative care. Similarly, in N staging, GLM-4-Air significantly reduced category I errors involving the misclassification of N0 as N2. Such errors could lead to overdiagnosis in clinical practice, subjecting patients who do not require lymph node dissection or radiotherapy to unnecessary medical risks and financial burdens. Despite the overall superior performance, minor fluctuations were observed in GLM-4-Air’s category II errors in black-box testing and category III errors in white-box testing. Analysis of these specific error cases revealed that most stemmed from inaccuracies in standardized comparison of tumor size data, which is a challenge also observed in GPT-4o’s errors. Although the performance gap with GPT-4o in these aspects is minimal, we will continue to explore methods to further enhance these capabilities.

###### Conclusions

In conclusion, while maintaining stringent control over category I errors, GLM-4-Air also preserves advantages over GPT-4o in most category II and III error metrics. This indicates that the model achieves high safety standards without compromising overall accuracy, rendering GLM-4-Air particularly suitable for practical TNM staging applications with enhanced reliability and performance.

### Rationale for Strict M-Staging Strategy

In addition, based on the comparative analysis of strict versus lenient interpretation strategies for M staging, the strict strategy proved highly effective in minimizing false positives while not significantly increasing false negatives. In contrast, the lenient strategy resulted in a substantially higher rate of false positives.

The adoption of the strict interpretation strategy for M staging aims to minimize false positives without compromising the need to maintain a low rate of false negatives and is grounded in specific clinical imperatives. From a clinical perspective, the high false positive rate observed under the lenient strategy is unacceptable. For instance, benign findings commonly described in reports, such as cysts in the kidneys or liver in older patients, could be misclassified as M1 (Stage IV) metastases under lenient rules. This could immediately induce unnecessary patient anxiety and trigger a cascade of inappropriate clinical actions. More critically, a false-positive call at this diagnostic juncture can fundamentally misdirect the primary treatment pathway, potentially steering a patient eligible for curative-intent therapy (eg, surgery or definitive chemoradiation) toward an erroneous palliative systemic approach. Furthermore, it compels a series of costly, invasive, and burdensome confirmatory investigations (eg, advanced imaging, biopsies) without clinical merit.

This strategic choice is also aligned with the linguistic reality of Chinese radiology reports, which often use broad or cautious terminology. The strict interpretation acts as a necessary filter against this inherent ambiguity, serving as a “localized calibration” technique in this context. Its core function is to correct the tendency of general-purpose LLMs to over-interpret ambiguous expressions in the absence of domain-specific fine-tuning, an enhancement generally applicable within Chinese clinical practice. It is crucial to acknowledge that this calibrated approach, while effective, is not a universal reasoning logic. It could theoretically introduce a risk of false negatives in scenarios where conservatively phrased reports use descriptive terminology. This technique complements the AJCC-based rule supplements we implemented.

Therefore, while sensitivity remains important, our strategy is designed to ensure that the model’s outputs provide highly credible and actionable decision support. A crucial caveat is that using such a strategy requires preliminary testing and calibration based on the target language or dataset. The decision to apply a strict strategy (to correct LLM over-interpretation) versus a lenient one (to correct LLM under-interpretation) should be guided by initial validation, as the latter scenario is also possible in other linguistic contexts or specific datasets. The strict strategy for M staging in this study thus represents a calibrated balance, optimizing the model for reliable integration into high-stakes clinical workflows.

### Cost-Effective Evaluation

This evaluation examines the operational efficiency and deployment feasibility of our optimized GLM-4-Air model. Through domain-specific optimization, the 32B-parameter GLM-4-Air model can achieve high staging accuracy while demonstrating a practical efficiency profile. A key practical implication of this study is the demonstration that a specialized, moderately sized language model can perform complex TNM staging with high accuracy using cost-effective, consumer-grade hardware. Specifically, our local deployment benchmark achieved low per-component latencies on 4 RTX 4090 GPUs, confirming that the model’s performance profile is acceptable and suitable for integration into real-world clinical workflows without requiring prohibitive computational infrastructure. While this study demonstrates the cost-effectiveness of the 32B-parameter GLM-4-Air model within our hybrid framework, we acknowledge that the broader claim of hardware accessibility is not exclusive to this specific model. Other competitively performant “lightweight” models (eg, GPT-4o-mini, Llama-3-70B), which share a similar order-of-magnitude parameter scale, could potentially offer comparable reductions in computational resource demand and deployment cost. While the assessment of other lightweight models was beyond the scope of this study, a systematic comparison among them represents a direction for future research.

Furthermore, the Cost-effective Evaluation suggests a trend of complementary strengths between large general-purpose models and smaller, domain-adapted models in the medical TNM staging task. On one hand, GPT-4o, a general-purpose LLM, demonstrates high conciseness in tasks with shorter reasoning chains, such as N staging. Its powerful linguistic generalization capabilities enable it to rapidly extract key information. On the other hand, for complex tasks like T staging, which require in-depth parsing of anatomical relationships and multimodal clinical descriptions, GLM-4-Air, enhanced by targeted medical SFT, establishes more stable and interpretable reasoning pathways. While large-parameter models excel in cross-domain generalization and flexible response for open scenarios, smaller-parameter models like GLM-4-Air are potentially better suited for high-reliability specialized tasks. They can potentially deliver lower overall latency and higher deployment cost-effectiveness while ensuring reasoning accuracy and completeness, with a proper training framework.

### Limitations

While the performance of our model is robust on the held-out test sets, we acknowledge that the generalizability of any AI model trained on a dataset of this scale (292 fine-tuning cases) warrants careful discussion. The primary consideration is its performance on data from wider distributions, such as more institutions with variations in reporting styles. Furthermore, our dataset was sourced exclusively from the proprietary medical record-management platform reliant on user-initiated uploads. This data acquisition method may introduce potential selection biases: the user population likely possesses higher digital literacy and health awareness, potentially limiting representativeness in terms of age or socioeconomic status; the act of voluntary uploading may also over-represent patients with more complex conditions, unresolved diagnostic concerns, a willingness to seek second opinions, or experiences with multi-institutional care in the dataset. These specific characteristics suggest that the patient profile in our study may differ from that of a general hospital cohort, which would typically include a higher proportion of patients with lower digital literacy (often associated with advanced age or lower socioeconomic status) and earlier-stage disease. The model’s staging accuracy for these specific subgroups requires further validation. Additionally, as uploaded data typically consists of single or nonconsecutive medical records, it lacks the temporal continuity and completeness in documenting disease progression compared with the structured, longitudinal records within a hospital information system. This difference in information presentation implies that the model may face challenges in inference and integration when processing richer contextual reports. Consequently, while the model demonstrates robust performance within the represented data distribution, its generalizability to passively collected, consecutive hospital-wide cohorts could be further explored in future work. Caution is advised when interpreting performance metrics for subclasses with a low number of cases. The high recall rates observed in some specific subgroups primarily reflect performance on that particular test subset and are subject to statistical variability due to limited sample size. It is also crucial to emphasize that an awareness of these potential biases directly informed our study design. To proactively enhance model robustness and generalizability within this context, we integrated 2 core methodological choices: the curation of rigorously balanced test sets (Figure 2) to ensure reliable evaluation, and the use of a heterogeneous data regimen during SFT to significantly reduce the risk of overfitting.

Another topic we would like to discuss is language generalizability. In this study, the model was exclusively trained and validated on medical reports in Chinese, and we acknowledge its potential limitations in generalizing across languages and health care systems. The unique expression logic, terminology, and writing structure inherent to Chinese clinical texts may restrict the model’s direct applicability to other languages, such as English, or different medical environments. A noteworthy point for discussion is the systematic difference in linguistic precision between Chinese and English medical language. Chinese reports often use broad-meaning or imprecise terms; for instance, the word “侵犯” can encompass various pathological states from adhesion to infiltration, with its specific interpretation highly dependent on context and the radiologist’s personal expression habits. In contrast, the mature system of English medical literature, shaped by long-term standardization, tends to use more discriminative terminology, such as “abutment,” “invasion,” and “encasement” to describe different degrees of involvement. These terms often have more direct correspondence with definitions in the AJCC staging guidelines. This linguistic characteristic suggests that while our current model is specialized for the Chinese context, adapting its core competency to the potentially more standardized terminology of English environments might present relatively manageable semantic understanding challenges, and the model may need recalibration for health care systems with more precise reporting standards.

### Future Work

Building upon the generalizable foundation established in this work, our future research will focus on extending the model’s robustness to multi-institutional environments. The strategies outlined below are presented as logical and powerful extensions of this study:

1. More collaboration: To directly assess and enhance performance across varied clinical settings, we plan to establish more collaborations to build larger, more representative datasets spanning different languages and health care systems. A key component will be the development of unified data inclusion standards and quality control metrics. We will continuously assess the collaboration’s effectiveness and optimize the workflow and technical protocols to ensure the creation of high-quality, federated data resources.
2. Systematic, knowledge-guided data augmentation: We will develop a systematic data augmentation pipeline to explicitly train the model to increase invariance to clinical language variations. This will include:
  1. Guideline-based semantic paraphrasing: Leveraging authoritative resources like the AJCC Cancer Staging Manual, we will systematically create a thesaurus of medical synonyms and sentence templates. For instance, for ‘tumor invasion of the visceral pleura,’ we will generate medically accurate alternatives like ‘microscopic involvement of the visceral pleura’ or ‘lesion involving the visceral pleural surface.’ This directly enhances robustness to variations in physicians’ dictation styles.
  2. LLM-assisted synthetic data generation: Using high-quality reports as seeds, we will use LLMs under strict constraints for conditional text generation. This will efficiently produce a large volume of coherent and logically sound synthetic reports. This approach will be particularly targeted towards under-represented or easily confused staging categories (eg, T1c vs T2a) identified in our error analysis, allowing us to strategically address specific model weaknesses rather than blindly expanding data.

In terms of language generalizability, to systematically enable effective deployment in broader scenarios, we propose the following three actionable technical pathways:

1. International collaboration: Establish collaborations with overseas medical institutions to acquire ethically approved, deidentified medical reports in English (or other languages). This will build a multi-center, cross-lingual dataset for model adaptation training and external validation, enhancing its ability to extract key information from reports with different structures and terminology.
2. Optimized modular and decoupled design: Decouple the model’s language understanding component from its medical reasoning component. The language understanding module would focus on accurately extracting key medical entities and their attributes from raw text. The medical reasoning module, leveraging shared clinical knowledge, would then perform logical staging based on these structured findings, strictly adhering to TNM guidelines. This design ensures the reasoning component’s generalizability across languages. When adapting to a new language, only the entity recognition capability of the language module needs training, significantly improving cross-lingual adaptability and reducing data requirements.
3. Construction of a cross-lingual clinical terminology mapping atlas: Systematically organize key phrases and imprecise terms related to TNM staging in specific language reports, establishing precise mappings to international standard terminologies like SNOMED CT (Systematized Nomenclature of Medicine - Clinical Terms) or Unified Medical Language System. This work will lay the foundation for achieving semantic alignment of clinical texts across languages.

In summary, by implementing this technical roadmap centered on global collaboration, modular design, and cross-lingual terminology alignment, we can systematically enhance the model’s generalizability, ultimately developing it into a practical tool for global, multi-lingual clinical environments.

Given the inherent complexity and subjective nature of cancer staging, we recognize that a prospective comparison between the model’s performance and the independent interpretations of multiple clinicians on new, unseen cases would provide a more ecologically valid assessment of its clinical utility. Such a study design, where the model’s outputs and individual expert annotations are collected in parallel without a pre-established consensus, would more accurately simulate real-world deployment scenarios. This approach would not only allow for a robust benchmarking against the natural variation inherent in clinical practice but also help identify specific contexts where the model’s reasoning aligns with or diverges from human experts. We plan to implement this in future research, potentially involving a larger and more diverse panel of annotators from multiple institutions. This direction is particularly significant for validating AI assistants in complex, subjective tasks like cancer staging, and its methodology could be extended to other high-stakes clinical decision support applications where interexpert variability is a key consideration.

### Conclusions

In conclusion, this study establishes a robust and efficient framework for automating TNM staging in NSCLC by leveraging the GLM-4-Air model enhanced through advanced prompt engineering and selectively applies SFT to reasoning-heavy tasks (T/N) while leveraging the baseline model for retrieval-centric tasks (M). This task-dependent optimization strategy highlights that the value of fine-tuning is not universal but varies with the nature of the clinical subtask, which is a key insight for future clinical AI development. The finalized model demonstrated superior performance over a leading commercial model, GPT-4o, achieving higher accuracy, particularly in complex staging decisions, while simultaneously reducing the incidence of clinically critical errors. Its design, which emphasizes reasoning transparency and adherence to clinical guidelines, ensures reliability. The exceptional cost efficiency of the solution further underscores its viability for scalable deployment.

This work also provides a validated pathway toward standardizing and augmenting cancer staging processes, with the immediate potential to improve consistency in treatment planning and to expand access to expert-level staging support in diverse health care settings.

## Supplementary material

10.2196/77988 Multimedia Appendix 1 Distribution of data set by imaging modality, language and hospital sources.

10.2196/77988 Multimedia Appendix 2 Distribution of T, N, M staging of the Data Set.

10.2196/77988 Multimedia Appendix 3 Domain of the Auxiliary Instruction Samples.

10.2196/77988 Multimedia Appendix 4 Result of post-OCR Chinese character error rate test and Examples of common OCR error types.

10.2196/77988 Multimedia Appendix 5 Deidentified example dataset.

10.2196/77988 Multimedia Appendix 6 Sample of T Staging.

10.2196/77988 Multimedia Appendix 7 Sample of N Staging.

10.2196/77988 Multimedia Appendix 8 Sample of M Staging.

10.2196/77988 Multimedia Appendix 9 Model Card.

## Abbreviations

AI: artificial intelligence
AJCC: American Joint Committee on Cancer
CoT: chain-of-thought
GLM: general language model
LLM: large language model
LoRA: low-rank adaptation
M: distant metastasis status
N: regional lymph node involvement
NSCLC: non-small cell lung cancer
OCR: optical character recognition
SFT: supervised fine-tuning
SNOMED CT: Systematized Nomenclature of Medicine - Clinical Terms
T: primary tumor characteristics
TNM: tumor-node-metastasis
