# Supplementary material for: Augmenting Large Language Model With Prompt Engineering and Supervised Fine-Tuning in Non-Small Cell Lung Cancer Tumor-Node-Metastasis Staging: Framework Development and Validation
Source: JMIR AI. 2026 Apr 15;5:e77988. doi: 10.2196/77988 (PMC13082344; doi:10.2196/77988)
Supplement: Multimedia Appendix 4 [file ai-v5-e77988-s004.pdf]

Result of post-OCR Chinese character error rate test and Examples of common OCR error types

1. Result of post-OCR Chinese character error rate test

Dataset	White-Box（13 cases）	Black-Box（14 cases）
**Number of Reports With Hospital Name**	7	8
**Number of Reports from 'Unknown' Category**	6	6
**Total Chinese Characters**	10168	13289
**Total Erroneous Characters (Excluding Punctuation)**	27	29
**Character Error Rate (Excluding Punctuation)**	0.26%	0.22%
**Impact of OCR Errors on Tumor Size Recognition**	No Impact	No Impact
**Impact of OCR Errors on Lymph Node Status Recognition**	No Impact	No Impact
**Impact of OCR Errors on Distant Metastasis Recognition**	No Impact	No Impact

The Chinese character error rates for the white-box and black-box settings were 0.26% and 0.22%, respectively, which is consistent with the data provided by the supplier [22].
Manual review confirmed that the erroneous Chinese characters did not affect the accuracy of the key information input to the model, and consequently, they had no impact on the model's overall interpretation of the reports.
Similarly, manual review of the four common OCR error types concluded that none of them affected the accuracy of the key information input to the model, and consequently, none impacted the model's overall interpretation of the reports.

2. Examples of Four common OCR error types

(1) Erroneous line breaks

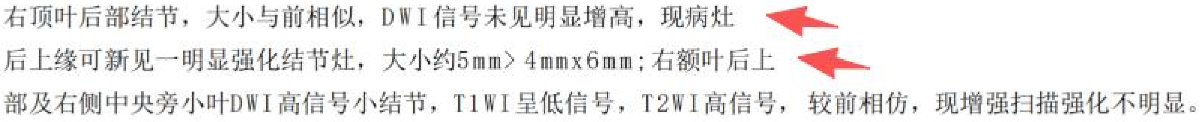
右顶叶后部结节，大小与前相似，DWI信号未见明显增高，现病灶
后上缘可新见一明显强化结节灶，大小约5mm> 4mmx6mm;右额叶后上
部及右侧中央旁小叶DWI高信号小结节，T1WI呈低信号，T2WI高信号，较前相仿，现增强扫描强化不明显。

(2) Spurious spaces between characters

Case: 检查报告 CT增强造影剂;CT胸部平扫+增 强;影像增强扫描用耗材(B套) 报告单号 报告医师审核医师
检查项目 CT增强造影剂;CT胸部平扫+增强;影像增强扫描用耗 材(B套) 检查所见
两侧胸廓对称。两肺支气管血管束增多，右肺中叶纤 维条索影。左肺下叶肺门下方见类圆形软组织密度
影，大小约2.9*2.5cm，增强后为轻度不均匀强化。左肺上叶见一钙化结节。右肺(SE201，IM29、
19)见实性结节，长径约3mm。气管居中，气管、 主支气管开口通畅。纵隔内及两肺门未见明显肿大淋
巴结影。双侧胸膜光整。双侧胸腔未见积液。胆囊体 积增大。 检查结论/诊断

(3) Omitted/misplaced punctuation

[illegible]
枚实性小结节，较前变化不大。 看病，们要时增强扫描进一步检查。，部 为纤维灶可能，较前减少。

(4) Poor paragraph segmentation

Case: 16:38 < 检查报告 o _0057_颈椎64层CT 胸部64层CT3 就诊人 开方医 性别: 年龄: 开方科室:呼吸内二门诊
报告时间:2022/5/9 14:45:03 检查部位 骨骼 检查所见 双侧锁骨上见小淋巴结，大者短径约6mm，双侧胸
廓对称，纵隔内结构清晰，纵隔见多发增大淋巴结， 大者短径约10mm(图2-17)。左肺门不大，各叶支
气管通畅，右肺上叶可见一不规则密度增高肿块影， 边缘毛糙，大小约23x46mm(图2-16)，上叶支气
管狭窄，右肺另见片索影，左肺野内未见异常密度 影。双侧胸膜未见增厚，未见胸水。骨窗部分椎体高
密度。双侧颈部见多发小淋巴结。甲状腺密度不均 匀，咽部未见异常。 诊断意见
右肺上叶癌，较前减小纵隔多发淋巴结肿大，双侧 锁骨上小淋巴结，减小右肺炎症椎体高密度，结合
骨ECT 检查双侧颈部小淋巴结甲状腺改变，结合彩 超 检查方法
11:59 < 检查报告 o _0012_胸部64层CT41 就诊 性分 开方科室:呼吸内一门诊 报告时间:2022/3/26 11:44:41 检查部位
