# Supplementary material for: Augmenting Large Language Model With Prompt Engineering and Supervised Fine-Tuning in Non-Small Cell Lung Cancer Tumor-Node-Metastasis Staging: Framework Development and Validation
Source: JMIR AI. 2026 Apr 15;5:e77988. doi: 10.2196/77988 (PMC13082344; doi:10.2196/77988)
Supplement: Multimedia Appendix 5 [file ai-v5-e77988-s005.pdf]

De-identified example dataset

ID: 1

Case: 检查报告 CT增强造影剂;CT胸部平扫+增 强;影像增强扫描用耗材(B 套) 报告单号 报告医师审核医师
检查项目 CT增强造影剂;CT胸部平扫+增强;影像增强扫描用耗 材(B套) 检查所见
两侧胸廓对称。两肺支气管血管束增多，右肺中叶纤 维条索影。左肺下叶肺门下方见类圆形软组织密度
影，大小约2.9*2.5cm，增强后为轻度不均匀强化。 左肺上叶见一钙化结节。右肺(SE201，IM29、
19)见实性结节，长径约3mm。气管居中，气管、 主支气管开口通畅。纵隔内及两肺门未见明显肿大淋
巴结影。双侧胸膜光整。双侧胸腔未见积液。胆囊体 积增大。 检查结论/诊断
左肺下叶肺门旁占位，考虑MT，建议复查。右肺实 性微小结节，随诊。右肺中叶纤维灶。左肺上叶钙
化灶。大胆囊。
PET/CT影像诊断报告 PET检查号: 性别: 女检查日期: 住院号: 床号: 显像剂: 18F-FDG 药物剂量: 7.54mCi 临床诊断肺
部阴影 影像所见: 受检者空腹，注射显像剂18F-FDG(18氟-脱氧葡萄糖)后行PET/CT显像(范围从头
颅至股骨上段)。 PET显像示脑皮质、双侧基底节区、丘脑、小脑等结构显像剂分布基本对称，未见
显像剂摄取异常增高或减低。CT平扫示双侧大脑半球对称，脑中线结构居中，各脑室、
脑池未见明显扩大，脑沟、脑裂未见明显增宽，颅内未见明显占位性病灶。
PET显像示颅底、鼻咽部、颌面部、颈部以及锁骨上下区未见显像剂摄取异常增高
影。CT平扫示甲状腺形态、大小如常，其内密度欠均匀;颅底、鼻咽部、颌面部、颈
部以及锁骨上下区未见明显占位性病灶。
PET显像示左肺下叶近肺门旁见团状显像剂浓聚影，SUVmax=12.2;纵隔、双侧肺
门、双侧乳腺、双侧腋窝及胸壁未见显像剂摄取异常增高影;左心室心肌见显像剂生
理性摄取。CT平扫示双侧胸廓对称,气管居中，气管、主支气管开口通畅;左肺下叶
近肺门旁见肿块影,大小约3.5*2.8cm;右肺中叶见微小结节影及纤维条索影;纵隔、
双侧肺门及双侧腋窝未见肿大淋巴结影;双侧胸腔未见明显积液影;双侧乳腺未见明 显占位性病灶。
PET显像示肝脏实质内未见显像剂摄取异常增高影;胃充盈可，胃壁见显像剂生理
性分布;脾脏、胰腺、双肾、双侧肾上腺及腹膜后未见显像剂摄取异常增高影;肠道
见显像剂生理性分布。CT平扫示肝脏形态、大小未见明显异常，表面光整，肝实质内
未见明显异常密度影;胆囊增大，其内似见高密度影;肝内外胆管未见明显扩张;胃
充盈可，胃壁未见局部异常增厚:胰腺、脾脏、双肾及双侧肾上腺形态、大小及密度
未见明显异常;腹腔及腹膜后未见明显占位性病灶及肿大淋巴结影;腹腔未见明显积 液影。
PET显像示盆腔及双侧腹股沟区未见显像剂摄取异常增高影;肠道见显像剂生理性
分布:膀胱内见显像剂生理性蓄积。CT平扫示子宫及双侧附件区缺如;盆腔及腹股沟
区未见明显占位性病灶及肿大淋巴结影;膀胱充盈欠佳，壁光整，未见局部异常增厚; 盆腔内未见积液影。
PET显像示所见骨骼未见显像剂摄取异常增高影。CT平扫示所见骨骼未见明显骨质
破坏;脊柱部分椎体缘及椎小关节见增生硬化改变。 影像诊断:
1.左肺下叶近肺门旁肿块伴FDG代谢异常增高，倾向恶性病变，请结合临床;余所见部
位未见FDG代谢异常增高灶。 2.甲状腺两叶密度欠均,请结合超声;右肺中叶微小结节，随诊;右肺中叶纤维灶;
大胆囊，胆结石可能，请结合超声;子宫、附件全切术后;部分颈胸腰椎退变。 报告:审核:

ID: 2
Case: 病历号： 姓名： 性别： 男
科别：胸外科 床号： 核素：18F 药物：FDG
给药途径：静脉注射 血糖：4.8 检查项目：18F-FDG全身断层显像
临床诊断：肺占位性病变
病史及检查目的：
患者长期咳喘，最近加重2 月余，外院胸部CT 检查：左肺中央型肺癌伴左肺门、纵隔淋巴结转移可能。现为进一步评估全身情况完善PET-CT 检查。
检查所见：
检查过程：
空腹6h 以上，静脉注射18F-FDG50-60min 后行全身PET/CT 断层显像，影像清晰。检查范围从头颅至股骨上1/3 处。
图像所见：
头部：右侧额叶可见类圆形低密度影，径约2.01cm，中央 FDG 代谢缺失，边缘FDG 代谢增高，SUVmax 为8.75，部分延伸至邻近右侧侧脑室前角旁；余大脑各叶、双侧基底节、丘脑、小脑及脑干密度、形态未见明显异常，放射性分布均匀、对称。诸脑室、脑池、脑沟、脑裂未见增宽、扩张。中线结构居中。双侧上颌窦粘膜增厚；双侧额窦、蝶窦及筛窦粘膜未见明显增厚，放射性分布未见异常。
颈部：鼻咽顶后壁及双侧壁未见明显增厚，咽隐窝及咽旁间隙清晰，放射性分布未见明显异常。口咽及喉咽密度未见明显异常，放射性分布未见异常增高。双叶甲状腺密度均匀，放射性分布未见异常。双侧颌下、颈部及锁骨上淋巴结未见明显肿大及异常放射性摄取。
胸部：双肺纹理增多，左肺门旁可见软组织肿块，大小约5.77cm×6.05cm，可见分叶和毛刺，左肺下叶支气管狭窄阻塞，FDG 代谢增高，SUVmax 为15.48；右肺上叶见散在呈簇状分布结节影，其中较大结节位于尖段支气管旁，直径约0.7cm，伴 FDG 代谢增高，SUVmax 为5.81，余结节部分FDG 代谢轻度增高，SUVmax 为1.68；左肺上叶可见斑片状密度增高影；双肺另见散在小结节，FDG 代谢未见增高；双肺散在囊状透亮影。左侧胸腔内可见液体密度影。纵隔（1L、2L、4L、5、7 区）及左肺门多发淋巴结肿大伴 FDG 代谢增高，大者径约3.9cm（PET 序列测量），SUVmax 为17.18；双侧腋窝淋巴结未见明显肿大及异常放射性摄取。食管管壁未见明显增厚及异常放射性分布。心包少量积液。
腹部：肝脏形态、大小、密度及放射性分布未见明显异常；肝内、外胆管未见扩张。胆囊壁未见异常增厚，胆囊内可见多发结节状致密影。胃壁未见明显增厚及异常放射性摄取增高。脾脏大小正常，放射性分布均匀。胰腺形态、大小、密度未见明显异常，胰管未见扩张，未见异常放射性分布。右肾上腺增粗伴FDG 代谢增高，SUVmax 为7.71。双肾实质未见异常密度影，肾盂肾盏未见明显扩张，放射性分布未见异常增高。升结肠可见FDG 代谢增高，SUVmax 为6.67。腹腔内及腹膜后未见明显肿大淋巴结及异常放射性分布。
盆腔：膀胱充盈良好，膀胱壁未见明显增厚，内未见异常密度影。前列腺形态增大，内可见结节状致密影，未见FDG 异常摄取。双侧精囊腺未见FDG 异常摄取。双侧腹股沟未见明显肿大淋巴结及异常放射性分布。
其他：C7 椎体、T1 及L4 左侧椎弓板及棘突、S1 椎体、左侧第11 后肋、左侧肩胛骨、左侧髂骨、左侧髋臼、右侧股骨头及股骨近段可见片状、结节状FDG 代谢增高，SUVmax 为12.57，同机CT 骨质密度改变不显著；多椎体及附件见骨质增生、硬化，部分伴骨赘形成；余所见颅骨、各躯干骨以及四肢骨密度及放射性分布未见异常。

诊断意见：
1.a.左肺门旁软组织肿块伴 FDG 代谢增高，考虑MT；左侧胸腔少量积液；
b.纵隔（1L、2L、4L、5、7 区）及左肺门多发淋巴结肿大伴FDG 代谢增高，考虑转移；
c.上述骨多发 FDG 代谢增高，考虑转移；
d.右侧额叶类圆形低密度影伴边缘 FDG 代谢增高，考虑转移，请结合MR 检查；
e.右肺上叶尖段支气管旁结节伴 FDG 代谢增高，MT 待排；两肺另见散在结节倾向陈旧性，请随诊；左肺上叶慢性炎症；双肺肺气肿；心包少量积液；
2.胆囊多发结石；右肾上腺增生考虑；升结肠炎性或生理性摄取；前列腺增生伴钙化；
3.脊柱退变；
4.老年性脑改变；双侧上颌窦炎。
报告医生：审核医生：
本报告仅供本院医生参考，不作证明用。

ID:3
Case: 全国 PET-CT诊断报告 H R 检查号:姓 名 ： 性别:男 科室:中医一门诊 显像剂:18F-FDG 采集方式:3D/ Helical
剂量:8.3mCi 临床诊断:肺结节 检查所见
空腹6h以上，静脉注射18F-FDG，静息60min后行全身PET-CT断层显像，影像清晰。
双侧脑实质密度、形态正常，放射性分布均匀、对称，诸脑室、脑池、脑沟、脑裂未
见异常，中线结构居中。鼻咽、口咽、喉咽未见异常。额窦、蝶窦、筛窦及双侧上颌窦结
构及放射性分布未见明显异常。双侧下鼻甲粘膜增厚放射性分布未见异常。甲状腺放射性
分布未见异常。颈部未见肿大淋巴结及异常放射性分布。
双肺透亮度可，纹理清晰，气管及主支气管通畅。右肺上叶后段见混杂磨玻璃密度结
节影放射性分布稍浓聚，SUVmax约2.2，大小约1.5cmX2.6cm，边界欠清。右肺上叶另见
高密度小结节影，放射性分布未见异常。左肺上叶舌段见高密度斑片状影放射性分布未见
异常。余双肺野及胸壁未见确切病灶和异常放射性分布。纵隔(7区)见钙化结节影。双肺门及双侧腋窝未见明显肿大淋巴结
及异常放射性分布。
肝左叶见小片状稍低密度影放射性分布未见异常。余肝脏形态、结构及放射性分布未见明显异常，SUVmax约3.1。
肝门结构及放射性分布未见明显异常。肝内、外胆管及胆总管未见扩张。胆囊、胃及十二指肠放射性分布未见异常。
脾周见软组织结节影放射性分布
未见异常，大小约1.5cmX1.8cm。左肾见小囊性低密度影放射性分布稀疏。右肾见点状致
密影。脾脏、胰腺及双侧肾上腺形态、结构及放射性分布未见异常。腹腔及腹膜后未见肿
大淋巴结及异常放射性分布。
肠管节段性放射性分布浓聚，SUVmax约4.6。膀胱、余肠管、前列腺等形态、结构及
放射性分布未见明显异常。双侧髂血管旁及双侧腹股沟未见明显肿大淋巴结及异常放射性
分布。双侧阴囊内见少许液体密度影。
额骨右侧骨质局部缺损放射性分布未见异常。脊柱部分椎体边缘骨质增生、硬化。L3
椎体前缘放射性分布浓聚，SUVmax约4.2。余所见颅骨、躯干骨及四肢骨放射性分布未见 明显异常。
全国 PET-CT诊断报告 HR 检查号 姓 性别:男科室:中医一门诊 检查所见(接上页) 印象:

1.右肺上叶混杂磨玻璃结节代谢稍活跃，疑恶性病变，建议结合病理;右肺上叶另见高密度小结节代谢未见异常，建议随诊观察。
2.左肺上叶舌段炎症;纵隔钙化灶。
3.双侧下鼻甲肥大。
4.肝左叶小片状稍低密度影代谢未见异常，考虑良性病变;副脾;右肾小结石;左肾囊 肿。
5.肠管节段性代谢活跃，考虑生理性摄取;双侧睾丸鞘膜腔少量积液。
6.额骨右份骨质局部缺损代谢未见异常;脊柱退行性变;L3椎体前缘代谢活跃，考虑炎 性。

ID:4

Case: CT 诊断报告-数字签名 病历号:病床号:登记时间性别:男 科室:入院准备中心

检查部位:胸部CT增强(胸外科手术计划专用) 检查设备:Siemens Definition AS 40 影像所见:

两肺纹理清晰，左肺下叶占位，较大横截面积约52*38mm，边缘可见毛刺分叶，周围可见斑片模糊影，增强后呈不均匀渐进性强化，内可见无强化坏死灶。病灶周围可见较大结节影(Se5 Im197)，约10mm，界尚清。余两肺散在小结节，约2-4mm，界清。两肺下叶胸膜下少许斑片模糊影，两肺散在条索影，余肺野未见明显异常密度灶，气管通畅，纵隔内见稍饱满淋巴结影，增强后未见明显强化。两侧胸膜局部增厚，胸廓骨性结构完整。升主动脉稍增宽，约40mm。主动脉壁 局部钙化。

影像诊断: 左下肺占位，MT需考虑，邻近较多结节，转移待排，请复查。 余两肺小结节，建议随访。

两肺下叶少许炎性灶，两肺散在纤维灶。 胸膜增厚。升主动脉稍增宽。动脉硬化。

附见:右肾囊肿，左肾小结石。 申请:检查: 书写: 审核: 报告时间

****此报告仅供本院医生参考，不作证明用******

ID: 5

Case: CT 诊断报告 数字签名 病历号: 病床号:性别:男 科室:胸外科 检查部位:胸部CT增强 检查设备:GE Optima CT620 影像所见: 右肺门软组织肿块，约40mm*35mm，呈轻中度持续强化，邻近支气管壁增厚、右肺中间段支气管狭窄、右下肺背段支气管狭窄闭塞，局部肺动脉分支受累狭窄。右下肺后基底段沿支气管走行强化软组织影。纵隔及右肺门淋巴结稍肿大。两肺胸膜下少量网格状病变。气管通畅。主动脉及冠脉壁钙化。 影像诊断:

右肺门软组织肿块，MT考虑，右下肺后基底段软组织影考虑肺内转移可能，纵隔及右肺门淋巴结稍肿大，建议进一步检查。 两肺胸膜下轻度间质性炎。 动脉硬化。 申请 检查: 书写:审 核:

****此报告仅供本院医生参考，不作证明用*****
