# Supplementary material for: Augmenting Large Language Model With Prompt Engineering and Supervised Fine-Tuning in Non-Small Cell Lung Cancer Tumor-Node-Metastasis Staging: Framework Development and Validation
Source: JMIR AI. 2026 Apr 15;5:e77988. doi: 10.2196/77988 (PMC13082344; doi:10.2196/77988)
Supplement: Multimedia Appendix 6 [file ai-v5-e77988-s006.docx]

## Sample of T Staging

//【DATA INPUT】

输入：

# "任务":

具有专业医师的推理和判断能力，根据影像报告的内容，进行T分期。

# "影像报告":

病历号： 姓名： 性别： 男 年龄： 69 岁

科别：呼吸内科/呼吸与危重症医学

床号： 检查号： 检查日期：2024-061

科(钱塘)

核素：18F 药物：FDG 注射时间：2024-061 显像时间：2024-061

给药途径：静脉注射 血糖：5.6 检查项目：18F-FDG全身断层显像

临床诊断：肺部阴影

病史及检查目的：

2024-06-07 我院胸部CT 增强提示：左肺上叶纵隔胸膜下团块灶伴邻近 T4 椎体骨质破坏，MT 首

先考虑，建议进一步检查。

现为评估全身情况行PET/CT 检查。

检查所见：

检查过程：

空腹6h 以上，静脉注射18F-FDG50-60min 后行全身PET/CT 断层显像，影像清晰。检查范围

从头颅至股骨上1/3 处。

图像所见：

头部：右侧颞叶局部见等密度结节，边界欠清，较大截面约0.6cm，FDG 代谢增高，

SUVmax 为12.65，病灶周边可见水肿带，余大脑各叶、双侧基底节、丘脑、小脑及脑干密度、

形态未见明显异常，放射性分布均匀、对称。诸脑室、脑池、脑沟、脑裂轻度增宽、扩张。中线

结构居中。双侧额窦、蝶窦、筛窦及上颌窦粘膜未见明显增厚，放射性分布未见异常。

颈部：鼻咽顶后壁及双侧壁未见明显增厚，咽隐窝及咽旁间隙清晰，放射性分布未见明显

异常。口咽及喉咽密度未见明显异常，双侧咽淋巴环FDG 代谢增高，SUVmax 为5.34。双叶甲状

腺密度欠均，内可见多发低密度灶，大者位于左叶甲状腺上极，大小约0.7cm，FDG 代谢未见明

显增高。左侧锁骨上区见肿大淋巴结影，FDG 代谢增高，SUVmax 为3.95，右颈内静脉约C6-7 水

平局部条状FDG 代谢增高，SUVmax 为4.47，余双侧颌下、颈部及锁骨上淋巴结未见明显肿大及

异常放射性摄取。

胸部：双肺纹理增多，两肺多发囊性透亮影，左肺上叶尖后段近纵隔胸膜下见软组织密度

灶，较大截面约3.2×2.0cm，可见分叶、毛刺、胸膜凹陷、充气支气管征象，FDG 代谢增高，

SUVmax 为16.3，邻近 T4 椎体骨质破坏并软组织形成，FDG 代谢增高，SUVmax 为20.5；余两肺

可见多发结节（SE3IM 右 131、138、151、159，左 117），大者约0.9cm，部分 FDG 代谢轻度增

高，SUVmax 为0.90；两肺另见少许条索影。纵隔（2、3A、4、5、8 区）、左肺门、右侧內乳区

见多发大小不等淋巴结影，FDG 代谢不同程度增高，SUVmax 为12.83，双侧腋窝淋巴结未见明

显肿大及异常放射性摄取。食管管壁未见明显增厚及异常放射性分布。

腹部：肝脏形态、大小未见明显异常，肝VIII 段近膈面见小囊性低密度灶，大小约

0.8cm，FDG 代谢轻度增高，SUVmax 为2.42，余肝密度及放射性分布未见明显异常；肝内、外

胆管未见扩张。胆囊壁未见异常增厚，其内未见明显异常密度影及放射性分布异常增高。胃壁未

见明显增厚及异常放射性摄取增高。脾脏大小正常，放射性分布均匀。胰腺形态、大小、密度未

见明显异常，胰管未见扩张，未见异常放射性分布。右侧肾上腺略增粗，FDG 代谢增高，

本报告仅供本院医生参考，不作证明用。

1 / 2

病历号： 姓名： 性别： 男 年龄： 69 岁

SUVmax 为7.86，左侧肾上腺形态及放射性分布未见明显异常。右肾中极见囊性低密度灶，大小

约1.2cm，FDG 代谢缺失，双肾见点状致密影，双侧肾盂肾盏未见明显扩张，放射性分布未见异

常增高。肠道各段放射性分布未见明显异常增高。肝胃间隙见中小淋巴结影，FDG 代谢轻度增高，

SUVmax 为3.17，后腹膜近 L5 水平左侧见饱满淋巴结影,FDG 代谢增高，SUVmax 为6.51，余腹腔

内及腹膜后未见明显肿大淋巴结及异常放射性分布。

盆腔：膀胱充盈良好，膀胱壁未见明显增厚，内未见异常密度影。前列腺增生伴点状钙化，

未见FDG 异常摄取。双侧精囊腺未见FDG 异常摄取。双侧腹股沟未见明显肿大淋巴结及异常放

射性分布。

其他：多椎体及附件见骨质增生、硬化，部分伴骨赘形成；余所见颅骨、各躯干骨以及四

肢骨密度及放射性分布未见异常。

诊断意见：

1.a.左肺上叶尖后段肿块伴邻近 T4 骨质破坏，FDG 代谢增高，符合MT 表现；

b.纵隔（2、3A、4、5、8 区）、左肺门、右侧內乳区、左侧锁骨上区、左侧腹膜后大小不

等淋巴结，转移需考虑；

c.右侧肾上腺略增粗，FDG 代谢增高，转移可能；

d.右侧颞叶等密度结节伴周围水肿，FDG 代谢增高，转移考虑；

2.双叶甲状腺低密度结节；右颈内静脉局部 FDG 代谢增高，不除外生理性摄取可能；

3.两肺小结节，部分 FDG 代谢轻度增高，建议定期复查；两肺慢支肺气肿；左肺多发肺大泡；

两肺少许纤维灶；

4.肝 VIII 段近膈面小囊性灶，FDG 代谢轻度增高，建议定期复查；肝胃间隙中小淋巴结，

FDG 代谢轻度增高，倾向炎性反应增生改变，建议复查；

5.双肾结石，右肾囊肿；前列腺增生伴钙化；

6.椎体退行性改变；

7.老年脑改变。

报告医生： 审核医生： 报告时间：

2 / 2

//【Prompt for T Staging】

# "原发肿瘤判断标准":

1. 肿瘤的最大径："影像报告"可能会存在因为手术、治疗等原因，有多期肿瘤尺寸数据，肿瘤的最大径要治疗后最新的数据尺寸，注意，不要被“实性结节”等非肿瘤结节的尺寸信息误导。

2. 肿瘤侵犯：报告中有明确类似明确表达："椎骨转移"、"侵占"、"侵入"、"占位"。其他表达都不可作为侵犯判断标准。

3. 出现癌结节：报告中有明确的"恶性软组织"或同等严重性的描述，才可考虑癌结节，例如："右肺中叶恶性软组织肿块，伴双肺多发结节，考虑转移"。其他非同等严重性表达均不可作为出现癌结节的判断标准。

4. 有肿瘤导致的延伸至肺门区域的肺不张，或阻塞性肺炎：判断标准可参考"左肺上叶中央型肺Ca伴阻塞性肺不张"。

# "报告提取结果":

从影像报告中提取以下信息并带有判断依据：

- 肿瘤大小及位置：

肿瘤的最大径是多少mm？

肿瘤是否跨叶生长？

肿瘤位于哪个肺叶？

- 癌结节情况：

癌结节的位置和大小（单位mm）

是否同侧不同肺叶出现癌结节，给出判断依据和原因。

是否同一肺叶出现孤立性癌结节，给出判断依据和原因。

- T4分期侵入部位：

报告是否明确表明肿瘤侵犯了以下结构：膈肌、纵隔、心脏、大血管、气管、喉返神经、食管、椎体、隆突，给出判断依据和原因。

- T3分期侵入部位：

报告是否明确表明肿瘤侵犯了壁层胸膜[通常报告在病理染色中]、胸壁（包括肺上沟瘤）、膈神经、心包，给出判断依据和原因。

报告是否明确表明肿瘤侵犯了较远端的动脉或静脉的主干，给出判断依据和原因。

- T2分期侵入部位：

报告是否明确表明肿瘤侵犯了左或右主支气管或脏层胸膜，给出判断依据和原因。

# "T分期判断标准和工作流程":

Step 1: 基于"影像报告"，严格按照"原发肿瘤判断标准"，得出"报告提取结果"。

Step 2：符合以下任何一个Condition，则判断为"T4期"。如果没有，执行Step3。

Condition1. 当50mm<肿瘤最大<70mm，并且跨叶生长，结论为"T4期";

Condition2. 当肿瘤侵犯"T4分期侵入部位"任一结构时，结论为"T4期";

Condition3. 同侧不同肺叶出现癌结节，结论为"T4期"。

Step 3: 符合以下任何一个Condition，则判断为"T3期"。如果没有，执行Step4。

Condition1. 肿瘤侵犯"T3分期侵入部位"任一结构时，结论为"T3期"。

Condition2. 同一肺叶出现孤立性癌结节，结论为"T3期"。

Condition3. 肿瘤最大径小于50mm, 并且跨叶生长，结论为"T3期"。

Step 4: 符合下列Condition，则判断为相得出对应具体T2分期结论。如果没有，执行Step5。

Condition1. "影像报告"表达有肿瘤导致的延伸至肺门区域的肺不张或阻塞性肺炎，结论为"T2期";

Step 5: 如果不符合以上情况中的任何一种且有提取到肿瘤最大径数值，请根据提取到的肿瘤最大径数值，结合给定范围区间，对应到相应的分期结果，规则如下。

肿瘤最大径小于10mm，结论为"T1a期"

肿瘤最大径大于10mm且小于或等于20mm，结论为"T1b期"

肿瘤最大径大于20mm且小于或等于30mm，结论为"T1c期"

肿瘤最大径大于30mm且小于或等于40mm，结论为"T2a期"

肿瘤最大径大于40mm且小于或等于50mm，结论为"T2b期"

肿瘤最大径大于50mm且小于或等于70mm，结论为"T3期"

肿瘤最大径大于70mm，结论为"T4期"

Step 6: 因为术后等原因，导致无原发肿瘤信息，无法分期，结论为"Tx期"。

Step 7: 如果没有提到原发肿瘤信息，结论为"T0期";

# "输出格式":

{

"报告提取结果":{

"肿瘤大小及位置":{

"肿瘤的最大径(mm)":"",

"肿瘤是否跨叶生长":"",

"肿瘤位于哪个肺叶":""},

"癌结节情况":{

"癌结节的位置和大小(mm)":"",

"是否同侧不同肺叶出现癌结节":"",

"是否同一肺叶出现孤立性癌结节":""},

"T4分期侵入部位":"",

"T3分期侵入部位":"",

},

"T分期":{

"推理过程":"<按照 T分期判断标准和流程 得出的推理过程>",

"推理结论":"<你给出的判断T分期>"

}

}

# "要求":

1. 严格按照 "T分期判断标准和工作流程"的步骤，逐步执行，直到给出结论终止。

2. 深刻理解影像报告内容所表达的含义，对于报告中的“诊断意见”要重点参考。这直接关系到T分期的准确性。

3. 严格按照 "输出格式" 进行JSON输出,不带注释。其中"报告提取结果"的内容要按照"信息要求"提取于病人"影像报告"，"推理过程"要严格按照"T分期判断标准和流程",并且要写清楚为什么给出这个结论；"推理结论"表达要按要求，统一简洁。

4. 严谨对待Step 5，肿瘤最大径数值不要对应错分期结论。

5. 如果依据规则病人报告同时符合两个及以上分期结果，请将最严重分期作为最终结果。

# "输出":

//【OUTPUT】

输出：

{

"报告提取结果": {

"肿瘤大小及位置": {

"肿瘤的最大径(mm)": "32",

"肿瘤是否跨叶生长": "否",

"肿瘤位于哪个肺叶": "左肺上叶"

},

"癌结节情况": {

"癌结节的位置和大小(mm)": "无",

"是否同侧不同肺叶出现癌结节": "否",

"是否同一肺叶出现孤立性癌结节": "否"

},

"T4分期侵入部位": "T4椎体",

"T3分期侵入部位": "无"

},

"T分期": {

"推理过程": "根据影像报告，肿瘤最大径为32mm，位于左肺上叶，未跨叶生长。报告明确表明肿瘤侵犯了T4椎体，符合T4期的判断标准。因此，根据T分期判断标准和流程，患者分期为T4期。", //【reasoning traces】

//【Result】

"推理结论": "T4期"

}

}

//【Ground Truth】annotation: T4
