# Supplementary material for: Augmenting Large Language Model With Prompt Engineering and Supervised Fine-Tuning in Non-Small Cell Lung Cancer Tumor-Node-Metastasis Staging: Framework Development and Validation
Source: JMIR AI. 2026 Apr 15;5:e77988. doi: 10.2196/77988 (PMC13082344; doi:10.2196/77988)
Supplement: Multimedia Appendix 8 [file ai-v5-e77988-s008.docx]

## Sample of M Staging

//【DATA INPUT】

输入：

"Report"

病历号： 姓名： 性别： 男 年龄： 69 岁

科别：呼吸内科/呼吸与危重症医学

床号： 检查号： 检查日期：2024-061

科(钱塘)

核素：18F 药物：FDG 注射时间：2024-061 显像时间：2024-061

给药途径：静脉注射 血糖：5.6 检查项目：18F-FDG全身断层显像

临床诊断：肺部阴影

病史及检查目的：

2024-06-07 我院胸部CT 增强提示：左肺上叶纵隔胸膜下团块灶伴邻近 T4 椎体骨质破坏，MT 首

先考虑，建议进一步检查。

现为评估全身情况行PET/CT 检查。

检查所见：

检查过程：

空腹6h 以上，静脉注射18F-FDG50-60min 后行全身PET/CT 断层显像，影像清晰。检查范围

从头颅至股骨上1/3 处。

图像所见：

头部：右侧颞叶局部见等密度结节，边界欠清，较大截面约0.6cm，FDG 代谢增高，

SUVmax 为12.65，病灶周边可见水肿带，余大脑各叶、双侧基底节、丘脑、小脑及脑干密度、

形态未见明显异常，放射性分布均匀、对称。诸脑室、脑池、脑沟、脑裂轻度增宽、扩张。中线

结构居中。双侧额窦、蝶窦、筛窦及上颌窦粘膜未见明显增厚，放射性分布未见异常。

颈部：鼻咽顶后壁及双侧壁未见明显增厚，咽隐窝及咽旁间隙清晰，放射性分布未见明显

异常。口咽及喉咽密度未见明显异常，双侧咽淋巴环FDG 代谢增高，SUVmax 为5.34。双叶甲状

腺密度欠均，内可见多发低密度灶，大者位于左叶甲状腺上极，大小约0.7cm，FDG 代谢未见明

显增高。左侧锁骨上区见肿大淋巴结影，FDG 代谢增高，SUVmax 为3.95，右颈内静脉约C6-7 水

平局部条状FDG 代谢增高，SUVmax 为4.47，余双侧颌下、颈部及锁骨上淋巴结未见明显肿大及

异常放射性摄取。

胸部：双肺纹理增多，两肺多发囊性透亮影，左肺上叶尖后段近纵隔胸膜下见软组织密度

灶，较大截面约3.2×2.0cm，可见分叶、毛刺、胸膜凹陷、充气支气管征象，FDG 代谢增高，

SUVmax 为16.3，邻近 T4 椎体骨质破坏并软组织形成，FDG 代谢增高，SUVmax 为20.5；余两肺

可见多发结节（SE3IM 右 131、138、151、159，左 117），大者约0.9cm，部分 FDG 代谢轻度增

高，SUVmax 为0.90；两肺另见少许条索影。纵隔（2、3A、4、5、8 区）、左肺门、右侧內乳区

见多发大小不等淋巴结影，FDG 代谢不同程度增高，SUVmax 为12.83，双侧腋窝淋巴结未见明

显肿大及异常放射性摄取。食管管壁未见明显增厚及异常放射性分布。

腹部：肝脏形态、大小未见明显异常，肝VIII 段近膈面见小囊性低密度灶，大小约

0.8cm，FDG 代谢轻度增高，SUVmax 为2.42，余肝密度及放射性分布未见明显异常；肝内、外

胆管未见扩张。胆囊壁未见异常增厚，其内未见明显异常密度影及放射性分布异常增高。胃壁未

见明显增厚及异常放射性摄取增高。脾脏大小正常，放射性分布均匀。胰腺形态、大小、密度未

见明显异常，胰管未见扩张，未见异常放射性分布。右侧肾上腺略增粗，FDG 代谢增高，

本报告仅供本院医生参考，不作证明用。

1 / 2

病历号： 姓名： 性别： 男 年龄： 69 岁

SUVmax 为7.86，左侧肾上腺形态及放射性分布未见明显异常。右肾中极见囊性低密度灶，大小

约1.2cm，FDG 代谢缺失，双肾见点状致密影，双侧肾盂肾盏未见明显扩张，放射性分布未见异

常增高。肠道各段放射性分布未见明显异常增高。肝胃间隙见中小淋巴结影，FDG 代谢轻度增高，

SUVmax 为3.17，后腹膜近 L5 水平左侧见饱满淋巴结影,FDG 代谢增高，SUVmax 为6.51，余腹腔

内及腹膜后未见明显肿大淋巴结及异常放射性分布。

盆腔：膀胱充盈良好，膀胱壁未见明显增厚，内未见异常密度影。前列腺增生伴点状钙化，

未见FDG 异常摄取。双侧精囊腺未见FDG 异常摄取。双侧腹股沟未见明显肿大淋巴结及异常放

射性分布。

其他：多椎体及附件见骨质增生、硬化，部分伴骨赘形成；余所见颅骨、各躯干骨以及四

肢骨密度及放射性分布未见异常。

诊断意见：

1.a.左肺上叶尖后段肿块伴邻近 T4 骨质破坏，FDG 代谢增高，符合MT 表现；

b.纵隔（2、3A、4、5、8 区）、左肺门、右侧內乳区、左侧锁骨上区、左侧腹膜后大小不

等淋巴结，转移需考虑；

c.右侧肾上腺略增粗，FDG 代谢增高，转移可能；

d.右侧颞叶等密度结节伴周围水肿，FDG 代谢增高，转移考虑；

2.双叶甲状腺低密度结节；右颈内静脉局部 FDG 代谢增高，不除外生理性摄取可能；

3.两肺小结节，部分 FDG 代谢轻度增高，建议定期复查；两肺慢支肺气肿；左肺多发肺大泡；

两肺少许纤维灶；

4.肝 VIII 段近膈面小囊性灶，FDG 代谢轻度增高，建议定期复查；肝胃间隙中小淋巴结，

FDG 代谢轻度增高，倾向炎性反应增生改变，建议复查；

5.双肾结石，右肾囊肿；前列腺增生伴钙化；

6.椎体退行性改变；

7.老年脑改变。

报告医生： 审核医生： 报告时间：

本报告仅供本院医生参考，不作证明用。

2 / 2

//【Prompt for M Staging】

"Duty"

甄别出Report中M分期有效的信息，专注判断Metastasis stage，排除N分期(区域淋巴结转移)信息的干扰。

"Background Knowledge"

Knowledge1: 出现“小结节”、“低密度结节影”、“致密影”、“高密度影”、“强回声伴声影”、“软化灶”、“条状强化影”等表达时，严谨考虑，不能直接推断为转移性病灶。

Knowledge2: 其他远处器官有“转移性xxx”、“考虑转移”、“疑似转移”、“密度增高影”、“高密度结节”等明确的表达，可以作为转移病灶的依据。

"M分期判断标准"：

Criteria1. 如果报告中注明：多个器官出现病灶，或者单个器官出现多处病灶，判断为M1c。

Criteria2. 如果报告中注明：单个器官的单处病灶，判断为M1b。

Criteria3. 如果只有胸腔内转移，报告明确说明：对侧肺出现转移性癌结节（单个或多个），判断为M1a。

Criteria4. 如果只有胸腔内转移，报告明确说明：肿瘤伴有胸膜，或者心包转移性癌结节，判断为M1a。

Criteria5. 如果只有胸腔内转移，且PET/穿刺病理报告中出现：恶性胸腔积液，或者心包积液，判断为M1a。

Criteria6. 如果无明显远处转移依据，判断为M0。

Criteria7. 如果未见其他胸腔外脏器检查报告，或无法判定，判断为Mx。

"Work Flow"

Step1. 注入Background Knowledge

Step2. 根据M分期标准判断

"问题"：

M分期包括：M1a,M1b,M1c,M0,Mx.

判断严格满足的M分期判断标准，给出M分期判断的依据，给出M分期结论。结论格式{xx}

//【OUTPUT】

输出

根据分期判断标准

```json

{//【reasoning traces】

根据提供的CT和MR影像学报告单，我们可以按照M分期判断标准来分析：

1. 报告中提到“右侧肾上腺略增粗，FDG 代谢增高，转移可能；”，这是一个远处器官的转移性病灶。

2. 报告中还提到“右侧颞叶等密度结节伴周围水肿，FDG 代谢增高，转移考虑”，这也是一个远处器官的转移性病灶。

根据Knowledge2，如果报告中注明多个器官出现病灶，可以判断为M1c。

//【Result】

因此，结论是：{M1c}

}

```

//【Ground Truth】annotation: M1c
