# Supplementary material for: Augmenting Large Language Model With Prompt Engineering and Supervised Fine-Tuning in Non-Small Cell Lung Cancer Tumor-Node-Metastasis Staging: Framework Development and Validation
Source: JMIR AI. 2026 Apr 15;5:e77988. doi: 10.2196/77988 (PMC13082344; doi:10.2196/77988)
Supplement: Multimedia Appendix 9 [file ai-v5-e77988-s009.docx]

Model Card

1. Model Specifications

Model Name: Lung Cancer TNM Staging Model

Version: v1.0

Architecture: Fine-tuned model based on GLM-4-Air, with 32 billion parameters

Language: Simplified Chinese

2. Intended Use

This model is designed as an auxiliary clinical decision support tool. Its outputs are intended to serve as supplementary reference information, aiming to enhance diagnostic workflow efficiency and consistency. Specifically, the model is designed to:

Automatically extract TNM staging components from structured or semi-structured lung cancer medical imaging reports.

Generate corresponding TNM and clinical staging suggestions.

Support radiologists and oncologists in rapidly identifying key staging information.

It cannot replace the professional judgment of licensed physicians.

3. Training Data

Dataset Composition: The model was trained and validated using 492 rigorously de-identified lung cancer diagnostic reports. The imaging modality distribution was 84.76% CT, 13.01% PET-CT, with the remainder comprising other modalities (e.g., MRI, Ultrasound). Regarding hospital sources, 78% of cases originated from 51 distinct contributing hospitals, while the source was unidentified for the remaining 22% due to users' proactive removal of hospital identifiers for privacy protection during upload.

Data Sources: All included cases had undergone relevant clinical management between January 2018 and May 2025, with available medical records containing extractable key elements essential for TNM staging, such as tumor size, location, nodal status, and metastatic findings.

Data Quality & Pre-processing:

(1) De-identification: All reports underwent strict de-identification combining automated processes and manual sampling checks. Direct identifiers (e.g., patient name, ID number, admission number) were removed, and institutional identifiers (e.g., hospital names, department seals) were removed or blurred to comply with medical data privacy and security standards.

(2) Annotation Pipeline: TNM staging labels were independently annotated by two experienced physicians using a double-blind method. For cases with discrepant annotations, a third senior expert served as an arbitrator to ensure gold-standard label quality.

4. Quantitative Performance Summary

Model performance was evaluated on an independent test set. Key metrics include Precision, Recall, and *F*_1_-score for the T, N, and M components.

| Evaluation Task | Accuracy  (95% CI) | Precision  (Macro-  averaging) | Recall  (Macro-  averaging) | *F*_1_-score  (Macro-  averaging) |
| --- | --- | --- | --- | --- |
| T stage | 92.0%  (85.0% - 95.9%) | 92.60% | 91.40% | 0.914 |
| N stage | 86.0%  (77.9% - 91.5%) | 84.20% | 86.70% | 0.815 |
| M stage | 92.0%  (85.0% - 95.9%) | 82.60% | 86.50% | 0.831 |

5. Ethical Considerations

Data Privacy & Compliance: The model development strictly adheres to medical data ethics guidelines. All training data have been de-identified. The data processing and storage procedures comply with China's "Personal Information Protection Law" and relevant medical information security standards, and have obtained approval from the relevant institutional ethics committee.

Human-in-the-loop: This model is designed as an assistive tool (Clinical Decision Support). Final diagnosis must be confirmed by a licensed physician considering the patient's complete clinical information.

Fairness: To avoid exacerbating healthcare resource inequality, technical optimizations ensure the model can run efficiently on consumer-grade GPU hardware, lowering the barrier to deployment.

Licensing: The training dataset does not incorporate, nor was it trained on, any copyrighted material without proper authorization.

6. Limitations

Performance Boundary: Model performance may degrade for specific patient subgroups or rare case types not sufficiently covered in the training data.

Domain Specificity: Exclusively validated on lung cancer imaging reports; performance not guaranteed for other malignancies (e.g., breast or colorectal cancers).

Text-Dependent Performance: Accuracy requires explicit documentation of anatomical locations, lesion dimensions, nodal status, and metastatic findings; ambiguous or incomplete descriptions may compromise staging reliability.

7. Failure Modes

The model may return 'Undetermined' or incomplete staging conclusions for T/N/M components when corresponding clinical evidence is absent or only implicitly described in the report text.
